# Supplementary material for: Unraveling the Neuropharmacological Properties of Lippia alba: A Scientometric Approach
Source: Pharmaceuticals (Basel). 2025 Mar 16;18(3):420. doi: 10.3390/ph18030420 (PMC11945933; doi:10.3390/ph18030420)
Supplement: Supplementary file 1 [file pharmaceuticals-18-00420-s001.zip › pharmaceuticals-3424184-supplementary.pdf]

SUPPLEMENTARY INFORMATION

Unraveling the neuropharmacological properties of Lippia alba: a scientometric approach

Pedro I. C. Silva [1,3,5], Lucas V. P. S. Pantoja [1,2], Brenda C. Conceição [1,2], Marta E. O. Barbosa [2,3], Luiza F. R. Soares [3], Rui Daniel Prediger [4], Enéas A. Fontes-Júnior [3], Jofre J. S. Freitas [5]* and Cristiane S. F. Maia [3]*

[1]Programa de Pós-Graduação em Farmacologia e Bioquímica, Universidade Federal do Pará, 66075-900 Belém, PA, Brazil;
[2]Programa de Pós-Graduação em Ciências Farmacêuticas, Universidade Federal do Pará, 66075-900 Belém, PA, Brazil;
[3]Laboratório de Farmacologia da Inflamação e do Comportamento, Instituto de Ciências da Saúde, Universidade Federal do Pará, 66075-900 Belém, PA, Brazil;
[4]Laboratório Experimental de Doenças Neurodegenerativas, Departamento de Farmacologia, Centro de Ciências Biológicas, Universidade Federal de Santa Catarina, 88049-900 Florianópolis, SC, Brazil;
[5]Centro de Estudos Pré-Clínicos da Amazônia, Universidade do Estado do Pará, Belém, 66087-662 Belém, PA, Brazil.

** Corresponding author: Cristiane Socorro Ferraz Maia and Jofre J. S. Freitas*

LIST OF CONTENTS

Table S1. List of Excluded Articles.---Page 3
Table S2. List of Journals.--Page 50
Table S3. Science mapping of Lippia alba neuropharmacological research. ------------Page 51
Table S4. Phytochemical aspects of *Lippia alba*.--Page 66

Page 3. Supplementary Information – Table S1. List of excluded articles.

Authors	Article Title	Journal	WoS-CC citations	Reason for exclusion
Sena, JG, Duringer, JM; Souza, IA ; da Cunha, EVL; Craig, AM; Silva, MS; Barbosa, JM; Xavier, HS	Phytochemistry And Acute Toxicity From The Roots Of *Lippia alba*	Pharmaceutical Biology	0	Phytochemical study
Oliveira, DR; Leitao, GG; Santos, SS; Bizzo, HR; Lopes, D; Alviano, CS; Alviano, DS; Leitao, SG	Ethnopharmacological Study Of Two *Lippia* Species From Oriximina, Brazil	Journal of Ethnopharmacology	73	Ethnopharmacological study
Barbosa, FG ; Lima, MAS ; Braz-Filho, R ; Silveira, ER	Iridoid And Phenylethanoid Glycosides From *Lippia Alba*	Biochemical Systematics And Ecology	18	Biochemical study
Trevisan, MTS; Marques, RA; Silva, MGV; Scherer, D; Haubner, R; Ulrich, CM; Owen, RW	Composition Of Essential Oils And Ethanol Extracts Of The Leaves Of *Lippia* Species: Identification, Quantitation And Antioxidant Capacity	Records of Natural Products	19	Phytochemical study
Hedhili, L	*Ippia Alba* Essential Oil: A Promising Complementary Therapy for Breast Cancer	Chemistry & Biodiversity	0	Focus on other areas of study
Fischer, U; Lopez, R; Pöll, E; Vetter, S; Novak, J; Franz, CM	Two chemotypes within *Lippia alba* populations in Guatemala	Flavour And Fragrance Journal	13	Phytochemical study
de Araújo, AKO; Gomes, RDS; da Silva, MLM; Santos, AMD; do Nascimento, LC	Sanitary and physiological quality of *Chorisia glaziovii* O. Kuntze seeds treated with plant extracts	Ciência Florestal	0	Focus on Agricultural Sciences
Tavares, IB; Momnté, VG; Barreto, HG; de Castro, HG; do Santos, GR; do Nascimento, IR	Types Of Cuttings And Different Substrates In The Vegetative Propagation Of The *Lippia alba* (Chemotypes I, II And III)	Bioscience Journal	4	Phytochemical study
Rocha, DS; Santos, CP; Bajay, MM; Campos, JB; Blank, AF; Pinheiro, JB; Zucchi, MI	Development of a novel set of microsatellite markers for *Lippia alba* (Verbenaceae)	Genetics And Molecular Research	4	Phytochemical study
Rameshkumar, A; Sardar, S; Majumder, B; Dey, S; Kazmi, SI	Observation of bee pollinators (Apoidea) on a medicinal plant, *Lippia alba* (Mill.) (Verbenaceae)	Current Science	0	Focus on other areas of study
Timóteo, P; Karioti, A; Leitao, SG ; Vincieri, FF; Bilia, AR	HPLC/DAD/ESI-MS Analysis of Non-volatile Constituents of Three Brazilian Chemotypes of *Lippia alba* (Mill.) N. E. Brown	Natural Product Communications	8	Phytochemical study
de Oliveira, GT; Ferreira, JMS; Rosa, LH; de Siqueira, EP; Johann, S; Lima, LARD	*In vitro* Antifungal Activities Of Leaf Extracts Of *Lippia alba* (Verbenaceae) Against Clinically Important Yeast Species	Revista Da Sociedade Brasileira De Medicina Tropical	11	Focus on other areas of study

Authors	Article Title	Journal	WoS-CC citations	Reason for exclusion
Hennebelle, T; Sahpaz, S; Joseph, H; Bailleul, F.	Phenolics and iridoids of *Lippia alba*	Natural Product Communications	15	Phytochemical study
Kampke, EH; Barroso, MED; Marques, FM; Fronza, M; Scherer, R; Lemos, MF; Campagnaro, BP; Gomes, LC	Genotoxic Effect Of *Lippia alba (*Mill.) N. E. Brown Essential Oil On Fish (*Oreochromis niloticus)* And Mammal (*Mus musculus*)	Environmental Toxicology And Pharmacology	16	Focus on other areas of study
Becker, AJ; Braga, A; Magalha, V ; Medeiros, LM; Ramos, PB ; Monserrat, JM; Heinzmann, BM; Schmidt, D; Wasielesky, W Jr; Baldisserotto, B	Examination of the antioxidant effects of linalool and *Lippia alba* essential oil in the Pacific whiteleg shrimp, *Litopenaeus vannamei* , subjected to eyestalk ablation	Aquaculture	0	Focus on other areas of study
Sousa, SM; Silva, PS; Torres, GA ; Viccini, LF	Chromosome banding and essential oils composition of Brazilian accessions of Lippia alba (Verbenaceae)	Biologia	12	Phytochemical study
Lorenzo, D; Paz, D; Davies, P ; Vila, R ; Cañigueral, S; Dellacassa, E	Composition of a new essential oil type of *Lippia alba* (Mill.) NE Brown from Uruguay	Flavour And Fragrance Journal	25	Phytochemical study
da Silva, NIS; de Moura, JFP; Nascimento, MED; Machado, FCG ; Costa, TGP ; de Araujo, JM; Corda, MA; Edvan, RL; Oliveira, RL; Bezerra, LR	Effect of *Lippia alba* hay as phytogenic feed additive on the lactation performance, milk composition, and rumen and blood parameters of *Alpine goats*	Small Ruminant Research	2	Focus on other areas of study
Bahl, JR; Garg, SN; Singh, SC; Bansal, RP; Naqvi, AA; Kumar, S	Composition of linalool rich essential oil from *Lippia alba* grown in Indian plains	Flavour And Fragrance Journal	14	Phytochemical study
Farias, MR; Pértile, R; Correa, MM; de Almeida, MTR; Palermo, JA; Schenkel, EP	Triterpenoid Saponins from *Lippia alba* (Mill.) N. E. Brown	Journal Of The Brazilian Chemical Society	05	Phytochemical study
Montero, VS; Rodenak, KB; Polo, MP; Cicció, AJF; García, DBMM	Synergism of *Lippia alba* essential oils with statins in antiproliferative and hypolipogenic effects	Biocell	0	Focus on other areas of study
de Barros, FMC; Zambarda, ED; Heinzmann, BM; Mallmann, CA	Seasonal variability and terpenoid biosynthesis of the essential oil of *Lippia alba* (mill.) N. E. Brown (verbenaceae)	Quimica Nova	28	Phytochemical study
Barbosa, FG; Lima, MAS; Silveira, ER	Total NMR assignments of new[C7-O-C7"]-biflavones from leaves of the limonene-carvone chemotype of *Lippia alba* (Mill) N.E. Brown	Magnetic Resonance In Chemistry	16	Phytochemical study

Authors	Article Title	Journal	WoS-CC citations	Reason for exclusion
Barbosa, FDF; Barbosa, LCA; Melo, EC; Botelho, FM; Santos, RHS	Effect of drying air temperature upon the content and chemical composition of the essential oil from *Lippia alba* (Mill) N. E.!Brown.	Química Nova	22	Phytochemical study
de Queiroz, TB; Dos Santos, JC; Neves, FTA; Bellini, MF	Physical - chemical analysis of *Lippia alba* and *Melissa officinalis* and clastogenicity evaluation of its aqueous extracts in human leukocytes	Anticancer Research	0	Focus on other areas of study
Gazola, R; Machado, D; Ruggiero, C; Singi, G; Alexandre, MM	*Lippia alba*, *Melissa officinalis* and *Cymbopogon citratus*:: effects of the aqueous extracts on the isolated hearts of rats	Pharmacological Research	37	Focus on other areas of study
Mendoza, JDS; Correia, LC; Saad, JCC; Siqueira, WJ; Ming, L; Campos, FG; Boaro, CSF; Marques, MOM	Effect of irrigation depth on biomass production and metabolic profile of *Lippia alba* (linalool chemotype) essential oil	Agricultural Water Management	03	Plant physiology study
Zoghbi, MDGB; Andrade, EHA; Santos, AS; Silva, MHL; Maia, JGS	Essential oils of *Lippia alba* (Mill.) N. E. Br growing wild in the Brazilian Amazon	Flavour And Fragrance Journal	43	Plant physiology study
Comes, AF; Ganzera, M; Schwaiger, S; Stuppner, H; Halabalaki, M; Almeida, MP; Leite, MF; Amaral, JG; David, JM	Simultaneous determination of iridoids, phenylpropanoids and flavonoids in *Lippia alba* extracts by micellar electrokinetic capillary chromatography	Microchemical Journal	19	Phytochemical study
Manica-Cattani, MF; Zacaria, J; Pauletti, G; Atti-Serafini, L; m Echeverrigaray, S	Genetic variation among South Brazilian accessions of *Lippia alba* Mill. (Verbenaceae) detected by ISSR and RAPD markers	Brazilian Journal Of Biology	28	Plant physiology study
dos Reis, PS; Estevam, IHS; dos Santos, WPC; Korn, MGA; David, JM; David, JP; Araújo, RGO; Pimentel, MF; Ferreira, SLC	Mineral Composition of *Lippia alba* (Mill.) N.E. Brown Leaves	Journal Of The Brazilian Chemical Society	09	Phytochemical study
Timóteo, P; Karioti, A; Leitao, SG; Vincieri, FF; Bilia, AR	Polyphenol content of aqueous preparations of three chemotypes of *Lippia alba* Mill N. E. Brown (Verbenaceae) by HPLC/DAD/ESI-MS	Planta Médica	0	Phytochemical study
Niculau, ED ; Alves, PB ; Nogueira, PCD ; Moraes, VRD ; Matos, AP; Bernardo, AR ; Volante, AC ; Fernandes, JB ; da Silva, MFGF; Correa, AG; Blank, AF ; Silva, AD; Ribeiro, LD	Insecticidal activity of essential oils of *Pelargonium graveolens* l'Herit and *Lippia alba* (Mill) N. E. Brown against *Spodoptera frugiperda* (J. E. Smith)	Química Nova	18	Bioplaguicide study

Authors	Article Title	Journal	WoS-CC citations	Reason for exclusion
Agatonovic-Kustrin, S; Kustrin, E; Gegechkori, V; Morton, DW	Anxiolytic Terpenoids and Aromatherapy for Anxiety and Depression	Reviews On New Drug Targets In Age-Related Disorders	36	*Lippia alba* is not the focus of the paper
Vishwakarma, P; Pandey, AK; Mishra, P; Singh, P; Tripathi, NN	Enhancement of Shelf Life of Button Mushroom, *Agaricus bisporus* (Higher Basidiomycetes) by Fumigant Application of *Lippia alba* Essential Oil	International Journal Of Medicinal Mushrooms	04	Focus on Agricultural Sciences
Sena, JG; Duringer, JM; Uchoa, DEA; Xavier, HS; Barbosa-Filho, JM; Braz-Filho, R	Distribution of iridoid glucosides in plants from the genus Lippia (Verbenaceae): An investigation of *Lippia alba* (Mill.) NE brown	Natural Product Communications	16	Phytochemical study
Sales, G; Medeiros, S; Soares, I; Sampaio, T; Bandeira, M; Nogueira, N; Queiroz, M	Antifungal and Modulatory Activity of Lemon Balm (*Lippia alba* (MILL.) N. E. BROWN) Essential Oil	Scientia Pharmaceutica	04	Focus on other areas of study
Santos, FRC; Lima, PF; Priolli, RHG; Siqueira, WJ; Colombo, CA	Isolation and characteristics of eight novel polymorphic microsatellite loci in *Lippia alba* (verbenaceae)	American Journal Of Botany	06	Botanical study
da Silva, ACC; Barbosa, FG; Mafezoli, J; de Oliveira, MDF; de Oliveira, TF	HS-SPME as an efficient tool for discriminating chemotypes of *Lippia alba* (Mill.) N. E. Brown	Química Nova	05	Phytochemical study
Kellner, AWA	Vegetation replacement in Rio de Janeiro during the last 7,000 years BC, genetic variation in the medicinal plant *Lippia alba* (Verbenaceae), and short review of Chagas disease in prehistory	Anais Da Academia Brasileira De Ciências	01	Focus on other areas of study
Nunes, MR; Castilho, MDM; Veeck, APD; da Rosa, CG; Noronha, CM; Maciel, MVOB; Barreto, PM	Antioxidant and antimicrobial methylcellulose films containing *Lippia alba* extract and silver nanoparticles	Carbohydrate Polymers	60	Focus on other areas of study
Lopes, JML; de Matos, EM ; Nascimento, LSD; Viccini, LF	Validation of reference genes for quantitative gene expression in the *Lippia alba* polyploid complex (Verbenaceae)	Molecular Biology Reports	08	Botanical study
Villamizar-Véliz, M; Aular, Y	Review of the extraction methods of the essential oil of *Lippia alba*	Ingenieria Uc	0	Phytochemical study
Gomes, AF; Almeida, MP; Leite, MF; Schwaiger, S; Stuppner, H; Halabalaki, M; Amaral, JG; David, JM	Seasonal variation in the chemical composition of two chemotypes of *Lippia alba*	Food Chemistry	52	Phytochemical study
Cardona, MJO; Muñoz, RJE	Genetic diversity and geographical genetic diversity in Colombian accessions of *Lippia alba* (Mill.) N.E. Brown	Revista De Ciências Agricolas	0	Botanical study

Authors	Article Title	Journal	WoS-CC citations	Reason for exclusion
Peixoto, MG; Costa, LM; Blank, AF; Lima, AD; Menezes, TSA; Santos, DD; Alves, PB; Cavalcanti, SCD; Bacci, L; Arrigoni-Blank, MD	Acaricidal activity of essential oils from *Lippia alba* genotypes and its major components carvone, limonene, and citral against *Rhipicephalus microplus*	Veterinary Parasitology	62	Bioplaguicide study
López, MA ; Stashenko, EE; Fuentes, JL	Chemical composition and antigenotoxic properties of *Lippia alba* essential oils	Genetics And Molecular Biology	45	Focus on other areas of study
Siani, AC; Tappin, MRR; Ramos, MFS; Mazzei, JL; Conceiçao, M; Ramos, KV; Neto, FRD; Frighetto, N	Linalool from *Lippia alba*:Study of the reproducibility of the essential oil profile and the enantiomeric purity	Journal Of Agricultural And Food Chemistry	32	Phytochemical study
de Oliveira, GT; Amado, PA; Ferreira, JMS; Lima, LARD	Allelopathic effect of the ethanol extract and fractions of the aerial parts of *Lippia alba* (Verbenaceae)	Natural Product Research	02	Botanical study
Tavares, ES; Lopes, D; Bizzo, HR; Lage, CLS; Leitao, SG	Kinetin enhanced linalool production by in vitro plantlets of *Lippia alba*	Journal Of Essential Oil Research	22	Phytochemical study
Yamari, I; Abchir, O; Siddique, F; Zaki, H; Errougui, A; Talbi, M; Bouachrine, M; Elkouali, M'; Chtita, S	The anticoagulant potential of *Lippia Alba* extract in inhibiting SARS-CoV-2 Mpro: Density functional calculation, molecular docking analysis, and molecular dynamics simulations	Scientific African	04	Focus on other areas of study
Ortiz, N; Jiménez, MF; Chaverri, C; Cicció, JF; Díaz, C	Effect on cell growth, viability and migration of geraniol and geraniol-containing essential oil from *Lippia alba* (Verbenaceae) on gastric carcinoma cells	Journal Of Essential Oil Research	07	Focus on other areas of study
da Silva, HNP; de Souza, RN; Sousa, EMD; Mourao, RHV; Baldisserotto, B; da Silva, LVF	Citral chemotype of the *Lippia alba* essential oil as an additive in simulated transport with different loading densities of tambaqui juveniles	Ciência Rural	02	Focus on other areas of study
Montero-Villegas, S; Crespo, R; Rodenak-Kladniew, B; Castro, MA; Galle, M; Cicció, JF; de Bravo, MG; Polo, M	Cytotoxic effects of essential oils from four *Lippia alba* chemotypes in human liver and lung cancer cell lines	Journal Of Essential Oil Research	08	Focus on other areas of study
Reis, AC; Sousa, SM; Vale, AA; Pierre, PMO; Franco, AL; Campos, JMS; Vieira, RF; Viccini, LF	*Lippia alba* (Verbenaceae): a new tropical autopolyploid complex?	American Journal Of Botany	30	Botanical study
de Oliveira, GT; Ferreira, JMS; Lima, WG; Alves, LF; Duarte-Almeida, JM; Lima, LARD	Phytochemical characterisation and bioprospection for antibacterial and antioxidant activities of *Lippia alba* Brown ex Britton & Wilson (Verbenaceae)	Natural Product Research	13	Focus on other areas of study

Authors	Article Title	Journal	WoS-CC citations	Reason for exclusion
Viccini, LF; Silveira, RS; do Vale, AA; de Campos, JMS; Reis, AC; Santos, MD; Campos, VR; Carpanez, AG; Grazul, RM	Citral and linalool content has been correlated to DNA content in *Lippia alba* (Mill.) NE Brown (Verbenaceae)	Industrial Crops And Products	27	Botanical study
de Jesus, RA; Prado, VMJ; Pinto, VS; Silva, VR; Santos, LS; Nogueira, PCL ; Navickiene, S; Pereira-Filho, ER; Blank, AE; Bezerra, DP; Soares, MBP; Seidl, C ; Cardoso, CL; Moraes, VRS	Application of LC-DAD Metabolic Fingerprinting in Combination with PCA for Evaluation of Seasonality and Extraction Method on the Chemical Composition of Accessions from *Lippia alba* (Mill.) N. E. Brown and Biological Activities	Journal Of The Brazilian Chemical Society	02	Focus on other areas of study
Castro, DM; Ming, LC; Marques, MOM	Biomass production and chemical composition of *Lippia alba* (Mill.) NEBr. ex Britt & Wilson in leaves on different plant parts in different seasons	Proceedings Of The First Latin-American Symposium On The Production Of Medicinal, Aromatic And Condiments Plants	05	Meeting Abstract
Marques, CTD; Gama, EVS; da Silva, F; Teles, S; Caiafa, AN; Lucchese, AM	Improvement of biomass and essential oil production of *Lippia alba* (Mill) NE Brown with green manures in succession	Industrial Crops And Products	19	Botanical study
Pandeló, D; Melo, TD; Singulani, JL; Guedes, FAF; Machado, MA; Coelho, CM; Viccini, LF; Santos, MO	Oil production at different stages of leaf development in *Lippia alba*	Brazilian Journal Of Pharmacognosy	10	Phytochemical study
Hennebelle, T; Sahpaz, S; Joseph, H; Bailleul, F	Ethnopharmacology of *Lippia alba*	Journal Of Ethnopharmacology	142	Ethnopharmacological study
Timóteo, P; Karioti, A; Leitao, SG; Vincieri, FF; Bilia, AR	A validated HPLC method for the analysis of herbal teas from three chemotypes of Brazilian *Lippia alba*	Food Chemistry	36	Phytochemical study
Malik, S; Odeyemi, S; Pereira, GC; de Freitas, LM; Abdul-Hamid, H; Atabaki, N; Makhzoum, A; de Almeida, EB; Dewar, J; Abiri, R	New insights into the biotechnology and therapeutic potential of *Lippia alba (*Mill.) NEBr. ex P. Wilson	Journal Of Essential Oil Research	10	Focus on other areas of study
Pierre, PMO; Sousa, SM; Davide, LC; Machado, MA; Viccini, LF	Karyotype analysis, DNA content and molecular screening in *Lippia alba (Verbenaceae)*	Anais Da Academia Brasileira De Ciências	24	Botanical study

Authors	Article Title	Journal	WoS-CC citations	Reason for exclusion
De Morais, SM; Sobrinho, ACN; Liberato, HR; Pereira, RDA Pessoa, C; Alves, DR; Fontanelle, ROD.	Biotechnological potential of essential oils from different chemotypes of *Lippia alba* (Mill.) NEBr. ex Britton & P. Wilson	Boletin Latinoamericano Y Del Caribe De Plantas Medicinales Y Aromaticas	03	Phytochemical study
Parra-Garcés, MI; Caroprese-Araque, JF; Arrieta-Prieto, D; Stashenko, E	Morphology, anatomy, ontogeny and chemical composition of inflorescences volatile secondary metabolites of *Lippia alba* (Verbenaceae) at three stages of development	Revista De Biologia Tropical	07	Botanical study
Ricciardi, G; Cicció, JF; Ocampo, R; Lorenzo, D; Ricciardi, A; Bandoni, A; Dellacassa, E	Chemical Variability of Essential Oils of *Lippia alba* (Miller) N. E. Brown Growing in Costa Rica and Argentina	Natural Product Communications	15	Phytochemical study
Senatore, F; Rigano, D.	Essential oil of two *Lippia* spp. (Verbenaceae) growing wild in Guatemala	Flavour And Fragrance Journal	34	Phytochemical study
Veras, HNH; Campos, AR; Rodrigues, FFG; Botelho, MA; Coutinho, HDM; da Costa, JGM	*Lippia alba* (Mill.) NE Essential Oil Interfere with Aminoglycosides Effect Against *Staphylococcus aureus*	Journal Of Essential Oil Bearing Plants	02	Focus on other areas of study
Gupta, SK; Khanuja, SPS; Kumar, S	In vitro micropropagation of *Lippia alba*	Current Science	24	Botanical study
Mesa-Arango, AC; Betancur-Galvis, L; Montiel, J ; Bueno, JG; Baena, A; Durán, DC; Martínez, JR; Stashenko, EE	Antifungal Activity and Chemical Composition of the Essential Oils of *Lippia alba* (Miller) N.E Brown Grown in Different Regions of Colombia	Journal Of Essential Oil Research	09	Focus on other areas of study
Bahl, JR; Sinha, S; Naqvi, AA; Bansal, RP; Gupta, AK; Kumar, S	Linalool-rich essential oil quality variants obtained from irradiated stem nodes in *Lippia alba*	Flavour And Fragrance Journal	12	Phytochemical study
Aguiar, JS; Costa, MCCD; Nascimento, SC; Sena, KXFR	Antimicrobial activity of *Lippia alba* (Mill.) N. E. Brown (Verbenaceae)	Brazilian Journal Of Pharmacognosy	37	Focus on other areas of study
García-Abarrio, SM; Martin, L; Burillo, J; Della Porta, G; Mainar, AM	Supercritical fluid extraction of volatile oil from *Lippia alba* (Mill.) cultivated in Aragon (Spain)	Journal Of Supercritical Fluids	12	Phytochemical study
Santos, MEAHP; Rodrigues, MS; Siqueira, WJ; Marques, MOM; Mondego, JMC	Comparative analysis indicates a simple protocol for DNA extraction of the aromatic plant *Lippia alba*	Analytical Biochemistry	01	Botanical study
Crotti, AEM; Pagotti, MC; Candido, ACBB; Marçal, MG; Vieira, TM; Groppo, M; Silva, MLA; Ferreira, DS; Esperandim, VR; Magalhaes, LG	Trypanocidal Activity of *Dysphania ambrosioides, Lippia alba,* and *Tetradenia riparia* Essential Oils against *Trypanosoma cruzi*	Chemistry & Biodiversity	03	Focus on other areas of study

Authors	Article Title	Journal	WoS-CC citations	Reason for exclusion
Nascimento, LSD; Lopes, JML; de Matos, EM; Souza, VC; Batista, DS; Santos, MD; Otoni, WC; Viccini, LF	Evaluation of reference genes for quantitative analysis of gene expression in *Lippia alba* under abiotic stress	Plant Cell Tissue And Organ Culture	0	Botanical study
Pascual, ME; Slowing, K; Carretero, ME; Villar, A	Antiulcerogenic activity of *Lippia alba* (Mill.) N. E. Brown (Verbenaceae)	Farmaco	33	Focus on other areas of study
Olivero-Verbel, J; González-Cervera, T; Güette-Fernandez, J; Jaramillo-Colorado, B; Stashenko, E	Chemical composition and antioxidant activity of essential oils isolated from Colombian plants	Brazilian Journal Of Pharmacognosy	39	Phytochemical study
Serra, BNV; Martins, ML; Pereira, SA; Chagas, EC; Chaves, FCM; Oliveira, MIB; Jeronimo, GT	Ovicidal effect of essential oils of *Lippia alba*, *Lippia sidoides* and *Lippia gracilis* on the acanthocephalan Neoechinorhynchus buttnerae (*Eoacanthocephala: Neoechinorhynchidae*)	Journal Of Fish Diseases	02	Focus on other areas of study
Teles, S; Pereira, JA; Santos, CHB; Menezes, RV; Malheiro, R; Lucchese, AM; Silva, F	Geographical origin and drying methodology may affect the essential oil of *Lippia alba* (Mill) NE Brown	Industrial Crops And Products	34	Botanical study
Costa, DCM; Vermelho, AB; Almeida, CA ; Dias, EPD; Cedrola, SML; Arrigoni-Blank, MD; Blank; Alviano, CS; Alviano, DS	Inhibitory effect of linalool-rich essential oil from *Lippia alba* on the peptidase and keratinase activities of dermatophytes	Journal Of Enzyme Inhibition And Medicinal Chemistry	15	Focus on other areas of study
Acero-Godoy, J; Guzmán-Hernández, T; Muñoz-Ruíz, C	Documentary review of essential oils obtained from *Lippia alba* (Verbenaceae) as microbial and antifungal therapeutic alternative	Tecnologia En Marcha	0	Focus on other areas of study
Jannuzzi, H; Mattos, JKA; Vieira, RF; Silva, DB; Bizzo, HR; Gracindo, LAM	Agronomic evaluation and identification of *Lippia alba* chemotypes from Distrito Federal, Brazil	Horticultura Brasileira	18	Focus on Agricultural Sciences
Veeck, APDL; Klein, B; Ruviaro, AR; Quatrin, A; Ferreira, LF; Daniel, AP; Piccolo, J; Oliveira, MS; Mallmann, CA; Heinzmann, BM; Emanuelli, T	Lipid stability of frozen common carp fillets treated with *Lippia alba* extract	Ciência Rural	05	Focus on other areas of study
Veras, HNH; Campos, AR; Rodrigues, FFG; Botelho, MA; Coutinho, HDM; Menezes, IRA; da Costa, JGM	Enhancement of the antibiotic activity of erythromycin by volatile compounds of *Lippia alba* (Mill.) NE Brown against *Staphylococcus aureus*	Pharmacognosy Magazine	09	Focus on other areas of study

Authors	Article Title	Journal	WoS-CC citations	Reason for exclusion
Rufino, ER; Siqueira, WJ; Marques, MOM; Colombo, CA; Azevedo, JA; Martins, ALM	Selection of new clones of linalool chemotype from genetic recombination in *Lippia alba*	Bragantia	02	Botanical study
Ramirez, JJQ; Rugeles, CIG; Stashenko, EE; Hernández, JCM; Galvis, MLD; Sanchez, LTG	In vivo protection against chagasic cardiomyopathy progression using trypanocidal fractions from *Lippia alba* (Verbenaceae) essential oils	Industrial Crops And Products	04	Focus on other areas of study
Muñoz-Acevedo, A; González, MC; Rodríguez, JD; De Moya, YS	New Chemovariety of *Lippia alba* From Colombia: Compositional Analysis of the Volatile Secondary Metabolites and Some in vitro Biological Activities of the Essential Oil From Plant Leaves	Natural Product Communications	03	Phytochemical study
Veloza, LA; Orozco, LM; Sepúlveda-Arias, JC	Use of Dimethyldioxirane in the Epoxidation of the Main Constituents of the Essential Oils Obtained from *Tagetes lucida*, *Cymbopogon citratus*, *Lippia alba* and *Eucalyptus citriodora*	Natural Product Communications	06	Botanical study
Glamoclija, J; Sokovic, M; Tesevic, V; Linde, GA; Colauto, NB	Chemical characterization of *Lippia alba* essential oil: an alternative to control green molds	Brazilian Journal Of Microbiology	24	Focus on other areas of study
Bonilla-Carvajal, K; Stashenko, EE; Moreno-Castellanos, N	Essential Oil of Carvone Chemotype *Lippia alba* (Verbenaceae) Regulates Lipid Mobilization and Adipogenesis in Adipocytes	Current Issues In Molecular Biology	07	Focus on other areas of study
García, LT; Leal, AF; Moreno, ÉM; Stashenko, EE; Arteaga, HJ	Differential anti-proliferative effect on K562 leukemia cells of *Lippia alba* (Verbenaceae) essential oils produced under diverse growing, collection and extraction conditions	Industrial Crops And Products	24	Focus on other areas of study
Hohlenwerger, JC; Baldisserotto, B; Couto, RD; Heinzmann, BM; da Silva, DT; Caron, BO; Schmidt, D; Copatti, CE	Essential oil of *Lippia alba* in the transport of Nile tilapia	Ciência Rural	15	Focus on other areas of study
Rugeles-Páez, NC; Quintero, WL; Stashenko, EE; García, LT	Citral-rich fractions of *Lippia alba* essential oils as immunoresponsive and anti-*Candida albicans* additives for collagen membranes in guided bone regeneration	Journal Of Oral Science	0	Focus on other areas of study
Jaramillo-Colorado, BE; Stashenko, EE; Winterhalter, P	Fractionation of four Colombian essential oils by countercurrent chromatography and evaluation of their antioxidant activity	Journal Of Essential Oil Research	04	*Lippia alba* is not the focus of the paper

Authors	Article Title	Journal	WoS-CC citations	Reason for exclusion
Lima, CB ; Boaventura, AC; Gomes, MM	Cuttings of *Lippia alba* with emphasis on time for seedling formation, substrates and plant growth regulators	Horticultura Brasileira	01	Plant physiology study
Espinel-Mesa, DX; Rugeles, CIG ; Hernández, JCM; Stashenko, EE; Villegas-Lanau, CA; Ramírez, JJQ; Sánchez, LTG	Immunomodulation and Antioxidant Activities as Possible Trypanocidal and Cardioprotective Mechanisms of Major Terpenes from *Lippia alba* Essential Oils in an Experimental Model of Chronic Chagas Disease	Antioxidants	05	Focus on other areas of study
de Almeida, MP; Silva, LAD; Zimmermann, LA; Bernardes, LSC; da Silva, MA; da Silva, GA; Amaral, JG; Gomes, AF; Leite, MF	Qualitative and quantitative chemical characterization of spray-dried extracts and the development of an innovative oral solid formulation from *Lippia alba*	Separation Science Plus	02	Biotechnology study
Machado, TF; Pereira, RDA; Batista, VCV	Seasonal variability of the antimicrobial activity of the essential oil of *Lippia alba*	Revista Ciência Agronômica	03	Phytochemical study
Batista, DS; de Castro, KM; da Silva, AR; Teixeira, ML; Sales, TA; Soares, LI; Cardoso, MD; Santos, MD; Viccini, LF; Otoni, WC	Light quality affects in vitro growth and essential oil profile in *Lippia alba* (Verbenaceae)	In Vitro Cellular & Developmental Biology-Plant	43	Plant physiology study
Hennebelle, T; Sahpaz, S; Dermont, C; Joseph, H; Bailleul, F	The essential oil of *Lippia alba*: Analysis of samples from French overseas departments and review of previous works	Chemistry & Biodiversity	41	Phytochemical study
Borges, AS; Bastos, CMS; Dantas, DM; Milfont, CGB ; Brito, GMH ; Pereira-de-Morais, L; Delmondes, GA; da Silva, RER; Kennedy-Feitosa, E; Maia, FPA; Lima, CMG; Bin Emran, T; Coutinho, HDM; Menezes, IRA; Kerntopf, MR; Caruso, G; Barbosa, R	Effect of *Lippia alba* (Mill.) N.E. Brown Essential Oil on the Human Umbilical Artery	Plants-Basel	06	Focus on other areas of study
Mercedes, CF; Cinthia, CA; Gabriela, CC; Nubia, MC	Infusions of *Moringa oleifera* (Moringa) combined with *Cymbopogon citratus* (Lemon grass) and *Lippia alba* (Mastranto)	Ciência Unemi	0	Phytochemical study
Veeck, APL; Klein, B; Ferreira, LF; Becker, AG; Heldwein, CG; Heinzmann, BM; Baldisserotto, B; Emanuelli, T	Lipid stability during the frozen storage of fillets from silver catfish exposed in vivo to the essential oil of *Lippia alba* (Mill.) NE Brown	Journal Of The Science Of Food And Agriculture	29	Focus on other areas of study

Authors	Article Title	Journal	WoS-CC citations	Reason for exclusion
Rabha, S; Devi, B; Goswami, M; Das, E; Sarma, N; Devi, A	Ecologically Safe Removal of Lead Ions and Basic Fuchsin Dye from an Aqueous Solution Using *Lippia alba* Leaves	Chemistryselect	01	Study in ecology
Mishra, RK; Chaudhary, S; Pandey, R; Gupta, S; Mallavarapu, GR; Kumar, S	Analysis of Linalool Content in the Inflorescence (Flower) Essential Oil and Leaf Oil of *Lippia alba* Cultivar 'Kavach'	Journal Of Essential Oil Research	05	Phytochemical study
dos Santos, LGA; dos Reis, RB; Souza, ASQ; Canuto, KM; de Brito, ES; Castro, KNC. Pereira, AML; Diniz, FM	Chemical composition and biological activities of the essential oils from *Lippia alba* and *Lippia origanoides*	Anais Da Academia Brasileira De Ciências	01	Study in aquaculture
Silva, PTD; de Souza, LM; de Morais, MB; de Moraes, MM; da Camara, CAG; Ulisses, C	Effect of biotic elicitors on the physiology, redox system, and secondary metabolite composition of *Lippia alba* (Mill.) cultivated *in vitro*	South African Journal Of Botany	04	Plant physiology study
Gimenes, L; Silva, JCRL; Facanali, R; Hantao, LW; Siqueira, WJ; Marques, MOM	Essential Oils of New *Lippia alba* Genotypes Analyzed by Flow-Modulated Comprehensive Two-Dimensional Gas Chromatography (GCxGC) and Chemometric Analysis	Molecules	07	Phytochemical study
Maynard, LG; Santos, KC; Cunha, PS; Barreto, AS; Peixoto, MG; Arrigoni-Blank, F; Blank, AF; Alves, PB; Bonjardin, LR; Santos, MRV	Chemical composition and vasorelaxant effect induced by the essential oil of *Lippia alba* (Mill.) NE Brown. (Verbenaceae) in rat mesenteric artery	Indian Journal Of Pharmacology	15	Focus on other areas of study
Forero-Peñuela, LY; Biasi, LA; Bizzo, HR; de Souza, MS; Deschamps, C	Potential of *Lippia alba* (Mill.) NE Br. ex Britt. & P. Wilson, as available source of linalool in southern Brazil	Journal Of Essential Oil Research	01	Phytochemical study
da Silva, RER; de Morais, LP; Silva, AA; Bastos, CMS; Pereira-Gonçalves, A; Kerntopf, MR; Menezes, IRA; Leal-Cardoso, JH; Barbosa, R	Vasorelaxant effect of the *Lippia alba* essential oil and its major constituent, citral, on the contractility of isolated rat aorta	Biomedicine & Pharmacotherapy	22	Focus on other areas of study
Braga, MEM; Ehlert, PAD; Ming, LC; Meireles, MAA	Supercritical fluid extraction from *Lippia alba*: global yields, kinetic data, and extract chemical composition	Journal Of Supercritical Fluids	51	Phytochemical study
Aline, DB; Viccini, LF; Recco-Pimentel, SM	Meiotic analysis of two putative polyploid species of Verbenaceae from Brazil	Caryologia	02	Botanical study

Authors	Article Title	Journal	WoS-CC citations	Reason for exclusion
Gomes, AF; Almeida, MP; Ruela, ALM; Amaral, JG; David, JM; Leite, MF	Development and evaluation of physical and release properties of a tablet formulation containing dry hydroethanolic extract from *Lippia alba* leaves	Journal Of Herbal Medicine	04	Biotechnology study
Mesa-Arango, AC; Montiel-Ramos, J; Zapata, B; Durán, C; Betancur-Galvis, L; Stashenko, E	Citral and carvone chemotypes from the essential oils of Colombian *Lippia alba* (Mill.) NE Brown: composition, cytotoxicity and antifungal activity	Memorias Do Instituto Oswaldo Cruz	92	Focus on other areas of study
de Castro, KM; Batista, DS; Silva, TD; Fortini, EA; Felipe, SHS; Fernandes, AM; Sousa, RMD; Nascimento, LSD; Campos, VR; Viccini, LF; Michael, R; Otoni, WC	Salinity modulates growth, morphology, and essential oil profile in *Lippia alba* L. (Verbenaceae) grown in vitro	Plant Cell Tissue And Organ Culture	06	Plant physiology study
Pandey, AK; Sonker, N; Singh, P	Efficacy of Some Essential Oils Against *Aspergillus flavus* with Special Reference to *Lippia alba* Oil an Inhibitor of Fungal Proliferation and Aflatoxin B1 Production in Green Gram Seeds during Storage	Journal Of Food Scienc	33	Focus on Agricultural Sciences
Pereira-de-Morais, L; Silva, AD; da Silva, RER; da Costa, RHS; Monteiro, AB; Barbosa, CRD; Amorim, TD; de Menezes, IRA; Kerntopf, MR; Barbosa, R	Tocolytic activity of the *Lippia alba* essential oil and its major constituents, citral and limonene, on the isolated uterus of rats	Chemico-Biological Interactions	16	Focus on other areas of study
de Castro, KM; Batista, DS; Fortini, EA; Silva, TD; Felipe, SHS; Fernandes, AM; Sousa, RMD; Nascimento, LSD; Campos, VR; Grazul, RM; Viccini, LF; Otoni, WC	Photoperiod modulates growth, morphoanatomy, and linalool content in *Lippia alba* L. (Verbenaceae) cultured in vitro	Plant Cell Tissue And Organ Culture	14	Plant physiology study
Caballero-Gallardo, K; Fuentes-Lopez, K; Stashenko, EE; Olivero-Verbel, J	Chemical Composition, Repellent Action, and Toxicity of Essential Oils from *Lippia origanoide, Lippia. alba* Chemotypes, and *Pogostemon cablin* on Adults of *Ulomoides dermestoides* (Coleoptera: Tenebrionidae)	Insects	05	Focus on other areas of study
Lopes, JML; de Matos, EM; Zorzatto, C; Azevedo, ALS; Machado, MA; Chester, M; Viccini, LF	Development of microsatellite markers for *Lippia alba* and related *Lippia* species	Molecular Biology Reports	02	Botanical study

Authors	Article Title	Journal	WoS-CC citations	Reason for exclusion
Brandao, AD; Viccini, LF; Salimena, FRG; Vanzela, ALL; Recco-Pimentel, SM.	Cytogenetic characterization of *Lippia alba* and *Lantana camara* (Verbenaceae) from Brazil	Journal Of Plant Research	14	Botanical study
Nascimento, HD; Crispim, BD; Francisco, LFV; Merey, FM; Kummrow, F; Viana, LF; Inoue, LAKA; Barufatti, A	Genotoxicity evaluation of three anesthetics commonly employed in aquaculture using *Oreochromis niloticus* and *Astyanax lacustris*	Aquaculture Reports	06	Study in aquaculture
Yamamoto, PY; Colombo, CA; Azevedo, JA; Lourencao, AL; Marques, MOM; Morais, GDD; Chiorato, AF; Martins, ALM; Siqueira, WJ	Performance of ginger grass (*Lippia alba*) for traits related to the production of essential oil	Scientia Agricola	20	Plant physiology study
Rao, GP; Singh, M; Singh, P; Singh, SP; Catalan, C; Kapoor, IPS; Singh, OP; Singh, G	Studies on chemical constituents and antifungal activity of leaf essential oil of *Lippia alba* (Mill)	Indian Journal Of Chemical Technology	15	Focus on Agricultural Sciences
Lopes, JML; de Carvalho, HH; Zorzatto, C; Azevedo, ALS; Machado, MA; Salimenas, FRG; Grazul, RM; Gitzendanner, MA; Soltis, DE; Soltis, PS; Viccini, LF	Genetic relationships and polyploid origins in the *Lippia alba* complex	American Journal Of Botany	09	Botanical study
Lopes, JML; Nascimento, LSD; Souza, VC; de Matos, EM; Fortini, EA; Grazul, RM; Santos, MO; Soltis, DE; Soltis, PS; Otoni, WC; Viccini, LF	Water stress modulates terpene biosynthesis and morphophysiology at different ploidal levels in *Lippia alba* (Mill.) N. E. Brown (Verbenaceae)	Protoplasma	0	Plant physiology study
Stashenko, EE; Jaramillo, BE; Martínez, JR	Comparison of different extraction methods for the analysis of volatile secondary metabolites of *Lippia alba* (Mill.) NE Brown, grown in Colombia, and evaluation of its in vitro antioxidant activity	Journal Of Chromatography A	200	Phytochemical study
Montero-Villegas, S; Polo, M; Galle, M; Rodenak-Kladniew, B; Castro, M; Ves-Losada, A; Crespo, R; de Bravo, MG	Inhibition of Mevalonate Pathway and Synthesis of the Storage Lipids in Human Liver-Derived and Non-liver Cell Lines by *Lippia alba* Essential Oils	Lipids	10	Focus on other areas of study

**Authors**	**Article Title**	**Journal**	**WoS-CC citations**	**Reason for exclusion**
de Castro, KM; Batista, DS; Silva, TD; Fortini, EA; Felipe, SHS; Fernandes, AM; Sousa, RMD; Nascimento, LSD; Campos, VR; ; Grazul, RM; Viccini, LF; Otoni, WC	Water deficit modulates growth, morphology, and the essential oil profile in *Lippia alba* L. (Verbenaceae) grown in vitro	Plant Cell Tissue And Organ Culture	13	Plant physiology study
Noguma, MA; Diaz, G; Sakumo, L; Tagami, PM	Antibacterial activity of *Lippa alba* (Lemon herb)	Latin American Journal Of Pharmacy	03	Focus on other areas of study
Shukla, R; Kumar, A; Singh, P; Dubey, NK	Efficacy of *Lippia alba (*Mill.) NE Brown essential oil and its monoterpene aldehyde constituents against fungi isolated from some edible legume seeds and aflatoxin B_1 production	International Journal Of Food Microbiology	100	Focus on Agricultural Sciences
Jezler, CN; de Oliveira, ARMF; Batista, RS; Oliveira, RA; Silva, DD; Costa, LCD	*Lippia alba* morphotypes cidreira and melissa exhibit significant differences in leaf characteristics and essential oil profile	Revista Brasileira De Farmacognosia-Brazilian Journal Of Pharmacognosy	14	Phytochemical study
Shukla, R; Singh, P; Prakash, B; Kumar, A; Mishra, PK; Dubey, NK	Efficacy of essential oils of *Lippia alba* (Mill.) NE Brown and Callistemon lanceolatus (Sm.) Sweet and their major constituents on mortality, oviposition and feeding behaviour of pulse beetle, *Callosobruchus chinensis L.*	Journal Of The Science Of Food And Agriculture	46	Focus on other areas of study
Couto, HGSD; Barbosa, AAT; Nizio, DAD; Nogueira, PCD; Arrigoni-Blank, MD; Pinto, JAO; Alves, MF; Pinto, VD; Blank, AF	Antibacterial activity of *Lippia alba*, *Myrcia lundiana* and *Ocimum basilicum* essential oils against six food-spoiling pathogenic microorganisms	Boletin Latinoamericano Y Del Caribe De Plantas Medicinales Y Aromaticas	0	Focus on other areas of study
Duarte, ME; Chetverikov, PE; Silva, ES; Navia, D	Three new species of eriophyoid mites (*Acariformes, Eriophyoidea*) from *Lippia alba* (Verbenaceae) from Brazil, and remarks on the thorn-like spermathecal process	Systematic And Applied Acarology	34	Focus on other areas of study
Day, MD; McAndrew, TD	The biology and host range of *Falconia intermedia* (Hemiptera: Miridae), a potential biological control agent for *Lantana camara* (Verbenaceae) in Australia	Biocontrol Science And Technology	17	Focus on other areas of study
Kishore, N; Mishra, AK	Effect of essential oils on sclerotial germination of *Rhizoctonia-solani*	National Academy Science Letters-India	04	Focus on Agricultural Sciences
Jaramillo-Colorado, B; Olivero-Verbel, J; Stashenko, EE; Wagner-Döbler, I; Kunze, B	Anti-quorum sensing activity of essential oils from Colombian plants	Natural Product Research	40	Focus on other areas of study

Authors	Article Title	Journal	WoS-CC citations	Reason for exclusion
Quintero, WL; Moreno, EM; Pinto, SML; Sanabria, SM; Stashenko, E; García, LT	Immunomodulatory, trypanocide, and antioxidant properties of essential oil fractions of *Lippia alba* (Verbenaceae)	BMC Complementary Medicine And Therapies	08	Focus on Lippia alba secondary metabolites
Deka, B; Pandey, AK; Babu, A; Baruah, C; Sarkar, S	Acaricidal and ovicidal properties of *Lippia alba* essential oil and its chemical constituents against red spider mite, *Oligonychus coffeae* Nietner (Acari: Tetranychidae) infesting tea crops	Archives Of Phytopathology And Plant Protection	01	Bioplaguicide study
Tavares-Dias, M; Neves, LR; Alves, CMG; Nogueira, JN; Neves, FB; Pinto, AVP; Carvalho, JCT; Ferreira, IM	In vitro anthelminthic activity of *Lippia alba* essential oil combined with silk fibroin against monogeneans of *Colossoma macropomum* (Serrasalmidae)	Aquaculture Research	01	Study in aquaculture
Joshi, A; Prakash, O; Pant, AK; Kumar, R ; Negi, MS	Chemical Analysis and Antioxidant Activity of Essential Oils of Two Morphotypes of *Lippia alba* (Mill.) NE Br. ex Britton & P. Wilson (Verbenaceae)	Journal Of Essential Oil Bearing Plants	08	Phytochemical study
Mota, APP; Dantas, JCP; Frota, CC	Antimicrobial activity of essential oils from *Lippia alba, Lippia sidoides, Cymbopogon citrates, Plectranthus amboinicus*, and *Cinnamomum zeylanicum* against *Mycobacterium tuberculosis*	Ciência Rural	11	Focus on other areas of study
Juliao, SA; Ribeiro, CD; Lopes, JML; de Matos, EM; Reis, AC; Peixoto, PHP; Machado, MA; Azevedo, ALS; Grazul, RM; de Campos, JMS; Viccini, LF	Induction of Synthetic Polyploids and Assessment of Genomic Stability in *Lippia alba*	Frontiers In Plant Science	28	Botanical study
Caballero-Gallardo, K; Olivero-Verbel, J; Stashenko, EE	Repellent Activity of Essential Oils and Some of Their Individual Constituents against *Tribolium castaneum* Herbst	Journal Of Agricultural And Food Chemistry	109	Focus on other areas of study
Sutili, FJ; Cunha, MA; Ziech, RE; Krewer, CC; Zeppenfeld, CC; Heldwein, CG; Gressler, LT; Heinzmann, BM; Vargas, AC; Baldisserotto, B	*Lippia alba* essential oil promotes survival of silver catfish (*Rhamdia quelen*) infected with Aeromonas sp.	Anais Da Academia Brasileira De Ciencias	13	Study in aquaculture
Sepúlveda-Arias, JC; Veloza, LA; Escobar, LM; Orozco, LM; Lopera, IA	Anti-inflammatory effects of the main constituents and epoxides derived from the essential oils obtained from *Tagetes lucida, Cymbopogon citratus, Lippia alba* and *Eucalyptus citriodora*	Journal Of Essential Oil Research	21	*Lippia alba* is not the focus of the paper

Authors	Article Title	Journal	WoS-CC citations	Reason for exclusion
Lopes, JML; Campos, VR; Reis, AC; de Matos, EM; Azevedo, ALS; Machado, MA; Grazul, RM; Viccini, LF	Aneuploids and its increment on diversity of *Lippia alba* polyploid complex: genetic aspects and origin	Molecular Biology Reports	02	Botanical study
Veeck, APD; Daniel, AP; Klein, B; Quatrin, A; Rezer, APD ; Milani, LG; Zeppenfeld, CC; da Cunha, MA; Heldwein, CG; Heinzmann, BM; Parodi, TV; Baldisserotto, B; Emanuelli, T	Chemical, microbiological, and sensory parameters during the refrigerated storage of silver catfish (*Rhamdia quelen*) exposed in vivo to the essential oil of *Lippia alba*	Journal Of Food Science And Technology-Mysore	09	Study in aquaculture
Tagami, OK; Gasparin, MDG; Schwan-Estrada, KRF; Cruz, MED; Itako, AT; Tolentino, JB; de Moraes, LM; Stangarlin, JR	Fungitoxicty of *Bidens pilosa, Thymus vulgaris, Lippia alba* and *Rosmarinus officinalis* in the in vitro development of phytophatogens fungi	Semina-Ciencias Agrarias	10	Focus on Agricultural Sciences
Costa, M; Distasi, LC; Kirizawa, M); Mendacolli, SLI); Gomes, C); Trolin, G	Screening in mice of some medicinal-plants used for analgesic purposes in the state of Sao-Paulo .2.	Journal Of Ethnopharmacology	24	*Lippia alba* is not the focus of the paper
Oliveira, TAS; Santiago, MB; Santos, VHP; Silva, EO; Martins, CHG; Crotti, AEM	Antibacterial Activity of Essential Oils against Oral Pathogens	Chemistry & Biodiversity	06	Article not found
do Vale, TG; Furtado, EC; Santos JG; Viana, GSB	Central effects of citral, myrcene and limonene, constituents of essential oil chemotypes from *Lippia alba* (Mill.) NE Brown	Phytomedicine	142	Focus on Lippia alba secondary metabolites
Ehlert, PAD; Chaves, FCM; Ming, LC; da Silva, MAS	Effect of substrata on the development of stem cuttings of *Lippia alba* (Mill.) NEBR. -: Limonene-carvone chemotype	International Society Horticultural Science	01	Plant physiology study
Day, MD; Riding, N; Chamberlain, A	Biology and host range of *Ophiomyia camarae* Spencer (Diptera: Agromyzidae), a potential biocontrol agent for *Lantana spp.* (Verbenaceae) in Australia	Biocontrol Science And Technology	06	Focus on other areas of study
Blanco, MA; Colareda, GA; van Baren, C; Bandoni, AL; Ringuelet, J; Consolini, AE	Antispasmodic effects and composition of the essential oils from two South American chemotypes of *Lippia alba*	Journal Of Ethnopharmacology	35	Focus on other areas of study
de Souza, CM; Zorzatto, C; Quinhones, CGS; Lopes, JML; de Carvalho, HH; Araújo, WL; Viccini, LF	Deciphering ploidal levels of *Lippia alba* by using proteomics	Plant Physiology And Biochemistry	02	Botanical study

Authors	Article Title	Journal	WoS-CC citations	Reason for exclusion
Olivero-Verbel, J; Barreto-Maya, A; Bertel-Sevilla, A; Stashenko, EE	Composition, anti-quorum sensing and antimicrobial activity of essential oils from *Lippia alba*	Brazilian Journal Of Microbiology	16	Focus on other areas of study
de Souza, VC; Aragao, MM; Tavares, LS; Capriles, PVSZ ; Viccini, LF; Santos, MO	De novo leaf transcriptome of a triploid linalool chemotype of *Lippia alba* (Mill.) NEBr. ex Britton & P. Wilson	Brazilian Journal Of Botany	02	Botanical study
Azambuja, CR; Mattiazzi, J; Riffel, APK; Finamor, IA; Garcia, LD; Heldwein, CG; Heinzmann, BM; Baldisserotto, B; Pavanato, MA; Llesuy, SF	Effect of the essential oil of *Lippia alba* on oxidative stress parameters in silver catfish (*Rhamdia quelen*) subjected to transport	Aquac8ulture	82	Study in aquaculture
Majolo, C; da Rocha, SIB; Chagas, EC; Chaves, FCM; Bizzo, HR	Chemical composition of *Lippia spp.* essential oil and antimicrobial activity against *Aeromonas hydrophila*	Aquaculture Research	31	Study in aquaculture
Reis, AC; Sousa, SM; Viccini, LF	High frequency of cytomixis observed at zygotene in tetraploid *Lippia alba*	Plant Systematics And Evolution	24	Botanical study
Majolo, C; Pilarski, F; Chaves, FCM; Bizzo, HR; Chagas, EC	Antimicrobial activity of some essential oils against *Streptococcus agalactiae*, an important pathogen for fish farming in Brazil	Journal Of Essential Oil Research	12	Study in aquaculture
Penido, AB; de Morais, SM; Ribeiro, AB; Silva, AZ	Ethnobotanical study of medicinal plants in Imperatriz, State of Maranhao, Northeastern Brazil	Acta Amazônica	15	Ethnobotanical study
Alves, AC; Jesus, FN; Alves, PB; Santos, HV; Souza, GS; Santos, AR	Biomass production and essential oil of lemon balm cultivated under colored screens and nitrogen	Horticultura Brasileira	06	Botanical study
Vélez-Martínez, GA; Duque-Zapata, JD; Reyes-Ardila, WL; Flórez, JEM; Gallo, SAD; Ariza, LAD; López-Alvarez, D	Comparative Analysis of Bacteria, Fungi, and *Arbuscular Mycorrhizal* Fungi in Medicinal Plants *Lippia alba* and *Petiveria alliacea* in Colombia	Diversity-Basel	0	Plant physiology study
Bianchini, AE ; Descovi, SN; Heinzmann, BM; Baldisserotto, B	Linalool induces relaxation of the mantle of golden apple snail (*Pomacea canaliculata*)	Anais Da Academia Brasileira De Ciencias	0	*Lippia alba* is not the focus of the paper
de Lima, ABS; Queiroga, IMBN; Silva, GMD; da Costa, JS; Guedes, JPD; Dantas, CD; Cavalcanti, MT	Characterization and application of *Lippia alba* (Mill) and *Cymbopogon citratus* DC Stapf. essential oils as natural sanitizers in coriander	Food Science And Technology	06	Focus on Agricultural Sciences

Authors	Article Title	Journal	WoS-CC citations	Reason for exclusion
Tomazoni, EZ; Pansera, MR; Pauletti, GF; Moura, S; Ribeiro, RTS; Schwambach, J	In vitro antifungal activity of four chemotypes of *Lippia alba* (Verbenaceae) essential oils against *Alternaria solani* (Pleosporeaceae) isolates	Anais Da Academia Brasileira De Ciências	22	Focus on Agricultural Sciences
Silva, LL; Balconi, LS; Gressler, LT; Garlet, QI; Sutili, FJ ; Vargas, APC; Baldisserotto, B; Morel, AF; Heinzmann, BM	S-(+)- and R-(-)-linalool: a comparison of the in vitro anti-Aeromonas hydrophila activity and anesthetic properties in fish	Anais Da Academia Brasileira De Ciências	22	Focus on Lippia alba secondary metabolites
Veit, JC ; Piccolo, J; Scherer, AF ; Machado, IS; Maurer, LH; Conte, L; Baldisserotto, B; Koakoski, G; Menezes, CC ; Loro, VL; Barcellos, LJG; Heinzmann, BM; Poletto, G; Menezes, CR; Emanuelli, T	Preslaughter Anesthesia with *Lippia alba* Essential Oil Delays the Spoilage of Chilled *Rhamdia quelen*	Journal Of Aquatic Food Product Technology	06	Study in aquaculture
Mahanta, S; Sarma, R; Khanikor, B	The essential oil of *Lippia alba* Mill (Lamiales:Verbenaceae) as mosquitocidal and repellent agent against *Culex quinquefasciatus* Say (Diptera: Culicidae) and *Aedes aegypti* Linn (Diptera: Culicidae)	Journal Of Basic And Applied Zoology	09	Focus on other areas of study
Lima, AD; De Carvalho, JF; Peixoto, MG; Blank, AF; Borges, LMF; Costa, LM	Assessment of the repellent effect of *Lippia alba* essential oil and major monoterpenes on the cattle tick *Rhipicephalus microplus*	Medical And Veterinary Entomology	31	Focus on other areas of study
de Souza, RC; da Costa, MM; Baldisserotto, B; Heinzmann, BM; Schmidt, D; Caron, BO; Copatti, CE	Antimicrobial and synergistic activity of essential oils of *Aloysia triphylla* and *Lippia alba* against *Aeromonas spp.*	Microbial Pathogenesis	38	Focus on other areas of study
Geromini, KVN; Roratto, FB; Ferreira, FG; Camilotti, J; Vidigal, TMA; Valle, JS; Colauto, NB; Linde, GA	Fungicidal effect of *Lippia alba* essential oil on a white-rot fungus	Maderas-Ciencia Y Tecnologia	4	Focus on other areas of study
Juiz, PJL; Lucchese, AM; Gambari, R; Piva, R; Penolazzi, L; Di Ciano, M; Uetanabaro, APT; Silva, F; Avila-Campos, MJ	Essential oils and isolated compounds from *Lippia alba* leaves and flowers: Antimicrobial activity and osteoclast apoptosis	International Journal Of Molecular Medicine	10	Focus on other areas of study

Authors	Article Title	Journal	WoS-CC citations	Reason for exclusion
de Sá, JCFD; Nizio, DAD; de Oliveira, AMS; Alves, MF; de Oliveira, RC; Luz, JMQ; Nogueira, PCD; Arrigoni-Blank, MD; Blank, AF	Geographic location and seasonality affect the chemical composition of essential oils of *Lippia alba* accessions	Industrial Crops And Products	04	Phytochemical study
Freitas, JCE; de Resende, CF; Pacheco, VS; Grazul, RM; Morais, LE; Passos, LP; Peixoto, PHP	Does the water regime differentially modulate the responses to water stress in *Lippia alba* (Verbenaceae) genotypes with different ploidy levels?	Industrial Crops And Products	06	Plant physiology study
Souza, CD; Baldissera, MD; Bianchini, AE; da Silva, EG; Mourao, RHV; da Silva, LVF; Schmidt, D; Heinzmann, BM; Baldisserotto, B	Citral and linalool chemotypes of *Lippia alba* essential oil as anesthetics for fish: a detailed physiological analysis of side effects during anesthetic recovery in silver catfish (*Rhamdia quelen*)	Fish Physiology And Biochemistry	59	Study in aquaculture
Kumari, KMU; Chanotiya, CS; Yadav, NP; Tandon, S; Luqman, S	Essential oil of *Lippia alba* impedes the growth of *Propionibacterium acnes* by modulating membrane potential and ROS level	Journal Of Herbal Medicine	0	Focus on other areas of study
Soares, BV; Neves, LR; Oliveira, MSB; Chaves, FCM; Dias, MKR; Chagas, EC; Tavares-Dias, M	Antiparasitic activity of the essential oil of *Lippia alba* on ectoparasites of *Colossoma macropomum* (tambaqui) and its physiological and histopathological effects	Aquaculture	63	Study in aquaculture
Rocha, GO; Scabini, C; Oliveira, JFD; Oliveira, DN; Mota, LC; Neves, BNA; dos Santos, AM; de Souza, FM; de Araujo, US; Moreira, BO; de Almeida, MP; Amaral, JG; David, JM; Gomes, AF	Seasonal and Circadian Variation of Non-Volatile Metabolites in *Lippia alba* Leaves	Chemistry & Biodiversity	0	Focus on other areas of study
Souza, RC; de Souza, EM; da Costa, MM; Melo, JFB; Baldisserotto, B; Copatti, CE	Dietary addition of the essential oil from *Lippia alba* to Nile tilapia and its effect after inoculation with *Aeromonas spp*	Aquaculture Nutrition	26	Study in aquaculture
Reis, AC; Chester, M; de Sousa, SM; Campos, VR; Nascimento, LSD; Júnior, SP; Franco, AL; Viccini, LF	Chromosomal view of *Lippia alba*, a tropical polyploid complex under genome stabilization process	Protoplasma	06	Botanical study
Leitao, GG; Pinto, SC; de Oliveira, DR; Timoteo, P; Guimaraes, MG; Cordova, WHP; Leitao, SG	Gradient x Isocratic Elution CCC on the Isolation of Verbascoside and Other Phenylethanoids: Influence of the Complexity of the Matrix	Planta Médica	06	Phytochemical study

Authors	Article Title	Journal	WoS-CC citations	Reason for exclusion
Venâncio, DDA; Viccini, LF; Luizi-Ponzo, AP; Prezoto, F	Flower-Visiting Insects and Phenology of *Lippia alba* (Lamiales: Verbenaceae): Floral Color Changes and Environmental Conditions as Cues for Pollinators	Environmental Entomology	05	Focus on other areas of study
Peixoto, MG; de Oliveira, ACL; Blank, AF; Silva, JHS; Luz, JMQ; Arrigoni-Blank, MD	In vitro conservation and leaf anatomy of different chemotypes of *Lippia alba* (Mill.) N. E. BR	Bioscience Journal	04	Botanical study
Cagol, L; Baldisserotto, B; Becker, AG; Souza, CD; Heinzmann, BM; Caron, BO; Leone, FA; dos Santos, LD; Ballester, ELC	Essential oil of Lippia alba in the diet of *Macrobrachium rosenbergii*: Effects on antioxidant enzymes and growth parameters	Aquaculture Research	08	study in aquaculture
Moreno, ÉM; Leal, SM; Stashenko, EE; García, LT	Induction of programmed cell death in *Trypanosoma cruzi* by *Lippia alba* essentia oils and their major and synergistic terpenes (citral, limonene and caryophyllene oxide)	BMC Complementary And Alternative Medicine	35	Focus on other areas of study
Majolo, C; Bizzo, HR; Brandao, FR; da Silva, AMS; Chagas, EC; Chaves, FCM; Muniz, AW	Chemical composition of *Lippia Linn.* (Verbenaceae) essential oils and their antibacterial potential *against Aeromonas spp.* isolates from *Colossoma macropomum*	Journal Of Essential Oil Research	01	Study in aquaculture
Costa, PS; Oliveira, SS; de Souza, EB ; de Brito, EHS; Cavalcante, CSD; de Morais, SM; Leal, ALAB; Barreto, HM ; Teixeira, AMR; Nogueira, CES; Fontenelle, ROD; dos Santos, HS	Antifungal Activity and Synergistic Effect of Essential oil from *Lippia alba* Against *Trichophyton rubrum* and *Candida spp.*	Revista virtual de química	06	Focus on other areas of study
da Silva, HNP; Sousa, EMD; Maia, JLD; Pinheiro, MTL; Lameirao, SVDC; Mourao, RHV; Maia, JGS; Baldisserotto, B; da Silva, LVF	*Lippia alba* (Verbenaceae) hydrolate as sedative of tambaqui (*Colossoma macropomum*) juveniles in simulated transport conditions	Aquaculture Research	11	Study in aquaculture
Saulo, S; Reis, AC; Silva, PS; Viccini, LF	An increment in the Duranta repens *L.* (Verbenaceae) knowledge: DNA content, karyology, meiosis and palynology	Caryologia	0	Botanical study

Authors	Article Title	Journal	WoS-CC citations	Reason for exclusion
Torre, R; de Medeiros, EADP; Pereira, CDB; Menezes, ACR; Fontes, IS; Pereira, LVR; Paiva, DHF; dos Santos, AM; Damasceno, PC Jr; de Souza, MAA	Protection of cowpea seeds and toxicity gainst cowpea weevils by the essential oils from *Lippia alba* (verbenaceae) and *Schinus terebinthifolius* (anacardiaceae)	Crop Protection	0	Focus on Agricultural Sciences
de Almeida, ET; Correia, ES; do Nascimento, CH; Bezerra, RD; Cahu, T; Chung, S; Copatti, CE; Ribeiro, K	*Macrobrachium rosenbergii* fed with essential oil from *Lippia alba* in the diet in low and high stocking density	Aquaculture Research	03	Study in aquaculture
Rossato, M; dos Santos, ACA; Serafini, LA; Agostini, F; Pansera, MR; Wasum, R; Barbieri, RL	Evaluation of the essential oil of *Aloysia sellowii* (Briquet) moldenke (Verbenaceae) from south Brazil.	Quimica Nova	0	*Lippia alba* is not the focus of the paper
Antonio-Gutiérrez, O; Alvízar-Martínez, JA; Solano, R; Vásquez-López, A; Hernández-Valladolid, SL; Lustre-Sánchez, H; Flores-Moctezuma, HE; de Luna-Santillana, ED; Lagunez-Rivera, L	Microwave-Assisted Hydrodistillation of Essential Oil from *Plectranthus amboinicus*: Evaluation of Its Antifungal Effect and Chemical Composition	Life-Basel	01	Focus on Agricultural Sciences
Andrighetti-Fröhner, CR; Sincero, TCM; da Silva, AC; Savi, LA; Gaido, CM; Bettega, JMR ; Mancini, M; de Almeida, MTR; Barbosa, RA; Farias, MR; Barardi, CRM; Simoes, CMO	Antiviral evaluation of plants from Brazilian Atlantic Tropical Forest	Fitoterapia	55	Focus on other areas of study
Gomes, DS; Negrao-Corrêa, DA; Miranda, GS; Rodrigues, JGM; Guedes, TJFL; de Lucca, W; de Sa, JCF; Nizio, DAD; Blank, AF; Feitosa, VLC; Dolabella, SS	*Lippia alb*a and *Lippia gracilis* essential oils affect the viability and oviposition of *Schistosoma mansoni*	Acta Tropica	06	Focus on other areas of study

Authors	Article Title	Journal	WoS-CC citations	Reason for exclusion
Marasca, S; Battisti, EK; Durigon, EG; Peixoto, NC; Uczay, J; Baldisserotto, B; Schmidt, D; Loro, VL; Leitemperger, JW; Salvador, MJ; Lazzari, R	Growth, hematology, metabolism, and oxidative parameters of silver catfish (*Rhamdia quelen*) fed diets containing *Lippia alba* leaf	Aquaculture	02	Study in aquaculture
Batista, DS; de Castro, KM; Ribeiro, DM; Caixeta, ET; Santos, MD; Viccini, LF; Otoni, WC	Ethylene Responses and ACC *oxidase* Gene Expression in *Lippia alba* (Verbenaceae) Chemotypes with Varying Ploidy Levels	In Vitro Cellular & Developmental Biology-Plant	03	Botanical study
Toni, C; Becker, AG; Simoes, LN; Pinheiro, CG; Silva, LD; Heinzmann, BM; Caron, BO ; Baldisserotto, B	Fish anesthesia: effects of the essential oils of *Hesperozygis ringens* and *Lippia alba* on the biochemistry and physiology of silver catfish (*Rhamdia quelen*)	Fish Physiology And Biochemistry	74	Study in aquaculture
Silva-Santos, L; Neto, LP; Corte-Real, N; Sperandio, MVL; Camara, CAG; Moraes, MM; Ulisses, C	Elicitation with Methyl Jasmonate and Salicylic Acid Increase Essential Oil Production and Modulate Physiological Parameters in *Lippia alba* (Mill) NE Brown (Verbenaceae)	Journal Of Plant Growth Regulation	01	Plant physiology study
Leite, PM; Miranda, APN; Amorim, JM; Santos, LB; Duarte, RCF; Maltarollo, VG; Viccini, LF; Faraco, AAG; Carvalho, MD; Castilho, RO	Correlation of chemical composition and anticoagulant activity in different accessions of Brazilian *Lippia alba* (Verbenaceae)	Journal Of Herbal Medicine	02	Focus on other areas of study
Machado, TF; Nogueira, NAP; Pereira, RDA; de Sousa, CT; Batista, VCV	The antimicrobial efficacy of *Lippia alba* essential oil and its interaction with food ingredients	Brazilian Journal of Microbiology	19	
Lima, TD; Baptista, NMD; De Oliveira, APS; Da Silva, PA; De Gusmao, NB; Correia, MTD; Napoleao, TH; Da Silva, MV; Paiva, PMG	Insecticidal activity of a chemotype VI essential oil from *Lippia alba* leaves collected at Caatinga and the major compound (1,8-cineole) against Nasutitermes corniger and *Sitophilus zeamais*	Pesticide Biochemistry And Physiology	15	Focus on Agricultural Sciences
Castro, MA; Girotti, JR; Dumrauf, B; Rodenak-Kladniew, B; Zaro, MJ; Otero, CM; Montero-Villegas, S; Bravo, MGD; Vina, SZ; Crespo, R	In vitro evaluation of antiatherogenic potential of *Origanum x paniculatum*, *Lippia alba, Clinopodium nepeta*, and *Eucalyptus globulus essential* oils	Journal of Herbal Medicine	0	Focus on other areas of study
Guerrero, MF; Puebla, P; Carrón, R; Martín, ML ; Arteaga, L ; San Román, L	Assessment of the antihypertensive and vasodilator effects of ethanolic extracts of some Colombian medicinal plants	Journal of Ethnopharmacology	56	Focus on other areas of study

Authors	Article Title	Journal	WoS-CC citations	Reason for exclusion
Saroj, A; Chanotiya, CS; Maurya, R ; Pragadheesh, VS; Yadav, A; Samad, A.	Antifungal action of *Lippia alba* essential oil in *Rhizoctonia solani* disease management	Sn Applied Sciences	05	Focus on Agricultural Sciences
Barbosa, MLF; Ribeiro, WLC; de Araújo, JV; Pereira, RDA; André, WPP; Melo, ACFL; Castelo-Branco, DDCM; de Morais, SM; de Oliveira, LMB; Bevilaqua, CML	In vitro anthelmintic activity of *Lippia alba* essential oil chemotypes against *Haemonchus contortus*	Experimental Parasitology	01	Focus on other areas of study
Leite, PM; Miranda, APN; Gomes, I; Rodrigues, ML; Amorim, JM; de Lima, GATD; Duarte, RCF; Fuly, AL; Faraco, AAG; Carvalho, MD; Castilho, RO	Anticoagulant and antiplatelet activity of aqueous extracts of *Citrus sinensis* and *Lippia alba*: interactions and potential for the development of antithrombotics	Blood Coagulation & Fibrinolysis	01	Focus on other areas of study
Bottignon, MR; Rufino, ER; Marques, MOM; Colombo, CA; de Azevedo, JA; Lourençao, AL; Martins, ALM; Siqueira, WJ	Heterogeneity of linalool chemotypes of *Lippia alba* (Mill.) NEBr., based on clonal half-sib progenies	Scientia Agricola	04	Phytochemical study
Cavalcanti, MM; Sampaio, TL; Lima, DB; da Costa, MFB; de Zevedo, IEP; Monteiro, ML; Evangelista, JSAM; Bandeira, MAM; Martins, AMC	Essential Oil of *Lippia alba* Protects Against Ischemic- Reperfusion Acute Kidney Injury	Brazilian Archives Of Biology And Technology	0	Focus on other areas of study
de Souza, EL; Lima, ED; Freire, KRD; de Sousa, CP	Inhibitory action of some essential oils and phytochemicals on the growth of various moulds isolated from foods	Brazilian Archives Of Biology And Technology	105	Focus on other areas of study
Zamora, CMP; Michaluk, AG; Chiappetta, DA; Nuñez, MB	Herbal buccal films with *in vitro* antibacterial and anti-inflammatory effects	Journal Of Herbal Medicine	11	Focus on other areas of study
Neto, LP; Silva-Santos, L; de Souza, LM; de Morais, MB; Corte-Real, N; Monte, IP Jr; Camara, CAG; Moraes, MM; Ulisses, C	Influence of Arbuscular *Mycorrhizal Fungi* on Morphophysiological Responses and Secondary Metabolism *in Lippia alba* (Verbenaceae) Under Different Water Regimes	Journal Of Plant Growth Regulation	07	Plant physiology study

Authors	Article Title	Journal	WoS-CC citations	Reason for exclusion
Becker, AG; Parodi, TV; Zeppenfeld, CC; Salbego, J; Cunha, MA; Heldwein, CG; Loro, VL; Heinzmann, BM; Baldisserotto, B	Pre-sedation and transport of *Rhamdia quelen* in water containing essential oil of *Lippia alba*: metabolic and physiological responses	Fish Physiology And Biochemistry	27	Study in aquaculture
Peixoto, MG; Bacci, L; Blank, AF; Araújo, APA; Alves, PB; Silva, JHS; Santos, AA; Oliveira, AP; da Costa, AS; Arrigoni-Blank, MD	Toxicity and repellency of essential oils of *Lippia alba* chemotypes and their major monoterpenes against stored grain insects	Industrial Crops And Products	64	Focus on Agricultural Sciences
Benelli, G; Pavela, R; Giordani, C; Casettari, L; Curzi, G; Cappellacci, L; Petrelli, R; Maggi, F	Acute and sub-lethal toxicity of eight essential oils of commercial interest against the filariasis mosquito *Culex quinquefasciatus* and the housefly *Musca domestica*	Industrial Crops And Products	116	Bioplaguicide study
Rodenak-Kladniew, B; Castro, MA; Gambaro, RC; Girotti, J; Cisneros, JS; Viña, S; Padula, G; Crespo, R; Castro, GR; Gehring, S; Chain, CY; Islan, GA	Cytotoxic Screening and Enhanced Anticancer Activity of *Lippia alba* and *Clinopodium nepeta* Essential Oils-Loaded Biocompatible Lipid Nanoparticles against Lung and Colon Cancer Cells	Pharmaceutics	08	Focus on other areas of study
Sobrinho, ACN; de Morais, SM; Marinho, MM; de Souza, NV; Lima, DM	Antiviral activity on the Zika virus and larvicidal activity on the *Aedes spp*. of *Lippia alba* essential oil and β-caryophyllene	Industrial Crops And Products	23	Focus on other areas of study
Coulibaly, FH; Rossignol, M; Haddad, M; Carrasco, D; Azokou, A; Valente, A; Ginibre, C; Kone, MW; Chandre, F	Biological effects of *Lippia alba* essential oil against *Anopheles gambiae* and *Aedes aegypti*	Scientific Reports	01	Focus on other areas of study
Duarte, MCT; Figueira, GM; Sartoratto, A; Rehder, VLG; Delarmelina, C	Anti-Candida activity of Brazilian medicinal plants	Journal Of Ethnopharmacology	407	Focus on other areas of study
Silva-Santos, L; Neto, LP; Corte-Real, N; Sperandio, MVL; Camara, CAG; Moraes, MM; Ulisses, C	Yeast extract and chitosan elicitation improves essential oil, regulates plant growth and antioxidative system in *Lippia alba* (Mill) NE Brown (Verbenaceae)	South African Journal Of Botany	0	Plant physiology study
Tofiño-Rivera, A; Ortega-Cuadros, M; Galvis-Pareja, D; Jiménez-Rios, H; Merini, LJ; Martínez-Pabón, MC	Effect of *Lippia alba* and *Cymbopogon citratus* essential oils on biofilms of Streptococcus mutans and cytotoxicity in CHO cells	Journal Of Ethnopharmacology	31	Focus on other areas of study

Authors	Article Title	Journal	WoS-CC citations	Reason for exclusion
Rucinque, DS; Pulecio-Santos, SL; Viegas, EMM	Impact of Pre-Slaughter Methods on the Overall Quality of Nile Tilapia (*Oreochromis niloticus)*	Journal Of Aquatic Food Product Technology	0	Study in aquaculture
Soares, CHD; Damasceno, PC; Campos, ID; Amorim, GTD; do Carmo, MGF; Chaves, DSD; de Souza, MAA	Selection of genotypes (citral chemotype) of *Lippia alba* (Mill.) N. E. Brown regarding seasonal stability of the essential oils chemical profile	Industrial Crops And Products	04	Phytochemical study
Santos, CP; Rocha, DS; Bajay, MM; Santos, FRC; Campos, JB; Pinheiro, JB; Zucchi, MI; Silva-Mann, R; Arrigoni-Blank, MF; Blank, AF	Cross-species transferability of microsatellite markers in the genus *Lippia*	Genetics And Molecular Research	03	Botanical study
de Abreu, MS; Costa, F; Giacomini, ACVV; Demin, KA; Petersen, EV; Rosemberg, DB; Kalueff, AV	Exploring CNS Effects of American Traditional Medicines using Zebrafish Models	Current Neuropharmacology	02	*Lippia alba* is not the focus of the paper
Supuran, CT; Alcaro, S	Active Components of Essential Oils as Anti-Obesity Potential Drugs Investigated by *in Silico* Techniques	Journal Of Agricultural And Food Chemistry	15	*Lippia alba* is not the focus of the paper
Neto, LP; Silva-Santos, L; Souza, L; Morais, M; Corte-Real, N; Monte, IP; da Camara, CAG; de Moraes, MM; Ulisses, C	Mycorrhization changes the antioxidant response and chemical profile of *Lippia alba* (Verbenaceae) essential oil under salinity conditions	South African Journal Of Botany	01	Plant physiology study
Lermen, C; da Cruz, RMS; de Souza, JS; Marchi, BD; Alberton, O	Growth of *Lippia alba* (Mill.) N. E. Brown inoculated with arbuscular mycorrhizal fungi with different levels of humic substances and phosphorus in the soil	Journal Of Applied Research On Medicinal And Aromatic Plants	14	Plant physiology study
Cervantes-Ceballos, L; Caballero-Gallardo, K; Olivero-Verber, J	Repellent and Anti-quorum Sensing Activity of Six Aromatic Plants Occurring in Colombia	Natural Product Communications	07	Focus on Agricultural Sciences
Castillo, VDL; Guzmán, WCH; Maza, LDR; García, MIB	Inclusion of High-School Students (Diversified Academic Tract) in Natural Plant Bioactivity Research	Revista Educacion	0	Focus on other areas of study
Teves, JFC; Ragaza, JA.	The quest for indigenous aquafeed ingredients: a review	Reviews In Aquaculture	32	Focus on other areas of study
Özek, T; Tabanca, N; Demirci, F; Wedge, DE; Baser, KHC	Enantiomeric Distribution of Some Linalool Containing Essential Oils and Their Biological Activities	Records Of Natural Products	72	Focus on other areas of study

Authors	Article Title	Journal	WoS-CC citations	Reason for exclusion
Andrés, MF; González-Coloma, A; Sanz, J; Burillo, J; Sainz, P	Nematicidal activity of essential oils: a review	Phytochemistry Reviews	156	Focus on Agricultural Sciences
Torres, NL; Zapata, A; Torres, M; Santana, A; Morales, B; Martinez, JL	Diversity and uses of medicinal plants in Panama	Boletin Latinoamericano y Del Caribe de Plantas Medicinales y Aromaticas	01	Focus on other areas of study
Silva, FF; Soares, MCSC; Alves, LC; Lima, MM; Silva, LVA; Faustino, MAG; Silva, FF	Comparative evaluation of the efficacy of phythotherapeutics and chemical products against tick in the control of the *Boophilus microplus* (Canestrini, 1887) through engorged female bioassey	Medicina Veterinaria-Recife	03	Focus on other areas of study
Costa, CMD; da Cruz, MG; Lima, TBC; Ferreira, LC; Ventura, AS; Brandao, FR; Chagas, EC; Chaves, FCM; Martins, ML; Jerônimo, GT	Efficacy of the essential oils of *Mentha piperita*, *Lippia alba* and *Zingiber officinale* to control the acanthocephalan *Neoechinorhynchus buttnerae* in *Colossoma macropomum*	Aquaculture Reports	26	Study in aquaculture
Barros, LDP; da Cruz, EDS; Guimaraes, BD; Setzer, WN; Moura, RHV; Costa, JSD; Silva, JKDD; Figueiredo, PLB	Chemometric analysis of the seasonal variation in the essential oil composition and antioxidant activity of a new geraniol chemotype of *Lippia alba* (Mill.) NEBr. ex Britton & P. Wilson from the Brazilian Amazon	Biochemical Systematics And Ecology	10	Phytochemical study
Ariotti, K; Marcon, JL; Finamor, IA; Bressan, CA; de Lima, CL; Souza, CD; Caron, BO; Heizmann, BM; Baldisserotto, B; Pavanato, MA	Lippia alba essential oil improves water quality during transport and accelerates the recovery of *Potamotrygon wallacei* from the transport-induced stress	Aquaculture	04	study in aquaculture
Huang, YR; Yang, JF; Chi, YY; Gong, C; Yang, HK; Zeng, FX; Gao, F; Hua, XJ; Wang, ZD	Newly Designed Quinazolinone Derivatives as Novel Tyrosinase Inhibitor: Synthesis, Inhibitory Activity, and Mechanism	Molecules	05	Focus on other areas of study
Caceres, A; Alvarez, AV; Ovando, AE; Samayoa, BE	Plants used in Guatemala for the treatment of respiratory-diseases .1. Screening of 68 plants against gram-positive bacteria	Journal Of Ethnopharmacology	65	Ethnopharmacological study

Authors	Article Title	Journal	WoS-CC citations	Reason for exclusion
Torres, NL; Laurido, C; Pavan, MF; Zapata, A; Martínez, JL	Medicinal plants of Panama 2: Ethnobotanics of Forestal Reserve La Tronosa, Province of Los Santos	Boletin Latinoamericano Y Del Caribe De Plantas Medicinales Y Aromaticas	03	Botanical study
Zapata, B; Betancur-Galvis, L; Duran, C; Stashenko, E	Cytotoxic activity of Asteraceae and Verbenaceae family essential oils	Journal Of Essential Oil Research	14	Focus on other areas of study
Bianchini, AE; da Cunha, JA; Brusque, ICM; Pinheiro, CG; Schindler, B; Heinzmann, BM; Baldisserotto, B	Relaxing effect of eugenol and essential oils in *Pomacea canaliculata*	Ciência Rural	02	Focus on other areas of study
Chanotiya, CS; Yadav, A	Enantiomeric Composition of (3R)-(-)- and (3S)-(+)-Linalool in Various Essential Oils of Indian Origin by Enantioselective Capillary Gas Chromatography-Flame Ionization and Mass Spectrometry Detection Methods	Natural Product Communications	16	Phytochemical study
Milho, C; Silva, J; Guimaraes, R; Ferreira, ICFR; Barros, L; Alves, MJ	Antimicrobials from Medicinal Plants: An Emergent Strategy to Control Oral Biofilms	Applied Sciences-Basel	12	Focus on other areas of study
Vargas, ECA; Thixeira, ER; Werneck, YCD; Arantes, DG	The Therapeutic Use of Plants By Users of a Public Pre-Hospital Unit in Campos dos Goytacazes City, Rio De Janeiro State, Brazil	Revista De Pesquisa-Cuidado E Fundamental Online	01	Ethnopharmacological study
Fogliarini, CO; Garlet, QI; Parodi, TV; Becker, AG; Garcia, LO; Heinzmann, BM; Pereira, AMS; Baldisserotto, B	Anesthesia of *Epinephelus marginatus* with essential oil of *Aloysia polystachya*: an approach on blood parameters	Anais Da Academia Brasileira De Ciências	13	Focus on other areas of study
Stashenko, EE; Martínez, JR; Cala, MP; Durán, DC; Caballero, D	Chromatographic and mass spectrometric characterization of essential oils and extracts from *Lippia* (Verbenaceae) aromatic plants	Journal Of Separation Science	51	Phytochemical study
Lobo, RADM; Lobo, ACBNM; de Oliveira, AFM; Andrade, LDC	Ethnomedicinal plants for veterinary use in gypsy communities of the northeast of Brazil	Boletin Latinoamericano Y Del Caribe De Plantas Medicinales Y Aromaticas	01	Ethnopharmacological study
Fereira, EF; Jose, ARS; Bomfim, MP; Porto, JS; de Jesus, JS	Use of plant extracts on control in vitro of *Colletotrichum gloeosporioides* Penz. collected in papaya fruits (*Carica papaya L.)*	Revista Brasileira De Fruticultura	04	Focus on Agricultural Sciences

Authors	Article Title	Journal	WoS-CC citations	Reason for exclusion
Batista, DS; de Castro, KM; Koehler, AD; Porto, BN; da Silva, AR; de Souza, VC; Teixeira, M; Cardoso, MD; Santos, MD; Viccini, LF; Otoni, WC	Elevated CO_2 improves growth, modifies anatomy, and modulates essential oil qualitative production and gene expression in *Lippia alba* (Verbenaceae)	Plant Cell Tissue And Organ Culture	17	Plant physiology study
Heredia-Díaz, Y; García-Díaz, J; López-González, T; Chil-Nuñez, I; Arias-Ramos, D; Escalona-Arranz, JC; González-Fernández, R; Costa-Acosta, J; Suarez-Cruz, D; Sánchez-Torres, M; Martínez-Figueredo, Y	An ethnobotanical survey of medicinal plants used by inhabitants of Holguin, Eastern Region, Cuba	Boletin Latinoamericano Y Del Caribe De Plantas Medicinales Y Aromaticas	11	Ethnopharmacological study
Possamai, MCF; dos Santos, IC; Silva, ES; Gazim, ZC; Gonçalves, JE; Soares, AA; Germano, RD; Fanin, M; de Sá, TC; Otutumi, LK	*In vitro* bacteriostatic activity of *Origanum vulgare, Cymbopogon citratus*, and *Lippia alba* essential oils in cat food bacterial isolates	Semina-Ciências Agrarias	02	Focus on other areas of study
Netto, JDL; Oliveira, RSM ; Copatti, CE	Efficiency of essential oils of *Ocimum basilicum* and *Cymbopogum flexuosus* in the sedation and anaesthesia of Nile tilapia juveniles	Anais Da Academia Brasileira De Ciências	24	Study in aquaculture
Puertas-Mejía, M; Hillebrand, S; Stashenko, E; Winterhalter, P	*In vitro* radical scavenging activity of essential oils from Columbian plants and fractions from oregano (*Origanum vulgare* L.) essential oil	Flavour And Fragrance Journal	75	Phytochemical study
Verloove, F	New records in vascular plants alien to Tenerife (Spain, Canary Islands)	Biodiversity Data Journal	03	Focus on other areas of study
Zeppenfeld, CC; Saccol, EMH; Pês, TS; Salbego, J; Koakoski, G; dos Santos, AC; Heinzmann, BM; Barcellos, LJG; Pavanato, MA; Caron, BO; Baldisserotto, B	*Aloysia triphylla* essential oil as food additive for *Rhamdia quelen* - Stress and antioxidant parameters	Aquaculture Nutrition	20	Focus on other areas of study
Albuquerque, FS; Peso-Aguiar, MC; Assuncao-Albuquerque, MJT	Distribution, feeding behavior and control strategies of the exotic land snail *Achatina fulica* (Gastropoda: Pulmonata) in the northeast of Brazil	Brazilian Journal Of Biology	38	Focus on other areas of study

Authors	Article Title	Journal	WoS-CC citations	Reason for exclusion
Saccol, EMH; Uczay, J; Pês, TS; Finamor, IA; Ourique, GM; Riffel, APK; Schmidt, D; Caron, BO; Heinzmann, BM; Llesuy, SF; Lazzari, R; Baldisserotto, B; Pavanato, MA	Addition of *Lippia alba* (Mill) N. E. Brown essential oil to the diet of the silver catfish: An analysis of growth, metabolic and blood parameters and the antioxidant response	Aquaculture	55	Study in aquaculture
Rodríguez-Sevilla, E; Ramírez-Silva, MT; Romero-Romo, M; Ibarra-Escutia, P; Palomar-Pardavé, M	Electrochemical Quantification of the Antioxidant Capacity of Medicinal Plants Using Biosensors	Sensors	34	Phytochemical study
Sousa, SM; Reis, AC; Gomes, SSL; Bernardo, KB; Salimena, FRG; Viccini, LF	Botanical aspects of *Heteropterys umbellata* (Malpighiaceae): a cytological and palynological approach	Anais Da Academia Brasileira De Ciências	02	Foco em outras áreas de estudo
Palheta, IC; Tavares-Martins, ACC; Lucas, FCA; Jardim, MAG	Ethnobotanical study of medicinal plants in urban home gardens in the city of Abaetetuba, Pará state, Brazil	Boletin Latinoamericano Y Del Caribe De Plantas Medicinales Y Aromaticas	19	Ethnobotanical study
Lermen, C; Cruz, RMSD; Pinc, MM; Zardeto, G; Lorencete, MD; Schwengber, RP; Dias-Arieira, CR; Alberton, O	Essential oil of bushy *Lippia* inoculated with arbuscular mycorrhizal fungi under different levels of humic substances and phosphorus	Rhizosphere	03	Plant physiology study
Rony, MA; Hosen, S; Bashar, MA	Efficacy of chemical fungicides and plant extracts against anthracnose pathogens of *Capsicum frutescens* L.	Bangladesh Journal Of Botany	0	Focus on Agricultural Sciences
Tabari, MA; Jafari, AH; Maggi, F; Mazzara, E; Youssefi, MR; Moghaddas, E; Rostam, MMY; Rezaei, F	Scolicidal activity of some medicinal plant essential oils on *Echinococcus granulosus* protoscolices	Journal Of Essential Oil Research	0	Focus on other areas of study
Rodríguez-Sevilla, E; Ramírez-Silva, MT; Palomar-Pardavé, M; Romero-Romo, MA; Marty, JL; Ibarra-Escutia, P	A novel tyrosinase base biosensor for the quantification of antioxidan capacity.	Chemical And Biological	0	Phytochemical study
Leite, PM; Miranda, APN; Gomes, I; Rodrigues, ML; Camargos, LM; Amorim, JM; Duarte, RCF; Faraco, AAG; Carvalho, MG; Castilho, RO	Antithrombotic potential of *Lippia alba*: A mechanistic approach	Journal Of Ethnopharmacology	03	Focus on other areas of study

Authors	Article Title	Journal	WoS-CC citations	Reason for exclusion
González, ARS; Nuñez, FOM; Villamizar, GAC; Chacón, MI	Molecular characterization of aromatic species of the genus *Lippia* from the *Colombian neotropics*	Proceedings Of The International Symposium On Medicinal And Nutraceutical Plants	05	Botanical study
Sutili, FJ; Gatlin, DM; Heinzmann, BM; Baldisserotto, B	Plant essential oils as fish diet additives: benefits on fish health and stability in feed	Reviews In Aquaculture	113	Study in aquaculture
Pês, TS; Saccol, EMH; Ourique, GM; Londero, EP ; Gressler, LT; Finamor, IA; Rotili, DA; Golombieski, JI; Glanzner, WG; Llesuy, SF; Gonçalves, PBD; Neto, JR; Baldisserotto, B; Pavanato, MA	Effect of diets enriched with rutin on blood parameters, oxidative biomarkers and pituitary hormone expression in silver catfish (*Rhamdia quelen*)	Fish Physiology And Biochemistry	32	Study in aquaculture
Munoz, JA; Staschenko, E; Ocampo, DCB	Insecticidal activity of essential oils from native plants against *Aedes aegypti* (Diptera: Culicidae)	Revista Colombiana De Entomologia	07	Foco em outras áreas de estudo
Lopes, JM; Souza, CD; Saccol, EMH; Pavanato, MA; Antoniazzi, A; Rovani, MT; Heinzmann, BM; Baldisserotto, B	*Citrus x aurantium* essential oil as feed additive improved growth performance, survival, metabolic, and oxidative parameters of silver catfish (*Rhamdia quelen*)	Aquaculture Nutrition	16	Foco em outras áreas de estudo
Ruiz-Duran, J; Torres, R; Stashenko, EE; Ortiz, C	Antifungal and Antibiofilm Activity of Colombian Essential Oils against Different Candida Strains	Antibiotics	04	Focus on other areas of study
Ocazionez, RE; Meneses, R; Torres, FA; Stashenko, E	Virucidal activity of Colombian *Lippia* essential oils on dengue virus replication *in vitro*	Memorias Do Instituto Oswaldo Cruz	53	Focus on other areas of study
de Morais, SM; Cavalcanti, ESB; Costa, SMO; Aguiar, LA	Antioxidant action of teas and seasonings more consumed in Brazil	Brazilian Journal Of Pharmacognosy	20	Phytochemical study
Correia, GA; de Araujo, ML; Carvalho, WA; Sairre, M; Cerchiaro, G; Shul'pina, LS; Kozlov, YN; ShulPin, GB; Kirillov, AM; Mandelli, D	Metal-ligand cooperation in the catalytic oxidation of (R)-carvone by Ga $(NO_3)_3/H_2O_2$1	Molecular Catalysis	01	Phytochemical study
García-Abarrio, SM; Viloria, L; Haya, L; Urieta, JS; Mainar, AM.	Thermophysical behaviour of the mixture (±)-3,7-dimethyl-1,6-octadien-3-ol with etanol	Fluid Phase Equilibria	11	Chemical study
Oliveira, BL; Souza, RM; Silva, JP; Gomes, LC; Fernandes, LFL.	Hematological and biochemical characteristics during the transport of dog snapper *Lutjanus jocu* (Perciformes: Lutjanidae)	Neotropical Ichthyology	03	Study in aquaculture

Authors	Article Title	Journal	WoS-CC citations	Reason for exclusion
Torres, NL; Martínez, JL; Laurido, C; Zapata, A	Medicinal plants of Panama: Ethnobotanics of The Montuoso Forestal Reserve	Boletin Latinoamericano Y Del Caribe De Plantas Medicinales Y Aromaticas	03	Ethnobotanical study
Bastidas-Bacca, MA; Dayve-Bacca-Descance, D; Guerra-Acosta, AD; Perea-Morera, E; Díaz-Ariza, LA; López-Alvarez, D; Osorio-García, AM	Ethnobotanical Insights: Qualitative Analysis of Medicinal Plants in Colon Putumayo for Traditional Knowledge Preservation	Plants-Basel	0	Ethnobotanical study
Zapata, B; Durán, C; Stashenko, E; Betancur-Galvis, L; Mesa-Arango, AC	Antifungal activity, cytotoxicity and composition of essential oils from the *Asteraceae* plant family	Revista Iberoamericana De Micologia	22	*Lippia alba* is not the focus of the paper
Figueroa Gualteros, AM; Castro Triviño, EA; Castro Salazar, HT	Bioplaguicide Effect of Vegetal Extracts to Control of *Spodoptera frugiperda* in Corn Crop (*Zea mays*)	Acta Biologica Colombiana	04	Bioplaguicide study
Sosa, BD; Moro, EB; Gomes, RLM; Cardoso, MD; Cardoso, LM; Boscolo, WR; de Oliveira, JDS; Signor, A; Bittencourt, F	Essential oils in diets for Nile tilapia juveniles: Productive performance and plasmatic biochemistry	Aquaculture Research	03	Study in aquaculture
Pina, LTS; Serafini, MR; Oliveira, MA; Sampaio, LA; Guimaraes, JO; Guimaraes, AG	Carvone and its pharmacological activities: A systematic review	Phytochemistry	16	*Lippia alba* is not the focus of the paper
Espósito, MA; Trento, MVC; Braga, MA; Marcussi, S	Thrombolysis, clotting, and genotoxic activities modulated by essential oils extracted from *Lippia alba*	Brazilian Journal Of Pharmaceutical Sciences	0	Focus on other areas of study
Sutili, FJ; Murari, AL; Silva, LL; Gressler, LT; Heinzmann, BM; de Vargas, AC; Schmidt, D; Baldisserotto, B	The use of *Ocimum americanum* essential oil against the pathogens *Aeromonas hydrophila* and *Gyrodactylus* sp in silver catfish (*Rhamdia quelen*)	Letters In Applied Microbiology	16	Study in aquaculture
Capetti, F; Tacchini, M; Marengo, A; Cagliero, C; Bicchi, C; Rubiolo, P; Sgorbini, B	Citral-Containing Essential Oils as Potential Tyrosinase Inhibitors: A Bio-Guided Fractionation Approach	Plants-Basel	17	*Lippia alba* is not the focus of the paper
Castillo, RM; Stashenko, E; Duque, JE	Insecticidal and repellent activity of several plant-derived essential oils against *Aedes aegypti*	Journal Of The American Mosquito Control Association	53	Focus on other areas of study
Gianoli, E; Palacio-López, K	Phenotypic integration may constrain phenotypic plasticity in plants	Oikos	59	Botanical study

Authors	Article Title	Journal	WoS-CC citations	Reason for exclusion
Teixeira, RR; de Souza, RC; Sena, AC; Baldisserotto, B; Heinzmann, BM; Copatti, CE	Essential oil of *Aloysia triphylla* is effective in nile tilapia transport	Boletim Do Instituto De Pesca	18	Study in aquaculture
de Souza, RC; Baldisserotto, B; Melo, JFB; da Costa, MM; de Souza, EM; Copatti, CE	Dietary *Aloysia triphylla* essential oil on growth performance and biochemical and haematological variables in Nile tilapia	Aquaculture	18	Study in aquaculture
Bastos, JFA; Moreira, IJA; Ribeiro, TP; Medeiros, IA; Antoniolli, AR; De Sousa, DP; Santos, MRV	Hypotensive and Vasorelaxant Effects of Citronellol, a Monoterpene Alcohol, in Rats	Basic & Clinical Pharmacology & Toxicology	90	Focus on *Lippia alba* secondary metabolites
Quintans, LJ; Guimaraes, AG; de Santana, MT; Araújo, BES; Moreira, FV; Bonjardim, LR; Araújo, AAS; Siqueira, JS; Antoniolli, AR; Botelho, MA; Almeida, JRGS; Santos, MRV	Citral reduces nociceptive and inflammatory response in rodents	Brazilian Journal Of Pharmacognosy	38	Focus on *Lippia alba* secondary metabolites
Lobato-Tapia, CA; Aguilar-Muñoz, E	*In silico* analysis of *Aloysia triphylla* compounds with potential anxiolytic activity and prediction of their pharmacokinetic properties	Eneurobiologia	0	*Lippia alba* is not the focus of the paper
Becker, AG; Parodi, TV ; Heldwein, CG; Zeppenfeld, CC; Heinzmann, BM; Baldisserotto, B	Transportation of silver catfish, *Rhamdia quelen*, in water with eugenol and the essential oil of *Lippia alba*	Fish Physiology And Biochemistry	99	study in aquaculture
Usseglio, VL; Dambolena, JS; Zunino, MP	Can Essential Oils Be a Natural Alternative for the Control of *Spodoptera frugiperda*? A Review of Toxicity Methods and Their Modes of Action	Plants-Basel	04	Focus on Agricultural Sciences
Rahmatullah, M; Jahan, R; Azam, FMS; Hossan, S; Mollik, MAH; Rahman, T	Folk medicinal uses of *Verbenaceae* family plants in Bangladesh	African Journal Of Traditional Complementary And Alternative Medicines	31	Ethnopharmacological study
Holetz, FB; Pessini, GL; Sanches, NR; Cortez, DAG; Nakamura, CV; Dias, BP	Screening of some plants used in the Brazilian folk medicine for the treatment of infectious diseases	Memorias Do Instituto Oswaldo Cruz	542	*Lippia alba* is not the focus of the paper

Authors	Article Title	Journal	WoS-CC citations	Reason for exclusion
Pandey, AK; Singh, P; Palni, UT; Tripathi, NN.	*In vitro* antibacterial activities of the essential oils of aromatic plants against *Erwinia herbicola* (Lohnis) and *Pseudomonas putida* (Kris Hamilton)	Journal Of The Serbian Chemical Society	14	*Lippia alba* is not the focus of the paper
Perello, A; Lampugnani, G; Abramoff, C; Slusarenko, A; Bello, G	Suppression of seed-borne *Alternaria arborescens* and growth enhancement of wheat with biorational fungicides	International Journal Of Pest Management	03	Focus on Agricultural Sciences
de Oliveira, GL; de Oliveira, AFM; Andrade, LDC	Medicinal plants used in the urban community of Muribeca, Northeast Brazil	Acta Botânica Brasílica	28	Ethnopharmacological study
Gomide, M; Lemos, F; Reis, D; José, G; Lopes, M; Machado, MA; Alves, T; Coelho, CM	Identification of dysregulated microRNA expression and their potential role in the antiproliferative effect of the essential oils from four different *Lippia* species against the CT26.WT colon tumor cell line	Brazilian Journal Of Pharmacognosy	04	Focus on other areas of study
Junior, GB; de Souza, CF; da Silva, HNP; Bianchini, AE; Rodrigues, P; da Costa, ST; Heinzmann, BM; Cargnelutti, JF; Baldisserotto, B	Combined effect of florfenicol with linalool via bath in combating *Aeromonas hydrophila* infection in silver catfish (*Rhamdia quelen*)	Aquaculture	12	Study in aquaculture
Chung, S; Lemos, CHD; Teixeira, DV; Fortes-Silva, R; Copatti, CE	Essential oil from *Ocimum basilicum* improves growth performance and does not alter biochemical variables related to stress in pirarucu (*Arapaima gigas*)	Anais Da Academia Brasileira De Ciências	12	Study in aquaculture
Bouyahya, A; Mechchate, H; Benali, T; Ghchime, R; Charfi, S; Balahbib, A; Burkov, P; Shariati, MA; Lorenzo, JM; El Omari, N	Health Benefits and Pharmacological Properties of Carvone	Biomolecules	46	Focus on *Lippia alba* secondary metabolites
Can, EK; Kizak, V; Can, SS; Özçiçek, E	Anesthetic potential of geranium (*Pelargonium graveolens*) oil for two cichlid species, *Sciaenochromis fryeri* and *Labidochromis caeruleus*	Aquaculture	17	Study in aquaculture
da Cunha, JA; Scheeren, CA; Salbego, J; Gressler, LT; Madaloz, LM; Bandeira, G; Bianchini, AE; Pinheiro, CG; Bordignon, SAL; Heinzmann, BM; Baldisserotto, B	Essential oils of *Cunila galioides* and *Origanum majorana* as anesthetics for *Rhamdia quelen*: efficacy and effects on ventilation and ionoregulation	Neotropical Ichthyology	19	Study in aquaculture

Authors	Article Title	Journal	WoS-CC citations	Reason for exclusion
Vera, SS; Zambrano, DF; Méndez-Sanchez, SC; Rodríguez-Sanabria, F; Stashenko, EE; Luna, JED	Essential oils with insecticidal activity against larvae of *Aedes aegypti* (Diptera: Culicidae)	Parasitology Research	81	Focus on other areas of study
Dantas, DD; Pereira-de-Morais, L; Silva, AD; da Silva, RER; Dias, FJ; Amorim, TD; Cruz-Martins, N; Coutinho, HDM; Barbosa, R	Pharmacological Screening of Species from the *Lippia genus*, Content in Terpenes and Phenylpropanoids, and their Vasorelaxing Effects on Human Umbilical Artery	Current Pharmaceutical Design	02	Focus on other areas of study
Gonçalves, AT; Llanos-Rivera, A; Ruano, M; Avello, V; Gallardo-Rodriguez, JJ; Astuya-Villalón, A	Physiological Response of Atlantic Salmon (*Salmo salar*) to Long-Term Exposure to an Anesthetic Obtained from *Heterosigma akashiwo*	Toxins	0	Study in aquaculture
Miura, PT; Queiroz, SCN; Jonsson, CM; Chagas, EC; Chaves, FCM; Reyes, FG	Study of the chemical composition and ecotoxicological evaluation of essential oils in *Daphnia magna* with potential use in aquaculture	Aquaculture Research	07	Study in aquaculture
Teixeira, RR; de Souza, RC; Sena, AC; Baldisserotto, B; Heinzmann, BM; Couto, RD; Copatti, CE	Essential oil of *Aloysia triphylla* in Nile tilapia: anaesthesia, stress parameters and sensory evaluation of fillets	Aquaculture Research	51	Study in aquaculture
Parodi, TV; Gressler, LT; Silva, LD; Becker, AG; Schmidt, D; Caron, BO; Heinzmann, BM; Baldisserotto, B	Chemical composition of the essential oil of *Aloysia triphylla* under seasonal influence and its anaesthetic activity in fish	Aquaculture Research	09	Study in aquaculture
Soto-Cáceres, VA; Díaz-Vélez, C; Becerra-Gutiérrez, LK; Arriaga-Deza, EV; Meoño-Asalde, CN; Reyes-Damián, JR; Peña-Vega, KM; Vera-Oblitas, LC; Suyon-Jiménez, JP; Segura-Muñoz, DM; Vargas-Tineo, OW; Silva-Díaz, H	Repellent effect and protection time of essential oils against the adult stage of *Aedes aegypti*	Revista De Investigaciones Veterinarias Del Peru	0	Focus on other areas of study
Lopes, JM; Souza, CD; Schindler, B; Pinheiro, CG; Salbego, J; de Siqueira, JC; Heinzmann, BM; Baldisserotto, B	Essential oils from *Citrus* x *aurantium* and *Citrus* x *latifolia* (Rutaceae) have anesthetic activity and are effective in reducing ion loss in silver catfish (*Rhamdia quelen*)	Neotropical Ichthyology	12	Study in aquaculture

Authors	Article Title	Journal	WoS-CC citations	Reason for exclusion
Silva-Trujillo, L; Quintero-Rueda, E; Stashenko, EE; Conde-Ocazionez, S; Rondón-Villarreal, P; Ocazionez, RE	Essential Oils from Colombian Plants: Antiviral Potential against Dengue Virus Based on Chemical Composition, *In Vitro* and *In Silico* Analyses	Molecules	08	Focus on other areas of study
Baldisserotto, B; Barata, LES; Silva, AS; Lobato, WFF; Silva, LL; Toni, C; Silva, LVF	Anesthesia of tambaqui *Colossoma macropomum* (Characiformes: Serrasalmidae) with the essential oils of *Aniba rosaeodora* and *Aniba parviflora* and their major compound, linalol	Neotropical Ichthyology	23	Study in aquaculture
Wosnick, N; Bendhack, F; Leite, RD; Morais, RN; Freire, CA	Benzocaine-induced stress in the euryhaline teleost, *Centropomus parallelus* and its implications for anesthesia protocols	Comparative Biochemistry And Physiology A-Molecular & Integrative Physiology	04	Study in aquaculture
Ruiz, NQ; Campo, YC; Stashenko, EE; Fuentes, JL	Antigenotoxic Effect Against Ultraviolet Radiation-induced DNA Damage of the Essential Oils from *Lippia* Species	Photochemistry And Photobiology	18	Focus on other areas of study
de Souza, PM; Goulart, FRD; Marques, JM; Bizzo, HR; Blank, AF; Groposo, C; de Sousa, MP; Vólaro, V; Alviano, CS; Moreno, DSA; Seldin, L	Growth Inhibition of Sulfate-Reducing Bacteria in Produced Water from the Petroleum Industry Using Essential Oils	Molecules	12	Focus on other areas of study
de Souza, EM; de Souza, RC; Melo, JFB; da Costa, MM; de Souza, SA; de Souza, AM; Copatti, CE	*Cymbopogon flexuosus* essential oil as an additive improves growth, biochemical and physiological responses and survival against *Aeromonas hydrophila* infection in Nile tilapia	Anais Da Academia Brasileira De Ciências	15	Study in aquaculture
Tabari, MA; Rostami, A; Khodashenas, A; Maggi, F; Petrelli, R; Giordani, C; Tapondjou, LA; Papa, F; Zuo, YT; Cianfaglione, K; Youssefi, MR	Acaricidal activity, mode of action, and persistent efficacy of selected essential oils on the poultry red mite (*Dermanyssus gallinae*)	Food And Chemical Toxicology	25	Focus on other areas of study
de Oliveira, CPB; Lemos, CHD; Silva, AFE; de Souza, SA; Albinati, ACL; Lima, AO; Copatti, CE	Use of eugenol for the anaesthesia and transportation of freshwater angelfish (*Pterophyllum scalare*)	Aquaculture	24	Study in aquaculture

Authors	Article Title	Journal	WoS-CC citations	Reason for exclusion
Ríos, N; Stashenko, EE; Duque, JE	Evaluation of the insecticidal activity of essential oils and their mixtures against *Aedes aegypti* (Diptera: Culicidae)	Revista Brasileira De Entomologia	64	Focus on other areas of study
Moreira, AP; Oliveira, FC; Ferreira, AL; de Almeida, PR; Costa, DC; Cardoso, CAL; Chaves, FCM; Chagas, EC; de Campos, CM	Efficacy of essential oil from ginger (*Zingiber officinale*) for anesthesia and transport sedation of pacu *(Piaractus mesopotamicus)*	Fish Physiology And Biochemistry	0	Study in aquaculture
de Paula, JAM; Silva, MDR; Costa, MP; Diniz, DGA; Sá, FAS; Alves, SF; Costa, ÉA; Lino, RC; de Paula, JR	Phytochemical Analysis and Antimicrobial, Antinociceptive, and Anti-Inflammatory Activities of Two Chemotypes of *Pimenta pseudocaryophyllus* (Myrtaceae)	Evidence-Based Complementary And Alternative Medicine	16	Focus on other areas of study
Saccol, EMH; Parrado-Sanabria, YA; Gagliardi, L; Jerez-Cepa, I; Mourao, RHV; Heinzmann, BM; Baldisserotto, B; Pavanato, MA; Mancera, JM; Martos-Sitcha, JA	*Myrcia sylvatica* essential oil in the diet of gilthead sea bream (*Sparus aurata* L.) attenuates the stress response induced by high stocking density	Aquaculture Nutrition	15	Study in aquaculture
de Souza, EM; de Souza, RC; Melo, JFB; da Costa, MM; de Souza, AM; Copatti, CE	Evaluation of the effects of *Ocimum basilicum* essential oil in Nile tilapia diet: growth, biochemical, intestinal enzymes, haematology, lysozyme and antimicrobial challenges	Aquaculture	36	Study in aquaculture
Baldin, ELL; Crotti, AEM; Wakabayashi, KAL; Silva, JPGF; Aguiar, GP; Souza, ES; Veneziani, RCS; Groppo, M	Plant-derived essential oils affecting settlement and oviposition of *Bemisia tabaci* (Genn.) biotype B on tomato	Journal Of Pest Science	45	Study in aquaculture
Vásquez, SPF; de Mendonça, MS; Noda, SD	Ethnobotany of medicinal plants in riverine communities of the Municipality of Manacapuru, Amazonas, Brasil	Acta Amazonica	51	Ethnopharmacological study
Soria, N; Ramos, P; Viveros, G; Estigarribia, G; Ríos, P; Ortíz, A	Ethnobotany and use of medicinal plants family health units of Caaguazu, Paraguay	Caldasia	04	Ethnopharmacological study
Funari, CS; Carneiro, RL; Khandagale, MM; Cavalheiro, AJ; Hilder, EF	Acetone as a greener alternative to acetonitrile in liquid chromatographic fingerprinting	Journal Of Separation Science	35	Focus on other areas of study

Authors	Article Title	Journal	WoS-CC citations	Reason for exclusion
Barbas, LAL; Pereira-Cardona, PM; Maltez, LC; Garcia, LO; Monserrat, JM; Sampaio, LA	Anaesthesia and transport of juvenile tambaqui *Colossoma macropomum* (Cuvier, 1818) with tricaine methane-sulphonate: Implications on secondary and oxidative stress responses	Journal Of Applied Ichthyology	16	Study in aquaculture
Zeng, XB; Dong, HB; Wu, JR; Wang, WH; Duan, YF; Chen, J; Zhang, JS	Essential oil of *Magnolia denudata* is an effective anesthetic for spotted seabass (*Lateolabrax maculatus*): a test of its effect on blood biochemistry, physiology, and gill morphology	Fish Physiology And Biochemistry	04	Study in aquaculture
Ferreira, RMA; Duarte, JL; Cruz, RAS; Oliveira, AEMFM; Araújo, RS; Carvalho, JCT; Mourao, RHV; Souto, RNP; Fernandes, CP	A herbal oil in water nano-emulsion prepared through an ecofriendly approach affects two tropical disease vectors	Brazilian Journal Of Pharmacognosy	13	Focus on other areas of study
Kizak, V; Can, E; Seyhaneyildiz Can, S	Potential anesthetic properties of Bay Leaf (*Laurus nobilis*) essential oil compared with 2-Phenoxyethanol on Blue Dolphin Cichlid, *Cyrtocara moorii*	Israeli Journal Of Aquaculture-Bamidgeh	02	Study in aquaculture
Mori, NC; Michelotti, BT; Pês, TD; Bressan, CA; Sutili, F; Kreutz, LC; Garlet, Q; Baldisserotto, B; Pavanato, MA; Cerqueira, VR; da Costa, ST; Heinzmann, BM	Citral as a dietary additive for *Centropomus undecimalis* juveniles: Redox, immune innate profiles, liver enzymes and histopathology	Aquaculture	05	Study in aquaculture
Young, T; Walker, SP; Alfaro, AC; Fletcher, LM; Murray, JS; Lulijwa, R; Symonds, J	Impact of acute handling stress, anaesthesia, and euthanasia on fish plasma biochemistry: implications for veterinary screening and metabolomic sampling	Fish Physiology And Biochemistry	34	Study in aquaculture
Becker, AJ; Ramos, PB; Monserrat, JM; Wasielesky, W; Baldisserotto, B	Behavioural and biochemical responses in adult Pacific white shrimp, *Litopenaeus vannamei*, exposed to the essential oil of *Cymbopogon citratus*	Aquaculture Research	05	Study in aquaculture
de Brito, GA; de Oliveira, PFR; Silva, CMD; Neto, MFD; Leite, FHA; Mesquita, PRR; Mota, TF; Magalhaes, JT	Identification of Bioactive Compounds against *Aedes aegypti* (Diptera: Culicidae) by Bioassays and *in Silico* Assays	Chemistry & Biodiversity	10	Focus on other areas of study
Escobar, P; Leal, SM; Herrera, LV; Martinez, JR; Stashenko, E	Chemical composition and antiprotozoal activities of Colombian *Lippia* spp essential oils and their major components	Memorias Do Instituto Oswaldo Cruz	130	Focus on other areas of study

Authors	Article Title	Journal	WoS-CC citations	Reason for exclusion
Daniel, AP; Ferreira, LF; Klein, B; Ruviaro, AR; Quatrin, A; Parodi, TV; Zeppenfeld, CC; Heinzmann, BM; Baldisserotto, B; Emanuelli, T	Oxidative stability during frozen storage of fillets from silver catfish (*Rhamdia quelen*) sedated with the essential oil of *Aloysia triphylla* during transport	Ciência Rural	07	Study in aquaculture
Daniel, AP; Veeck, APL; Klein, B; Ferreira, LF; da Cunha, MA; Parodi, TV; Zeppenfeld, CC; Schmidt, D; Caron, BO; Heinzmann, BM; Baldisserotto, B; Emanuelli, T	Using the Essential Oil of *Aloysia triphylla* (L'Her.) Britton to Sedate Silver Catfish (*Rhamdia quelen*) during Transport Improved the Chemical and Sensory Qualities of the Fish during Storage in Ice	Journal Of Food Science	30	Study in aquaculture
Rodrigues, P; Barbosa, LB; Bianchini, AE; Ferrari, FT; Baldisserotto, B; Heinzmann, BM	Nociceptive-like behavior and analgesia in silver catfish (*Rhamdia quelen*)	Physiology & Behavior	04	Study in aquaculture
Alagöz, K; Parug, S; Tastan, Y; Bilen, S; Sönmez, AY	Spurge (*Euphorbia rigida*) exhibits anaesthetic effect in rainbow trout *(Oncorhynchus mykiss*) without altering plasma cortisol levels	Aquaculture Research	02	Study in aquaculture
Becker, AG; Luz, RK; Mattioli, CC; Nakayama, CL; Silva, WDE; Leme, FDP; Mendes, HCPD; Heinzmann, BM; Baldisserotto, B	Can the essential oil of *Aloysia triphylla* have anesthetic effect and improve the physiological parameters of the carnivorous freshwater catfish *Lophiosilurus alexandri* after transport?	Aquaculture	29	Study in aquaculture
Meneses, R; Ocazionez, RE; Martínez, JR; Stashenko, EE	Inhibitory effect of essential oils obtained from plants grown in Colombia on yellow fever virus replication *in vitro*	Annals Of Clinical Microbiology And Antimicrobials	78	Focus on other areas of study
Zeng, XB; Dong, HB; Yang, YK; Li, T; Li, CH; Zhang, JS	Effects of essential oil of *Magnolia denudata* on spotted seabass (*Lateolabrax maculatus*) during simulated live transportation	Aquaculture	02	Study in aquaculture
Mirghaed, AT; Hoseini, SM; Aydin, B; Paolucci, M; Hoseinifar, SH; Van Doan, H	Effects of anaesthesia with 1,8-cineole on haematological and plasma stress responses in Caspian trout*, Salmo caspius*, subadults	Aquaculture Research	05	Study in aquaculture

Authors	Article Title	Journal	WoS-CC citations	Reason for exclusion
Saccol, EMH; Jerez-Cepa, I; Ourique, GM; Pês, TS; Gressler, LT; Mourao, RHV; Martínez-Rodríguez, G; Mancera, JM; Baldisserotto, B; Pavanato, MA; Martos-Sitcha, JA	*Myrcia sylvatica* essential oil mitigates molecular, biochemical and physiological alterations in *Rhamdia quelen* under different stress events associated to transport	Research In Veterinary Science	26	Study in aquaculture
Saccol, EMH; Toni, C; Pês, TS; Ourique, GM; Gressler, LT; Silva, LVF; Mourao, RHV; Oliveira, RB; Baldisserotto, B; Pavanato, MA	Anaesthetic and antioxidant effects of *Myrcia sylvatica* (G. Mey.) DC. and *Curcuma longa* L. essential oils on tambaqui (*Colossoma macropomum*)	Aquaculture Research	40	Study in aquaculture
dos Santos, GR; Brum, RBCS; de Castro, HG; Gonçalves, CG; Fidelis, RR	Effect of essential oils of medicinal plants on leaf blotch in *Tanzania grass*	Revista Ciência Agronomica	06	Focus on Agricultural Sciences
Anaruma, ND; Schmidt, FL; Duarte, MCT; Figueira, GM; Delarmelina, C; Benato, EA; Sartoratto, A	Control of *Colletotrichum gloeosporioides* (penz.) Sacc. In yellow passion fruit using *Cymbopogon citratus* essential oil	Brazilian Journal Of Microbiology	29	Focus on Agricultural Sciences
Yousefi, M; Vatnikov, YA; Kulikov, EV; Ghelichpour, M	Change in blood stress and antioxidant markers and hydromineral balance of common carp (*Cyprinus carpio*) anaesthetized with citronellal and linalool: Comparison with eugenol	Aquaculture Research	29	Study in aquaculture
Lima, JKA; Albuquerque, ELD; Santos, ACC; Oliveira, AP; Araújo, APA; Blank, AF; Arrigoni-Blank, MD; Alves, PB; Santos, DD; Bacci, L	Biotoxicity of some plant essential oils against the termite *Nasutitermes corniger* (Isoptera: Termitidae)	Industrial Crops And Products	29	Bioplaguicide study
Dubey, S; Ojha, K; Chandrakar, J; Dehariya, R; Vinodia, S; Singh, A; Dixit, AK	Assessment of total phenolic content and antioxidant potentiality of selected Indian folk medicinal plants by spectrophotometric method	Plant Science Today	03	Phytochemical study
Bandeira, G; Pês, TS; Saccol, EMH; Sutili, FJ; Rossi, W; Murari, AL; Heinzmann, BM; Pavanato, MA; de Vargas, AC; Silva, LD; Baldisserotto, B	Potential uses of *Ocimum gratissimum* and *Hesperozygis ringens* essential oils in aquaculture	Industrial Crops And Products	38	Study in aquaculture

**Authors**	**Article Title**	**Journal**	**WoS-CC citations**	**Reason for exclusion**
Jarvis, GE; Barbosa, R; Thompson, AJ	Noncompetitive Inhibition of 5-HT3 Receptors by Citral, Linalool, and Eucalyptol Revealed by Nonlinear Mixed-Effects Modeling	Journal Of Pharmacology And Experimental Therapeutics	29	Focus on other areas of study
Hara, K; Haranishi, Y; Terada, T; Takahashi, Y; Nakamura, M; Sata, T	Effects of intrathecal and intracerebroventricular administration of luteolin in a rat neuropathic pain model	Pharmacology Biochemistry And Behavior	39	Focus on other areas of study
Sangwan, NS; Sharma, PK; Sangwan, RS	Geranyl acetate esterase is commonly present but linalyl acetate esterase occurrence is highly limited in plants	Flavour And Fragrance Journal	08	Phytochemical study
Sousa, EAP; Mendonça, ACAM; Garcia, IR; Lisboa, MAN; Kamdem, JP; Cruz, GV; Silva, MAPD; Fernandes, GP; Júnior, JTC	Ethnoknowledge of medicinal and mystical plants used by healers in Juazeiro do Norte, Ceará, Northeast Brazil	Indian Journal Of Traditional Knowledge	03	Ethnopharmacological study
Mora, J; Blanco-Metzler, H	Evaluation of botanical insecticides in controlling the population of fall armyworms (*Spodopterafrugiperda Smith*) present on corn crops (*Zea mays*) located in Santa Cruz, Guanacaste	International Conference On Organic Agriculture In The Tropics: State Of The Art, Challenges And Opportunities	02	Focus on Agricultural Sciences
Toni, C; Martos-Sitcha, JA; Ruiz-Jarabo, I; Mancera, JM; Martínez-Rodríguez, G; Pinheiro, CG; Heinzmann, BM; Baldisserotto, B	Stress response in silver catfish (*Rhamdia quelen)* exposed to the essential oil of *Hesperozygis ringens*	Fish Physiology And Biochemistry	22	Study in aquaculture
da Silva, LA; Martins, MA; Santo, FE; Oliveira, FC; Chaves, FCM; Chagas, EC; Martins, ML; de Campos, CM	Essential oils of *Ocimum gratissimum* and *Zingiber officinale* as anesthetics for the South American catfish *Pseudoplatystoma reticulatum*	Aquaculture	08	Study in aquaculture
Siqueira, MRP; da Rosa, LC; Santos, RD; Lopes, MPGD; Paumgartten, FJR; Moreira, DD	A newly validated HPLC-DAD-UV method to study the effects of medicinal plants extracts, fractions and isolate compounds on gastric emptying in rodents	Brazilian Journal Of Pharmacognosy	01	Focus on other areas of study
Garlet, QI; Souza, CF; Rodrigues, P; Descovi, SN; Martinez-Rodríguez, G; Baldisserotto, B; Heinzmann, BM	GABAa receptor subunits expression in silver catfish (*Rhamdia quelen*) brain and its modulation by xi Nees essential oil and isolated compounds	Behavioural Brain Research	04	Study in aquaculture

Authors	Article Title	Journal	WoS-CC citations	Reason for exclusion
Farias, PKS; Silva, JCRL; de Souza, CN; da Fonseca, FSA; Brandi, IV; Martins, ER; Azevedo, AM; de Almeida, AC	Antioxidant activity of essential oils from condiment plants and their effect on lactic cultures and pathogenic bacteria	Ciência Rural	11	Focus on other areas of study
Delgado, MN; Gomes, JP; de Castro, RB; de Sousa, JA	Medicinal plants used by residents of urban and rural areas of Luziania, Goiás, Brazil	Revista Agrogeoambiental	0	Ethnopharmacological study
Brandao, FR; Souza, DCD; Sebastiao, FD; Chaves, FCM; Bizzo, HR; O'Sullivan, FLD; Chagas, EC	Essential oils as anaesthetics and sedatives in native Brazilian fish, with a special emphasis on *Colossoma macropomum*: A review	Aquaculture Research	04	Study in aquaculture
Santos, IGD; Scher, R; Rott, MB; Menezes, LR; Costa, EV; Cavalcanti, SCD; Blank, AF; Aguiar, JD; da Silva, TG; Dolabella, SS	Amebicidal activity of the essential oils of *Lippia spp*. (Verbenaceae) against *Acanthamoeba polyphaga* trophozoites	Parasitology Research	17	Focus on other areas of study
Carvalho, GB; Coelho, GCZ; Alves, AC; Silva, APD; Monzani, PS; Senhorini, JA; Vianna, NC; Yasui, GS	Embryo manipulation in neotropical characiform fish: incubation system, anaesthetic, and PGC transplantation in *Prochilodus lineatus*	Zygote	01	Study in aquaculture
Parodi, TV; Cunha, MA; Becker, AG; Zeppenfeld, CC; Martins, DI; Koakoski, G; Barcellos, LG; Heinzmann, BM; Baldisserotto, B	Anesthetic activity of the essential oil of *Aloysia triphylla* and effectiveness in reducing stress during transport of albino and gray strains of silver catfish, *Rhamdia quelen*	Fish Physiology And Biochemistry	101	Study in aquaculture
Ferreira, AL; dos Santos, FAC; Souza, AD; Favero, GC; Pinheiro, CG; Heinzmann, BM; Baldisserotto, B; Luz, RK	Anesthetic and sedative efficacy of essential oil of *Hesperozygis ringens* and the physiological responses of *Oreochromis niloticus* after biometric handling and simulated transport	Fish Physiology And Biochemistry	0	Study in aquaculture
Silva, LD; Garlet, QI; Koakoski, G; de Abreu, MS; Mallmann, CA; Baldisserotto, B; Barcellos, LJG; Heinzmann, BM	Anesthetic activity of the essential oil of *Ocimum americanum* in *Rhamdia quelen* (Quoy & Gaimard, 1824) and its effects on stress parameters	Neotropical Ichthyology	21	Study in aquaculture
Aydin, B; Akhan, S; Gümüs, E; Özbas, M	Anesthetic efficacy of clove oil and 2-phenoxyethanol on doctor fish, *Garra rufa* (Heckel, 1843)	Boletim Do Instituto De Pesca	10	Study in aquaculture
Barbas, LAL; Stringhetta, GR; Garcia, LD; Figueiredo, MRC; Sampaio, LA	Jambu, *Spilanthes acmella* as a novel anaesthetic for juvenile tambaqui, *Colossoma macropomum:* Secondary stress responses during recovery	Aquaculture	27	Study in aquaculture

Authors	Article Title	Journal	WoS-CC citations	Reason for exclusion
Moreira, FJC; Santos, CDG; Innecco, R	Hatching and mortality of second-stage juveniles of *Meloidogyne incognita* race 2 in essential plant oils	Revista Ciência Agronômica	14	Focus on other areas of study
Caballero-Gallardo, K; Quintero-Rincón, P; Stashenko, EE; Olivero-Verbel, J	Photoprotective agents obtained from aromatic plants grown in Colombia: total phenolic content, antioxidant activity, and assessment of cytotoxic potential in cancer cell lines of *Cymbopogon flexuosus* and *Tagetes lucida* cav. Essential oils	Plants-Basel	11	*Lippia alba* is not the focus of the paper
García-Díaz, J; Megret-Depaigne, R; Pérez-Rondón, L; Morales-González, M; Hechavaría-Valdés, Y; Pozo-Revé, Y; Heredia-Díaz, Y	Ethnobotanical characterization of medicinal plants in two communities from the South-eastern Region of Cuba	Caldasia	0	Ethnopharmacological study
Ferreira, AL; Favero, GC; Boaventura, TP; Souza, CD; Ferreira, NS; Descovi, SN; Baldisserotto, B; Heinzmann, BM; Luz, RK	Essential oil of *Ocimum gratissimum* (Linnaeus, 1753): efficacy for anesthesia and transport of *Oreochromis niloticus*	Fish Physiology And Biochemistry	16	Study in aquaculture
Furlani, R; De Sousa, MM; Rocha, GNDAO; Vilar, FCR; Ramalho, RC; Peixoto, RD	Antibacterial activity of essential oils against pathogens of importance in caprine and ovine mastitis	Revista Caatinga	01	Focus on other areas of study
Pérez-Nicolás, M; Vibrans, H; Romero-Manzanares, A	Can the use of medicinal plants motivate forest conservation in the humid mountains of Northern Oaxaca, Mexico?	Botanical Sciences	08	Ethnopharmacological study
Ngugi, CC; Oyoo-Okoth, E; Muchiri, M	Effects of dietary levels of essential oil (EO) extract from bitter lemon (*Citrus limon*) fruit peels on growth, biochemical, haemato-immunological parameters and disease resistance in Juvenile *Labeo victorianus* fingerlings challenged with *Aeromonas hydrophila*	Aquaculture Research	57	Study in aquaculture
Souza, CD; Baldissera, MD; Baldisserotto, B; Heinzmann, BM; Martos-Sitcha, JA; Mancera, JM	Essential oils as stress-reducing agents for fish aquaculture: a review	Frontiers In Physiology	86	Study in aquaculture
Mirghaed, AT; Yasari, M; Mirzargar, SS; Hoseini, SM	Rainbow trout (*Oncorhynchus mykiss*) anesthesia with myrcene: efficacy and physiological responses in comparison with eugenol	Fish Physiology And Biochemistry	29	Study in aquaculture

Authors	Article Title	Journal	WoS-CC citations	Reason for exclusion
Can, E; Kizak, V; Can, SS; Özçiçek, E	Anesthetic efficiency of three medicinal plant oils for aquatic species: coriander *Coriandrum sativum*, linaloe tree *Bursera delpechiana,* and lavender *Lavandula hybrida*	Journal Of Aquatic Animal Health	12	Study in aquaculture
Lima, CMD; Fujishima, MAT; dos Santos, BÉF; Lima, BD; Mastroianni, PC; de Sousa, FFO; da Silva, JO	Phytopharmacovigilance in the elderly: highlights from the Brazilian Amazon	Evidence-Based Complementary And Alternative Medicine	01	Ethnopharmacological study
Barbas, LAL; Maltez, LC; Stringhetta, GR; Garcia, LD; Monserrat, JM; da Silva, DT; Heinzmann, BM; Sampaio, LA	Properties of two plant extractives as anaesthetics and antioxidants for juvenile tambaqui *Colossoma macropomum*	Aquaculture	43	Study in aquaculture
dos Santos, AC; Junior, GB; Zago, DC; Zeppenfeld, CC; da Silvay, DT; Heinzmann, BM; Baldisserotto, B ; da Cunha, MA	Anesthesia and anesthetic action mechanism of essential oils of *Aloysia triphylla* and *Cymbopogon flexuosus* in silver catfish (*Rhamdia quelen*)	Veterinary Anaesthesia And Analgesia	25	Study in aquaculture
Bueno-Sánchez, JG; Martínez-Morales, JR; Stashenko, EE; Ribón, W	Anti-tubercular activity of eleven aromatic and medicinal plants occurring in Colombia	Biomedica	40	*Lippia alba* is not the focus of the paper
Veit, JC; Piccolo, J; Scherer, AF; Machado, IS; Peres, MM; Schwerz, JP; Baldisserotto, B; Heinzmann, BM; Emanuelli, T	Stability of frozen fillets from silver catfish anesthetized withessential oil of *Lippia alba* prior to electrical stunning orhypothermia	Journal Of Food Processing And Preservation	05	Study in aquaculture
da Paz, CA; da Costa, BMPA; Hamoy, MKO; dos Santos, MF; da Rocha, LL; Deiga, YD; Barbosa, AD; do Amaral, ALG; Camara, TM; Barbosa, GB; de Araujo, DB; Hartcopff, PFP; Barbas, LAL; Muto, NA; Hamoy, M	Establishing a safe anesthesia concentration window for Nile tilapia (*Oreochromis niloticus*) (Linnaeus 1758) by monitoring cardiac activity in eugenol immersion baths	Comparative Biochemistry And Physiology C-Toxicology & Pharmacology	01	Study in aquaculture
Mirghaed, AT; Ghelichpour, M; Zargari, A; Yousefi, M	Anaesthetic efficacy and biochemical effects of 1,8-cineole in rainbow trout (*Oncorhynchus mykiss*, Walbaum, 1792)	Aquaculture Research	32	Study in aquaculture

Authors	Article Title	Journal	WoS-CC citations	Reason for exclusion
Almeida, BV; Ribeiro, DA; Santos, MO; de Macedo, DG; Macedo, JGF; Macedo, MJF; de Menezes, IRA; Souza, MMA	Mixtures of medicinal plants from caatinga: Basis for further bioprospecting studies	South African Journal Of Botany	03	Ethnopharmacological study
Valente, CD; dos Santos, G; Becker, AG; Heinzmann, BM; Caron, BO; Baldisserotto, B; Ballester, ELC	Anaesthetic effect of clove basil (*Ocimum gratissimum* L.) essential oil on the giant river prawn (*Macrobrachium rosenbergii*, De Man 1879) exposed to different water pHs	Aquaculture International	01	Study in aquaculture
Yousefi, M; Hoseini, SM; Vatnikov, YA; Nikishov, AA; Kulikov, EV	Thymol as a new anesthetic in common carp (*Cyprinus carpio*): Efficacy and physiological effects in comparison with eugenol	Aquaculture	67	Study in aquaculture
da Silva, AQ; da Silva, DS; Figueiredo, PLB; Sarrazin, SLF; Bouillet, LEM; de Oliveira, RB; Maia, JGS; Mourao, RHV	Seasonal and circadian evaluation of a citral-chemotype from *Lippia alba* essential oil displaying antibacterial activity	Biochemical Systematics And Ecology	17	Focus on other areas of study
Shehabeldine, AM; Salem, SS; Ali, OM; Abd-Elsalam, KA; Elkady, FM; Hashem, AH	Multifunctional silver nanoparticles based on chitosan: antibacterial, antibiofilm, antifungal, antioxidant, and wound-healing activities	Journal Of Fungi	60	*Lippia alba* is not the focus of the paper
Slomp, L; Pereira, PS; França, SD; Zingaretti, S; Beleboni, RO	In vitro nematocidal effects of medicinal plants from São Paulo state, Brazil	Pharmaceutical Biology	09	Focus on Agricultural Sciences
Fontana, DC; Neto, DD; Pretto, MM; Mariotto, AB; Caron, BO; Kulczynski, SM; Schmidt, D	Using essential oils to control diseases in strawberries and peaches	International Journal Of Food Microbiology	17	Focus on Agricultural Sciences
Jerez-Cepa, I; Marín-Rincón, A; Martínez-Rodríguez, G; Ruiz-Jarabo, I; Mancera, JM	A natural additive in the diet to improve growth and reduce energy expenditure of gilthead seabream *(Sparus aurata* L.): Attenuation of high stocking density stress responses	Aquaculture	14	Study in aquaculture
Menetrier, JV; Bonkoski, VR; Medeiros, KA; Estevan, DA; Palozi, RAC; Lívero, FAD; Velasquez, LG; Lourenço, ELB; Gasparotto, A	Ethnomedicinal plants used for the treatment of cardiovascular diseases by healers in the southwestern state of Paraná, Brazil, and their validation based on scientific pharmacological data	Journal Of Religion & Health	03	Ethnopharmacological study

Authors	Article Title	Journal	WoS-CC citations	Reason for exclusion
Saccol, EMH; Londero, ÉP; Bressan, CA; Salbego, J; Gressler, LT; Silva, LVF; Mourao, RHV; Oliveira, RB; Llesuy, SF; Baldisserotto, B; Pavanato, MA	Oxidative and biochemical responses in *Brycon amazonicus* anesthetized and sedated with *Myrcia sylvatica* (G. Mey.) DC. and *Curcuma longa* L. essential oils	Veterinary Anaesthesia And Analgesia	25	Study in aquaculture
Mota, TF; Silva, CMD; Conceciao, MD; Fraga, DBM; Brodskyn, CI; Neto, MFD; Santana, IB; Mesquita, PRR; Leite, FHA; Magalhaes, JT	Screening organic repellent compounds against *Lutzomyia longipalpis* (Diptera: Psychodidae) present in plant essential oils: Bioassay plus an *in silico* approach	Acta Tropica	04	Focus on other areas of study
Martins, AFO; Medeiros, AA; Monteiro, JM; Armstrong, AD; Neto, EMDL	The influence of socio-economic factors on distinct categories of use in a rural community in northeastern Brazil	Acta Botânica Brasílica	0	Ethnopharmacological study
Bodur, T; Afonso, JM; Montero, D; Navarro, A	Assessment of effective dose of new herbal anesthetics in two marine aquaculture species: *Dicentrarchus labrax* and *Argyrosomus regius*	Aquaculture	25	Study in aquaculture
Magara, G; Prearo, M; Vercelli, C; Barbero, R; Micera, M; Botto, A; Caimi, C; Caldaroni, B; Bertea, CM ; Mannino, G; Barcelo, D; Renzi, M; Gasco, L; Re, G; Dondo, A; Elia, AC; Pastorino, P	Modulation of antioxidant defense in farmed rainbow trout (*Oncorhynchus mykiss*) fed with a diet supplemented by the waste derived from the supercritical fluid extraction of basil (*Ocimum basilicum*)	Antioxidants	15	Study in aquaculture
Vilhena, CS; do Nascimento, LAS; Andrade, EHD; da Silva, JKD; Hamoy, M; Torres, MF; Barbas, LAL	Essential oil of *Piper divaricatum* induces a general anaesthesia-like state and loss of skeletal muscle tonus in juvenile tambaqui, *Colossoma macropomum*	Aquaculture	21	Study in aquaculture
dos Santos, AC; Bianchini, AE; Bandeira, G; Garlet, QI; Brasil, MTD; Heinzmann, BM; Baldisserotto, B; Caron, BO; da Cunha, MA	Essential oil of *Aloysia citriodora* Palau and citral: sedative and anesthetic efficacy and safety in *Rhamdia quelen* and *Ctenopharyngodon idella*	Veterinary Anaesthesia And Analgesia	03	Study in aquaculture
Port's, PD; Chisté, RC; Godoy, HT; Prado, MA	The phenolic compounds and the antioxidant potential of infusion of herbs from the Brazilian Amazonian region	Food Research International	41	Phytochemical study

Authors	Article Title	Journal	WoS-CC citations	Reason for exclusion
Rocha, MFG; de Aguiar, FLN; Brilhante, RSN; Cordeiro, RD; Teixeira, CEC; Castelo-Branco, DDCM; Paiva, MDN; Zeferino, JPO; Mafezoli, J; Sampaio, CMD; Barbosa, FG; Sidrim, JJC	*Moringa oleifera* and *Vernonia sp* extracts against *Candida albicans* and *Microsporum canis* isolates from dogs and cats and analysis of toxicity to *Artemia sp.*	Ciência Rural	07	*Lippia alba* is not the focus of the paper
Jerez-Cepa, I; Fernández-Castro, M; O'Neill, TJD; Martos-Sitcha, JA; Martínez-Rodríguez, G; Mancera, JM; Ruiz-Jarabo, I	Transport and recovery of Gilthead Seabream (*Sparus aurata* L.) sedated with clove oil and MS-222: effects on stress axis regulation and intermediary metabolism	Frontiers In Physiology	17	Study in aquaculture
Zeppenfeld, CC; Toni, C; Becker, AG; Miron, DD; Parodi, TV; Heinzmann, BM; Barcellos, LJG; Koakoski, G; da Rosa, JGS; Loro, VL; da Cunha, MA; Baldisserotto, B	Physiological and biochemical responses of silver catfish, *Rhamdia quelen*, after transport in water with essential oil of *Aloysia triphylla* (L'Herit) Britton	Aquaculture	75	Study in aquaculture
Favero, GC; Silva, WDE; Boaventura, TP; Leme, FDP; Luz, RK	Eugenol or salt to mitigate stress during the transport of juvenile *Lophiosilurus alexandri*, a Neotropical carnivorous freshwater catfish	Aquaculture	22	Study in aquaculture
Khumpirapang, N; Chaichit, S; Jiranusornkul, S; Pikulkaew, S; Müllertz, A; Okonogi, S	*In vivo* anesthetic effect and mechanism of action of active compounds from *Alpinia galanga* oil on *Cyprinus carpio* (koi carp)	Aquaculture	12	Study in aquaculture
Wang, BK; Wang, YH; Jia, T; Feng, JX; Qu, CY; Wu, XJ; Yang, XL; Zhang, Q	Changes in physiological responses and immunity of blunt snout bream *Megalobrama amblycephala* from transport stress	Fish Physiology And Biochemistry	04	Study in aquaculture
Bianchini, AE; Garlet, QI; Rodrigues, P; Souza, CD; Silva, LD; dos Santos, AC; Heinzmann, BM; Baldisserotto, B	Pharmacokinetics of S-(+)-linalool in silver catfish (*Rhamdia quelen*) after immersion bath: An anesthetic for aquaculture	Aquaculture	12	Study in aquaculture
Vanderzwalmen, M; Edmonds, E; Carey, P; Snellgrove, D; Sloman, KA	Effect of a water conditioner on ornamental fish behaviour during commercial transport	Aquaculture	11	Study in aquaculture
Bodur, T; Oktavia, IS; Sulmartiwi, L	Effective concentration of herbal anaesthetics *Origanum vulgare* L. oil and its effects on stress parameters in Nile tilapia (*Oreochromis niloticus)*	Veterinary Medicine And Science	0	Study in aquaculture

Authors	Article Title	Journal	WoS-CC citations	Reason for exclusion
Chagas, ACD; Oliveira, MCD; Giglioti, R; Santana, RCM; Bizzo, HR; Gama, PE; Chaves, FCM	Efficacy of 11 Brazilian essential oils on lethality of the cattle tick *Rhipicephalus* (Boophilus) *microplus*	Ticks And Tick-Borne Diseases	40	Bioplaguicide study
Tang, YY; Zhang, HX; Yang, GX; Fang, CL; Kong, C; Tian, LL; Huang, XY	Pharmacokinetics studies of eugenol in Pacific white shrimp (*Litopenaeus vannamei*) after immersion bath	BMC Veterinary Research	07	Study in aquaculture
Baldissera, MD; Souza, CF; Zeppenfeld, CC; Velho, MC; Klein, B; Abbad, LB; Ourique, AF; Wagner, R; Da Silva, AS; Baldisserotto, B	Dietary supplementation with nerolidol nanospheres improves growth, antioxidant status and fillet fatty acid profiles in Nile tilapia: Benefits of nanotechnology for fish health and meat quality	Aquaculture	32	Study in aquaculture
Yousefi, M; Hoseini, SM; Aydin, B; Mirghaed, AT; Kulikov, EV; Drukovsky, SG; Seleznev, SB; Rudenko, PA; Hoseinifar, SH; Van Doan, H	Anesthetic efficacy and hemato-biochemical effects of thymol on juvenile Nile tilapia, *Oreochromis niloticus*	Aquaculture	15	Study in aquaculture
Kizak, V; Can, E; Danabas, D; Can, SS	Evaluation of anesthetic potential of rosewood (*Aniba rosaeodora*) oil as a new anesthetic agent for goldfish *(Carassius auratus*)	Aquaculture	16	Study in aquaculture
Stojanovic, NM; Mladenovic, MZ; Maslovaric, A; Stojiljkovic, NI; Randjelovic, PJ; Radulovic, NS	Lemon balm (*Melissa officinalis* L.) essential oil and citronellal modulate anxiety-related symptoms - *In vitro* and in vivo studies	Journal Of Ethnopharmacology	15	*Lippia alba* is not the focus of the paper
Peixoto, MG; Blank, AF; Arrigoni-Blank, MD; Gagliardi, PR; de Melo, JO; Nizio, DAD; Pinto, VS	Activity of essential oils of *Lippia alba* chemotypes and their major monoterpenes against phytopathogenic fungi	Bioscience Journal	04	Focus on Agricultural Sciences
Magalhaes, KD; Guarniz, WAS; Sá, KM; Freire, AB; Monteiro, MP; Nojosa, RT; Bieski, IGC; Custódio, JB; Balogun, SO; Bandeira, MAM	Medicinal plants of the Caatinga, northeastern Brazil: Ethnopharmacopeia (1980-1990) of the late professor Francisco Jose de Abreu Matos	Journal Of Ethnopharmacology	41	Ethnopharmacological study
González-Ball, R; Bermúdez-Rojas, T; Romero-Vargas, M; Ceuterick, M	Medicinal plants cultivated in urban home gardens in Heredia, Costa Rica	Journal Of Ethnobiology And Ethnomedicine	06	Ethnopharmacological study
de Santana, BF; Voeks, RA; Funch, LS	Ethnomedicinal survey of a maroon community in Brazil's Atlantic tropical forest	Journal Of Ethnopharmacology	44	Ethnopharmacological study

Page 50. Supplementary Information – Table S2. List of Journals.

Journals	Impact Factor	Number of papers	Number of citations
Boletim do Instituto de Pesca	0.5	1	8
Anais da Academia Brasileira de Ciências	1.1	1	8
Aquaculture	3.9	8	332
Biological and Pharmaceutical Bulletin	1.7	1	70
Boletin Latinoamericano y del Caribe de Plantas Medicinales y Aromaticas	0.7	1	0
Brazilian Journal of Biology	*	1	27
Brazilian Journal of Medical and Biological Research	1.9	3	116
Ciencia Rural	0.8	3	27
Comparative Biochemistry and Physiology C: Toxicology & Pharmacology	3.9	1	82
Environmental Science and Pollution Research	*	1	7
Fish Physiology and Biochemistry	2.5	3	82
Fishes	2.1	1	1
Heliyon	3.4	1	0
Journal of Applied Ichthyology	0.7	1	24
Journal of Ethnopharmacology	4.8	4	150
Journal of Experimental Biology	2.8	1	12
Journal of Pharmacy and Pharmacology	2.8	1	19
Marine and Freshwater Behaviour and Physiology	0.9	2	43
Molecules	4.2	4	208
Neotropical Ichthyology	2.0	5	144
Pharmaceutical Biology	3.9	1	45
Phytomedicine	6.7	2	33
Phytotherapy Research	6.1	1	51
Plants-Basel	4.0	1	2
Research in Veterinary Science	2.2	1	43
Reviews in Aquaculture	8.8	1	36
Revista Brasileira de Zootecnia	1.1	1	12
Revista de Biología Tropical	0.8	1	5

*Journals without Impact Factor, considering the Journal Citation Report 2023, Clarivate ®

Page 51. Supplementary Information – Table S3. Science mapping based on selected articles focusing on phytochemical aspects, experimental protocols, and pharmacological procedures of Lippia alba neuropharmacological research.

Authors/ years	Study type	Phytochemical approach			Protocol study	Neuropharmacological mapping		
		Part of the plant used	Major component	Type of extract	Dose and Frequency	Research Objective	Behavioral analysis	CNS-related biological analyses
Silva et al., 2024	*In vitro* (enzimatic test) and *in silico* study	Citral chemotype and carvone chemotype: Leaves; linalool chemotype: Leaves, flowers and fin branches	Citral, carvone and linalool	Essential oil	100 uL/mL in a single application	To evaluate the inhibitory effect on the acetylcholinesterase enzyme and the mechanism of action of essential oil chemotypes of *Lippia alba*	Not applicable	Citral, carvone and linalool chemotype<br>↓ AChE<br>IC-50 between 0,1 a 4,3 µg/mL.
Velasquez et al., 2023	*In vitro* study (Neural cortical cells extrated from rat embryonic brain)	Leaves	Not investigated	Ethanolic	0.01 µg/mL in a single application	To determine the dendritogenic potential of the ethanolic extract from *Lippia alba* leaves	Not applicable	↑ total length of dendrites,<br>↑ number of branches,<br>↑ dendritic complexity,<br>PI3K signaling pathway
Finamor et al., 2023	*In vivo* study (*Potamotrygon Wallacei*)	Leaves	Linalool	Essential oil	10 µL/L in a single application (the essential oil was added to the water tank)	To analyze the effect of *Lippia alba* essential oil on simulated long-term transport on the redox state and brain function of stingrays (*Potamotrygon wallacei*)	Not specified	↓ lactate levels<br>↑ SOD, CAT, GPx, GSH and Gr enzymatic activity<br>↓ lipid peroxidation<br>↓ HSP70 activity<br>↓activation of NF-κB
Nonato et al., 2023	*In vivo* study (*Danio renio*)	Leaves	Not investigated	Ethanolic and Essential oil	4, 20 or 40 mg/kg (essential oil) and 40, 200 or 400 mg/kg (ethanolic extract) both intraperitoneally in a single administration;<br>4 mg/kg (essential oil for GABAergic pathway assessment);<br>400 mg/kg (ethanolic extract for	To investigate the biological activities, including anxiolytic effects, of three species from the *Lippia genus* and their mechanisms of action	Open field test:<br>OE and EXT: ↔ number of crossings<br><br>Light/dark test:<br>OE and EXT: ↑ time permanence in the light zone of the aquarium	Anxiolytic mechanisms of action<br>OE: GABAergic pathway<br>EXT: serotonergic pathway (5-HT3A/3B)

Authors/ years	Study type	Phytochemical approach			Protocol study	Neuropharmacological mapping		
		Part of the plant used	Major component	Type of extract	Dose and Frequency	Research Objective	Behavioral analysis	CNS-related biological analyses
					serotonergic pathway assessment)			
Becker et al., 2023	*In vivo* study (*Hyalella bonariensis)*	Unspecified	Linalool	Essential oil	250, 500 or 750uL/L in a single application (the essential oil was added to the water tank)	To determine the time for anesthetic induction and recovery in Hyalella bonariensis exposed to essential oils, including *Lippia alba*	Highest concentration (750 µL/L): ↓ time to induce stages 1 and 2 of anesthesia Longer anesthetic recovery time<br><br>All concentrations: ↔ locomotor activity ↔ freezing episodes ↔ choice of aquarium zones	Not specified
de Lima et al., 2021	*In vivo* study (*Potamotrygon wallacei*)	Leaves	Not investigated	Essential oil	150, 175, 200 and 225 uL/L in a single application	To evaluate how *Lippia alba* essential oil can influence the anesthetic induction and recovery times of the Amazonian freshwater stingray (*Potamotrygon wallacei)*	Average time for anesthetic induction: between 4.7 and 12.4 min Average time for anesthetic recovery: 8 to 13 min	Not specified
Rucinque et al., 2021	*In vivo* study (*Oreochromis niloticus*)	Unspecified	Linalool	Essential oil	500uL/L in a single application (the essential oil was added to the water tank)	To analyze whether essential oils, including *Lippia alba*, have an anesthetic effect on Nile tilapia	Average time for induction of deep anesthesia: 169.7 s Average recovery time: 134.4 s	↔ plasma cortisol levels
Becker et al., 2021	*In vivo* study (*Farfantepenaeus paulensis* and *Litopenaeus vannamei)*	Unspecified	Linalool	Essential oil	500, 750 or 1000 uL/L in a single application (the essential oil was added to the water tank)	To evaluate the potential anesthetic effects of different essential oils, including *Lippia alba*, on two species of shrimp, *Farfantepenaeus paulensis* and *Litopenaeus vannamei* (Decapoda, Crustacea)	Shortest induction time from sedation to anesthesia: concentration of 1000 $\mu L/L^{-1}$ Recovery time was similar across the administered concentrations	Not specified

Authors/ years	Study type	Phytochemical approach			Protocol study	Neuropharmacological mapping		
		Part of the plant used	Major component	Type of extract	Dose and Frequency	Research Objective	Behavioral analysis	CNS-related biological analyses
Postay et al., 2021	*In vivo* study (*Oreochromis niloticus*)	Leaves	Linalool	Essential oil	250 µL/L in a single application (the essential oil was added to the water tank)	To check whether the use of surfactant can increase the dispersion of *Lippia alba* essential oil in water for adequate sedation of fish (*Oreochromis niloticus*)	Shorten anesthesia induction time: EO + polysorbate 80 (T80) Duration of anesthesia: similar between OE + polysorbate 20 or 80	Not specified
Maia et al., 2019	*In vivo* study (*Colossoma macropomum*)	Leaves, flowers and fin branches	Citral	Hydrolate of essential oil	50, 75, 100 or 150 uL/mL in a single application (the essential oil was added to the water tank)	To evaluate the effects of exposure of juvenile tambaqui (*Colossoma macropomum*) to *Lippia alba* hydrolate on anesthetic induction and recovery times.	All concentrations induced the anesthetic plane (dose-dependent). Recovery time was similar for all concentrations.	Not specified
Souza et al., 2019	*In vivo* study (*Rhamdia quelen*)	Leaves	Citral and Linalool	Essential oil	100uL/L or 300uL/L in a single application (the essential oil was added to the water tank)	To elucidate the involvement of the hypothalamic-pituitary-interrenal (HPI) axis in the anesthetic induction and recovery of two chemotypes of *Lippia alba* essential oil, citral and linalool, in silver catfish.	Not specified	Linalool chemotype ↓Crh, hsd20b, slc6a2, nr3c2 e hsp90. ↑ hsd11b2, Pomca e hspa12a<br><br>Citral chemotype ↓ nr3c2, pomca e hsp90 ↑ Crh, hsd11b2, hsd20b, slc6a2 e hsp12a
Almeida et al., 2019	*In vivo* study (*Serrasalmus eigenmanni)*	Leaves	Not investigated	Essential oil	Induction of anesthesia: 50 - 200uL/L Prolonged induction of anesthesia: 5 - 10uL/L in a single application (the essential oil was added to the water tank)	To investigate whether essential oils, including *Lippia alba*, can be used as anesthetics and stress-reducing agents for the transport of the species *Serrasalmus eigenmanni* Norman	All concentrations reached all stages of anesthesia Similar recovery time at all concentrations ↓ Swimming time ↔ Balance of animals	↔ cortisol levels
da Silva et al., 2019	*In vivo* study (*Colossoma macropomum*)	Leaves	Citral and Linalool	Essential oil	25, 50, 100 or 200uL/L in a single application (the essential oil was	To evaluate the anesthetic effects of essential oils, including *Lippia alba* chemotypes citral and	Sedation induction time - Citral chemotype, 200 µL/L^{-1}: 74.5 s	Not specified

Authors/ years	Study type	Phytochemical approach			Protocol study	Neuropharmacological mapping		
		Part of the plant used	Major component	Type of extract	Dose and Frequency	Research Objective	Behavioral analysis	CNS-related biological analyses
					added to the water tank)	linalool, in juveniles of Tambaqui	- Linalool chemotype, 200 $\mu L/L^{-1}$: 76.1 s<br><br>Deep anesthesia induction time<br>- Citral chemotype, 200 $\mu L/L^{-1}$: 103.6 s<br>- Linalool chemotype, 200 $\mu L/L^{-1}$: 137.25 s<br><br>Recovery time<br>- Citral chemotype, 200 $\mu L/L^{-1}$: 174.45 s<br>- Linalool chemotype, 200 $\mu L/L^{-1}$: 133.08 s	
Batista et al., 2018	*In vivo* study (*Colossoma macropomum*)	Leaves	Citral	Essential oil	Induction of anesthesia: 20, 50, 100, 200 and 300mg/L<br>Stress response during handling: 50 and 100mg/L in a single application (the essential oil was added to the water tank)	To analyze the anesthetic property of *Lippia alba* through the anesthetic induction time and the physiological response to stress in tambaqui (*Colossoma macropomum*)	Stage 2 anesthesia: 20 mg/L^{-1} for 6 hours<br>Stages 3 and 4 of anesthesia: from a dose of 50 mg/L^{-1}<br>Fastest anesthesia time: 200 and 300 mg/L^{-1}, < 4 min.<br>Recovery time was similar for all concentrations<br>↓ Physiological stress (in the highest doses)	Not specified
Souza et al., 2018	*In vivo* study (*Neohelice granulata*)	Leaves	Not investigated	Essential oil	300, 500, 1000, 2000, 3000, 5000 or 8000 uL/L in a single application (the essential oil was	To investigate the anesthetic efficiency of different natural products, including *Lippia alba* essential oil, in the	No anesthetic effect<br>100% mortality	Not specified

Authors/ years	Study type	Phytochemical approach			Protocol study	Neuropharmacological mapping		
		**Part of the plant used**	**Major component**	**Type of extract**	**Dose and Frequency**	**Research Objective**	**Behavioral analysis**	**CNS-related biological analyses**
					added to the water tank)	brachyuran crab *Neohelice granulata*		
da Silva et al., 2018	*In vivo* study (*Drosophila melanogaster*)	Leaves and fin branches	Citral	Essential oil	0,5 a 1,0uL/L in a single application (the essential oil was added to the water tank)	To evaluate whether *Lippia alba* essential oil and its components have sedative activity and influence synaptic transmission in *Drosophila*	Anesthetic effect was proportional to the increase in EO volume<br>Average anesthetic recovery time: 6 hours	OE: ↓ EPSP amplitude<br>↔ EPSP τ<br>↓ RMP<br>Citral: ↓ EPSP amplitude<br>↔ EPSP τ<br>↓ RMP<br>↓ Transmitter release<br>↓ Presynaptic Ca^{2+} influx<br><br>Carvona: ↓ EPSP amplitude<br>↔ EPSP τ<br>↔ RMP
Becker et al., 2018	*In vivo* study (*Rhamdia quelen*)	Leaves	Citral and Linalool	Essential oil	100 - 300uL/L in a single application (the essential oil was added to the water tank)	Verify the effectiveness of *Lippia alba* essential oil as an anesthetic in fish	Higher concentrations: ↔ sedation induction time<br>↑ EO concentration ↓ anesthesia induction time<br>↑ EO concentration ↑ anesthetic recovery time	Not specified
Almeida et al., 2018	*In vivo* study (*Serrasalmus rhombeus)*	Leaves	Linalool	Essential oil	50, 100 and 200uL/L in a single application (the essential oil was added to the water tank)	To analyze the anesthetic efficacy and swimming behavior of *Serrasalmus rhombeus* treated with essential oils, including *Lippia alba*	Induction time for sedation: 15 minutes, 50 μL L^{-1}<br>Deep anesthesia: 200 μL L^{-1}<br>Anesthetic recovery time: proportional to the concentration ↑<br>↓ Swimming time (5 to 10 μL L^{-1})<br>↔ Equilibrium (5 to 10 μL L^{-1})	Not specified

Authors/ years	Study type	Phytochemical approach			Protocol study	Neuropharmacological mapping		
		**Part of the plant used**	**Major component**	**Type of extract**	**Dose and Frequency**	**Research Objective**	**Behavioral analysis**	**CNS-related biological analyses**
Bandeira et al., 2018	*In vivo* study (*Danio renio and Rhamdia quelen*)	Leaves	Linalool	Essential oil	150uL/L in a single application (the essential oil was added to the water tank)	To investigate the aversiveness or attractiveness of essential oils, including *Lippia alba*, in two species of fish to consolidate their anesthetic effect	↔ Change of preference<br>↔ Locomotor parameters<br>No signs of aversiveness<br><br>Anxiolytic effect<br>↑ Distance traveled<br>↑ Mean speed<br>↑ Number of crossings<br>↑ Entries to the upper zone of the aquarium	↓ Plasma cortisol levels only in zebrafish
Salbego et al., 2017	*In vivo* study (*Rhamdia quelen*)	Leaves	Unspecified	Essential oil	10 or 20uL/L in a single application (the essential oil was added to the water tank)	To evaluate the sedative effects of *Lippia alba* essential oil in fish on metabolic responses and acetylcholinesterase activity	Not specified	Cerebral AChE in all concentrations
Salbego et al., 2017	*In vivo* study (*Hypsiboas Geographicus*)	Leaves	Linalool and citral	Essential oil	Chemoype citral: 25, 50, 100 or 200uL/L Chemotype linalol: 50, 75, 100 or 200uL/L in a single application (the essential oil was added to the water tank)	To analyze the sedative and anesthetic activities of essential oils, including *Lippia alba* (citral and linalool chemotype), in tadpoles of *Hypsiboas geographicus*	The higher the concentration, the shorter the sedation and anesthesia induction time, and the longer the recovery time.	Not specified
Simoes et al., 2017	*In vivo* study (*Equinemetra lucunter*)	Leaves	Linalool	Essential oil	50, 100 or 150uL/L in a single application (the essential oil was added to the water tank)	To verify the sedative and anesthetic effect of *Lippia alba* essential oil on *Echinometra lucunter*	Sedation induction: all concentrations, dose-dependent<br>Induction of anesthesia: only the largest doses<br>Effective concentration: 150 $\mu L\ L^{-1}$<br>↔ Recovery time	Not specified

Authors/ years	Study type	Phytochemical approach			Protocol study	Neuropharmacological mapping		
		Part of the plant used	Major component	Type of extract	Dose and Frequency	Research Objective	Behavioral analysis	CNS-related biological analyses
Souza et al., 2017	*In vivo* study (*Rhamdia quelen*)	Leaves	Linalool and Citral	Essential oil	Anesthesia induction: 25, 50, 100, 200, 300uL/L in a single application Stress-induced physiological effects assessment: 100uL/L for up to 5 min - 300uL/L for up 2 min	To investigate the sedative and anesthetic actions of two chemotypes (citral and linalool) of *Lippia alba* on jundiás (*Rhamdia quelen*)	The citral chemotype induced sedation and anesthesia more quickly at the lowest concentration; however, it had the longest recovery time compared to the linalool chemotype.	Not specified
Sena et al., 2016	*In vivo* study (*Colossoma macropomum*)	Leaves and fin branches	Not investigated	Essential oil	Sedation and anesthetic induction: 10, 20, 50, 75, 100, 200 or 300uL/L Stress response assessment: 200uL/L in a single application (the essential oil was added to the water tank)	To analyze the anesthetic efficacy of *Lippia alba* essential oil in juvenile tambacu (*Piaractus mesopotamicus*×*Colossoma macropomum*).	Sedation induction: all concentrations tested<br><br>Anesthesia induction: 200–300 μL L^{-1} (time between 72.63–76.38 s).<br><br>Recovery time < 30 s (10 e 20 μL L^{-1}) < 60 s (50 μL L^{-1}) 300 s (between 200 and 300 μL L^{-1}) > 428 s (75 ou 100 μL L^{-1}).	Not specified
Cárdenas et al., 2016	*In vivo* study (*Argyrosomus regius*)	Leaves	Unspecified	Essential oil	54, 96 and 160mg/L in a single application (the essential oil was added to the water tank)	To determine the anesthetic efficacy of essential oils, including Lippia alba, in juvenile meagre, *Argyrosomus regius* (Asso, 1801)	All concentrations induced anesthesia in less than 3 minutes Recovery time was less than 5 minutes for all concentrations	↑ Cortisol levels ↔ Pituitary GH

Authors/ years	Study type	Phytochemical approach			Protocol study	Neuropharmacological mapping		
		Part of the plant used	Major component	Type of extract	Dose and Frequency	Research Objective	Behavioral analysis	CNS-related biological analyses
Hohlenwerger et al., 2016	*In vivo* study (*Oreochromis niloticus*)	Leaves	Linalool	Essential oil	To determine the ideal concentration of *Lippia alba* essential oil to induce and recover anesthesia in Nile tilapia.	↑ EO concentrations result in shorter sedation and anesthesia times<br>Sedation and anesthesia begin at a concentration of 200 µL L-1<br>↑ EO concentrations lead to longer recovery times<br>Average recovery time: 247 seconds.	Not specified	
Toni et al., 2015	*In vivo* study (*Sparus aurata*)	Leaves	β-linalool	Essential oil	Anesthetic: 25, 35, 50, 100, 200 and 300uL/L<br>Stress: 35uL/L<br>in a single application (the essential oil was added to the water tank)	To evaluate the anesthetic efficacy of *Lippia alba* essential oil in the anesthetic induction and recovery times, as well as in the hypothalamic and pituitary hormonal expression of sea bream (*Sparus aurata*)	All concentrations induced stage 1 anesthesia<br>Stage 3 was induced only between 50 and 300 $\mu L\ L^{-1}$<br>Recovery time was longer at the lowest dose	↑ Cortisol levels<br>↔ CRH, POMCa e Gh<br>↓CRH-BP<br>↑ POMCb<br>↓ PRL
Souza et al., 2015	*In vivo* study (*Rhamdia quelen*)	Leaves	Linalool	Essential oil	0,25 e 0,50 mL/kg in the feed (to satiation once a day)	To analyze the effects of dietary addition of *Lippia alba* essential oil on *Rhamdia quelen* through metabolic, osmoregulatory, and endocrine parameters.	Not specified	↔ GH<br>↔ PRL<br>↑ SL<br>↔ Cortisol levels
Sousa et al., 2015	*In vivo* study (*Rattus norvegicus*)	Unspecified	Citral	Essential oil	60ug/mL applied to the sciatic nerve once (after euthanasia)	To investigate the actions of *Lippia alba* essential oil on compound action potentials (CAPs) in the sciatic nerve of Wistar rats	Not specified	↓ Nerve conduction<br>Complete block of CAP<br>↓ Conduction velocity<br>Preference for Aβ myelinated sensory fibers and Aγ myelinated motor fibers (small caliber)<br>↓ Nerve excitability<br>↑ Rheobase<br>↑Chronaxy

Authors/ years	Study type	Phytochemical approach			Protocol study	Neuropharmacological mapping		
		Part of the plant used	Major component	Type of extract	Dose and Frequency	Research Objective	Behavioral analysis	CNS-related biological analyses
Heldwein et al., 2014	*In vivo* study (*Rhamdia quelen*)	Leaves	Linalool	Essential oil	50,100 e 300uL/L in a single application (the essential oil was added to the water tank)	To evaluate the anesthetic effect of *Lippia alba* essential oil and linalool, and to investigate the possible involvement of the GABAergic system in their mechanism of action in silver catfish (*Rhamdia quelen*)	Linalool<br>Induction of stage 2 anesthesia: 30 and 60 μL L^{-1}<br>Induction of stage 3 anesthesia: 60 and 180 μL L^{-1}<br>Induction of stage 4 anesthesia: 180 μL L^{-1}<br>↑ Anesthetic recovery time<br><br>OE<br>Induction of stage 2 anesthesia: all concentrations<br>Induction of stage 3 anesthesia: only 50 μL L^{-1}<br>Induction of stage 4 anesthesia: all concentrations<br>↓Anesthetic recovery time	The GABAergic system is exclusively involved in the anesthetic action of the essential oil
Salbego et al., 2014	*In vivo* study (*Rhamdia quelen*)	Leaves	Unspecified	Essential oil	Before transport: 200uL/L<br>After transport: 30uL/L and 40uL/L (the essential oil was added to the water tank)	To investigate whether the rapid sedative effect of *Lippia alba* essential oil interferes with enzymes related to purinergic neurotransmission in the brain of silver catfish (*Rhamdia quelen*)	Not specified	↔ AChE<br>↔ NTPDase<br>↔ 5NUC
Carmona et al., 2013	Clinical study	Leaves	Geraniol-carvenone	Hydroethanolic	1 drop/kg/weight/day twice a day	To evaluate the therapeutic action of the hydroethanolic extract of *Lippia alba* leaves in women with migraine.	HIT<br>↓ total score (from 64 to 59)<br><br>MIDAS	Not specified

Authors/ years	Study type	Phytochemical approach			Protocol study	Neuropharmacological mapping		
		Part of the plant used	Major component	Type of extract	Dose and Frequency	Research Objective	Behavioral analysis	CNS-related biological analyses
							↓ Number of missed days (from 19 dias to 5 dias)<br>↓ Number of headache days (from 20 episodes to 5 episodes)<br>↓ Pain intensity (from 7 to 6)<br>No side effects were observed	
Heldwein et al., 2012	*In vivo* study (*Rhamdia quelen*)	Leaves	Linalool	Essential oil	Anesthesia induction: 50, 100 e 300 uL/L; Reversal of anesthetic effects: 300 uL/L in a single application (the essential oil was added to the water tank)	To evaluate the involvement of the GABAergic system in the anesthetic activity of *Lippia alba* essential oil in silver catfish (*Rhamdia quelen*)	DZP + OELa<br>↓ Time for anesthesia induction<br>Induced stage 4 at the lowest doses<br>↔ Recovery time<br><br>Isolated OELa<br>↑ anesthesia induction time<br>Induced stage 4 at the highest doses<br>↑ Recovery time	Isolated OELa<br>Increased flumazenil water recovery score (GABAergic pathway)
Parodi et al., 2012	*In vivo* study (*Litopenaeus vannamei*)	Unspecified	Linalool	Essential oil	Short-term anestesia, sub-adults: 250, 500, 750 or 1000uL/L; Post-larvae: 400, 500 or 600uL/L<br>Concentration range for transport, sub-adults: 50, 100, 200 or 250uL/L; Post-larvae: 100, 200 or 250uL/L in a single application (the essential oil was added to the water tank)	To check the induction and recovery times from anesthesia using essential oils, including *Lippia alba*, in white shrimp (*Litopenaeus vannamei*)	Shrimp subadults:<br>↓ The time required to induce anesthesia (stages 1 and 2)<br>Deep anesthesia time: 30 min<br>Average anesthetic recovery time: 7 min (dose-dependent)<br><br>Postlarvae:<br>Deep anesthesia time: 9.5 min	Not specified

Authors/ years	Study type	Phytochemical approach			Protocol study	Neuropharmacological mapping		
		Part of the plant used	Major component	Type of extract	Dose and Frequency	Research Objective	Behavioral analysis	CNS-related biological analyses
							Average anesthetic recovery time: 6.5 min	
Hatano et al., 2012	*In vivo* study (*Rattus norvegicus*)	Leaves	Carvone	Essential oil	12,5 and 25mg/Kg injected intraperitoneally for 14 days consecutive days	To investigate the anxiolytic properties of *Lippia alba* essential oil in Wistar rats	OE<br>↓ Avoidance time in the high T test<br>↔ escape measures<br><br>Isolated carvone<br>↓ Avoidance time in the high T test<br>↔ Escape measures	Not specified
Conde et al., 2011	Clinical Study - cohort	Leaves	Geraniol	Hydroethanolic and essential oil	1-1.5 drops/kg/day twice a day	To investigate whether the hydroalcoholic extract of *Lippia alba* reduces the intensity and frequency of headache episodes in patients with migraine	↓ Average pain intensity (from 9 to 2)<br>↓ Average frequency of symptoms (from 3 to 0.25 episodes/week)<br>The median time to clinical response was 11 days<br>80% of patients: ↓ minimum of 50% in pain intensity<br>81.6%: ↓ minimum of 50% in pain frequency.<br>8.3% of patients showed worsening of their clinical condition.<br>No side effects were observed.<br>Some patients have had complete abolition of symptoms.	Not specified

Authors/ years	Study type	Phytochemical approach			Protocol study	Neuropharmacological mapping		
		Part of the plant used	Major component	Type of extract	Dose and Frequency	Research Objective	Behavioral analysis	CNS-related biological analyses
da Cunha et al., 2011	*In vivo* study (*Hippocampus reidi*)	Leaves	Not investigated	Essential oil	Anesthetic induction: 0, 10, 20, 50, 150, 300, 450uL/L Transport: 15uL/L	To identify anesthetic induction and recovery times in seahorses (*Hippocampus reidi*) treated with *Lippia alba* essential oil	Remain at stage 2 of anesthesia: 6 hours, for the lowest doses Induction of stages 3a, 3b, and 4: from a concentration of 50 μL/L^{-1}. Dose-dependent anesthesia. ↔ Anesthesia recovery time between concentrations.	Not specified
da Cunha et al., 2010	*In vivo* study (*Rhamdia quelen*)	Leaves	Not investigated	Essential oil	5, 10, 20, 50, 100, 200, 300, 400 or 50 mg/L in a single application (the essential oil was added to the water tank)	To determine the optimal concentration for inducing and recovering from anesthesia with *Lippia alba* essential oil in *Rhamdia quelen*	Induction of stage 2 anesthesia: concentrations ranging from 10 to 400 μL/L^{-1} Induction of stage 3 anesthesia: initiated at a concentration of 50 μL/L^{-1} Induction of stage 4 anesthesia: starting at a concentration of 100 μL/L^{-1} Anesthesia dependent on dose ↔ Recovery time between concentrations	Not specified
Neto et al., 2009	*In vivo* study (*Rattus norvegicus* and *mus musculus*)	Leaves	Linalool and geranial	Hydroethanolic and essential oil	300mg/Kg injected intraperitoneally a single administration	To evaluate the anticonvulsant activity and the involvement of the GABAergic system as mechanisms of action of different chemotypes of *Lippia alba* in mice and rats.	Linalool and citral chemotypes Protect against PTZ-induced seizures ↓ Reduce percentage of mortality	The citral chemotype ↓ GABA uptake ↓ binding to GABA Dose-dependent

Authors/ years	Study type	Phytochemical approach			Protocol study	Neuropharmacological mapping		
		Part of the plant used	Major component	Type of extract	Dose and Frequency	Research Objective	Behavioral analysis	CNS-related biological analyses
							↔ Latency time for seizures<br>↓ Severity/intensity score<br>↔ Locomotor activity	
Hennebelle et al., 2008	*In silico* study	Leaves	Citral	Hydromethanolic and methanolic	Not applicable	To analyze the neurosedative and antioxidant properties of polyphenols and iridoids from *Lippia alba*	Not specified	At the highest concentration, polar compounds:<br>↓ [3H] flunitrazepam binding to the BZD receptor<br>↓ binding of muscimol to the GABA receptor
Zétola et al., 2002	*In vivo* study (*Mus musculus*)	Leaves, flowers and fin branches (sedative activity in leaves)	Not investigated	Ethanolic	200mg/Kg administrered once orally	To investigate the anticonvulsant, sedative, and myorelaxant effects of liquid and spray-dried extracts from *Lippia alba*	80% liquid and spray-dried extract of L. alba<br>↓ Sleep latency induced by sodium pentobarbital<br>↑ Sleep duration induced by sodium pentobarbital<br>↔ Number of seizures<br>↔ Lethality index<br>↔ Latency for seizures<br>↓Motor performance<br>↑ Number of falls<br>↓ Time of permanence on the bar (rota rod)<br>↔ Rectal temperature<br><br>Liquid extract 40% of L. alba	Not specified

Authors/ years	Study type	Phytochemical approach			Protocol study	Neuropharmacological mapping		
		Part of the plant used	Major component	Type of extract	Dose and Frequency	Research Objective	Behavioral analysis	CNS-related biological analyses
							↑ Latency for seizures	
Viana et al., 2000	*In vivo* study (*Mus musculus*)	Leaves	Citral, myrcene and limonene	Essential oil	50 to 400mg/Kg injected intraperitoneally a single administration	To study the anticonvulsant effects of three chemotypes of essential oils from *Lippia alba* N.E. Brown (Verbenaceae) in mice	Three chemotypes orally and i.p.<br>↑ latency to first seizure<br>↑ latency to death<br><br>OE + DZP<br>Enhancement of the effect<br><br>Citral, beta-myrcene and limonene<br>↑ latency to seizure<br>↑ survival percentage	Not specified
Vale et al., 1999	*In vivo* study (*Mus musculus*)	Leaves	Citral, carvone and limonene	Essential oil	10 mL/Kg injected intraperitoneally a single administration	To investigate the behavioral effects of mice treated with three chemotypes of *Lippia alba* essential oils, clarifying their possible mechanisms of action.	All chemotypes<br>↑ Percentage of open arms entries<br>↑ Time spent in open arms<br>↑ Percentage of time in open arms<br>↔ Number of crossings<br>↓ Number of *rearing* and *grooming*<br>↓ rectal temperature<br><br>Limonene chemotype<br>↓ Time of permanence on the bar (rota rod)	Not specified
Viana et al., 1998	*In vivo* study (*Rattus norvegicus*)	Leaves	Citral and carvone	Essential oil	Hot plate: 1, 10 and 50mg/Kg	To evaluate the antinociceptive and anti-inflammatory activity of	Citral Chemotype<br>↑ Latency in hot plate test	Naloxone did not reverse the central effects of citral chemotype

Authors/ years	Study type	Phytochemical approach			Protocol study	Neuropharmacological mapping		
		**Part of the plant used**	**Major component**	**Type of extract**	**Dose and Frequency**	**Research Objective**	**Behavioral analysis**	**CNS-related biological analyses**
					(administrered intraperitoneally); Acetic acid and formalin test: 0,5, 1 and 10mg/Kg (administrered intraperitoneally)	two chemotypes of *Lippia alba*		

Legend - OE: essential oil; EXT: extract; AChE: Acetylcholinesterase; IC-50: maximal inhibitory concentration; PI3R: fosfatidilinositol3-quinase; GH: growth hormone; SOD: superoxide dismutase enzyme; CAT: catalase enzyme; GPX: glutathione peroxidase enzyme; GSH: glutathione; GR: glutathione reductase; CRH: corticotropin releasing hormone; Hsp70: heat shock protein 70; NF-κB: nuclear factor kappa-light-chain-enhancer of activated B cells; s: seconds; min: minutes; 5HT3A-3B: receptor de serotonina; CRH-BP: corticotropin releasing hormone-binding protein; Hsd20b: 20β-hydroxysteroid dehydrogenase; Slc6a2: solute carrier family 6 (neurotransmitter transporter, noradrenalin) member 2; Nr3c2: mineralocorticoid receptor; Hsp90: heat shock protein 90; Hsd11B2: corticosteroid 11β-dehydrogenase isozyme 2; Hspa12a: heat Shock protein 70 member 12; POMCa: proopiomelanocortin a; POMCb: proopiomelanocortin b; EPSP: excitatory postsynaptic potential; EPSP τ: excitatory postsynaptic potential decay τ; RMP: muscle resting membrane potential; Ca^{2+}: calcium channel; PRL: prolactina; SL: somatolactin; CAP: compound action potentials; NTPDase: ecto-nucleosídeo trifosfato difosfohidrolase (NTPDase); 5NUC: 5'nucleotidase; HIT: Headache Impact Test – 6 Item; MIDAS: Migraine Disability Assessment Scale; DZP: Diazepam; GABA: Gamma-AminoButyric Acid.

Page 66. Supplementary Information – Table S4. Detailed information about *Lippia alba* collection, extraction, and chemical composition

Authors/year	Plant collection	Extraction method	Chemical composition
Silva et al., 2024	LA1 e LA2: Pará, Brazil; LA3: São Paulo, Brazil	Hydrodistillation in a Clevenger-type apparatus (Citral chemotype and carnove chemotype)<br><br>Steam distillation (linalool chemotype)	Citral chemotype (3,7%): 6-methyl-5-Hepten-2-one (1.9%), Mircene (0.9%),p-Cymene (0.77%), Limonene (7.02%), trans-β-Ocimene (0.53%), γ-Terpinene (2.78%), Linalool (0.77%), E-Isocitral (0.63%), Citronelol (1.17%), Neral (23,84%), Carvone (0.8%), Geraniol (1.14%), Geranial (32.31%), β-Elemene (0.64%), E-Caryophyllene (0.58%), γ-Muurolene (7.67%), α-Zingiberene (1.15%), Cubebol (0.58%), Elemol (5.3%).<br><br>Carbona chemotype (1,1%): α-Pinene (0.56%), Sabinene (3.74 %), Mircene (3,89%),Limonene (10.3%),1,8-Cineole (14,37%), trans-β-Ocimene (0.96 %),Linalool (0.75%), cis-Sabinene hydrate (0.52%), trans-Verbenol (0.73%), δ-Terpineol (0.5%), α-Terpineol (2.14%), Carvone (30.7%), Piperitenone (1.34%), β-Cubebene (0.58%), β-Elemene (0.79%), γ-Muurolene (6.45%), Cubebol (1.14%), Elemol (5.21%), Guaiol (0.53%), Cedr-8(15)-en-9-α-ol (1.3%), 8-Cedren-13-ol (1.61%), Curcumenol (0.6%), Myrtenol (0.69%).<br><br>Linalool chemotype(1,3%): trans-β-Ocimene (1.98%), cis-Linalool oxide (1.81%), trans-Linalool oxide (1.58%),Linalool (68.31%), endo-Fenchol (2%), β-Cyclocitral (2,29%),Myrtenol (0.92%),β-Elemene (2.78%), E-Caryophyllene (4.14%), γ-Muurolene (4.13%).
Velasquez et al., 2023	Tolima, Colombia	Percolation with 96% ethanol in 4 cycles of 24h	Tannis, flavonoids and phenolic compounds
Finamor et al., 2023	Rio Grande do Sul, Brazil	Hydrodistillation in a unspecified apparatus	β-linalool (59.9%), cineole (10.29%), germacrene D (6.49%), caryophyllene (4.78%), germacrene B (4.48%).
Nonato et al., 2023	Ceará, Brazil	Hydrodistillation in a Clevenger-type apparatus (Essential oil); maceration in ethanol for 72h (Ethanol extract)	Not investigated
Becker et al., 2023	Rio Grande do Sul, Brazil	Hydrodistillation in a Clevenger-type apparatus	Linalool (59.8%), cineole (10.29%), germacrene D (6.49%), germa-crene B (4.78%) and β-caryophyllene (3.64%).
de Lima et al., 2021	Rio Grande do Sul, Brazil	Hydrodistillation in a Clevenger-type apparatus	Not investigated
Rucinque et al., 2021	Goiás, Brazil	Unspecified	Myrcene (0.1%), δ-carene (0.1%), 1,8-cineole (5.7%), Linalool (74.1%), Isoborneol (0.1%), Camphor (1.5%), α-copaene (0.3%), β-elemene (0.1%), β-caryophyllene (14.1%), α-bergamotene (0.3%), Humulene (1.2%), β-farnesene (0.2%), α-bisabolene (0.4%), Nerolidol (0.6%), caryophyllene oxide (0.7%), α-terpineol (0.3%).
Becker et al., 2021	Rio Grande do Sul, Brazil	Steam distillation in a Clevenger-typer apparatus	Linalool- (58.37%), 1,8-cineole - (6.33%), germacrene D - (4.47%) and β-caryophyllene - (3.64%).

Authors/year	Plant collection	Extraction method	Chemical composition
Postay et al., 2021	Espírito Santo, Brazil	Steam-dragged hydrodistillation in a Clevenger-typer apparatus	Linalool (42.36%), Geraniol (12.46%), Neral (10.7%), Limonene (7.45%), Cineole<1,8-> (3.89%), Terpinene<γ-> (3.65%), Cadina-1(6),4-diene<trans> (2.92%), Cimene<o-> (2.46%), Hepten-2-one<6-methyl-5-> (2.35%), Sabinene (1.46%), Ocimene<(E)-β-> (1.27%), Cariophllene<(E)-> (1.11%), Mircene (0.93%), NI (0.71%), Germacrene B (0.71%), NI (0.62%), Isocitral<(E)-> (0.57%), Octen-3-ol<1-> (0.48%), Thujene<α-> (0.4%), Isocitral<(Z)-> (0.4%), Germacrene A (0.38%), Citronellal (0.33%), Carvone (0.31%), Sabinene hydrate<cis-> (0.29%), Hexenol<(3E)-> (0.24%), Terpineol<α-> (0.24%), Hedycaryol (0.18%), Terpinene<α-> (0.16%), Bulnesol (0.15%), Cubebol (0.14%), Pinene<α-> (0.13%), Cubebene<β-> (0.11%), Ylangene<α-> (0.1%), Humulene<α-> (0.08%), NI (0.07%), Copaene<β-> (0.07%), Cadinene<δ-> (0.07%), Geranyl acetate (0.03%).
Maia et al., 2019	Pará, Brazil	Hydrodistillation in a Clevenger-type apparatus	**Essential oil:** α-Thujene (0.23%), α-Pinene (0.13%), Benzaldehyde (0.28%), Abinene (0.42%), Hepten-2-one-6-methyl-5 (1.29%), α-Terpinene (0.16%), Ortho-cymene (1.26%), Para-cymene (0.64%), Limonene (7.32%), Benzeneacetaldehyde (0.97%), β-Ocimene, E (0.47%), γ-Terpinene (3.02%), Monoterpenehydrocarbon (0.1%), Linalool (0.78%), cis-p-mentha-2,8-dien-1-ol (0.2%), Geijerene (0.23%), Oxygenatedmonoterpene (0.1%), Citronellal (0.11%), cis-Chrysanthenol (0.5%), Borneol (0.2%), Isocitral, E (1.02%), Terpinen-4-ol (1.16%), Dihydrocarveol (0.73%), Myrtenol (0.15%), Neo-dihydrocarveol (0.25%),, cis-Carveol (0.31%), Nerol (0.82%), cis-Sabinenehydrateacetate (0.27%), Citronellol (0.29%), Neral (24.16%), Carvone (7.41%), Geraniol (0.36%), Piperitone (0.32%), Geranial (34.38%),Ni (0.09%), Isobornylacetate (0.09%), Eugenol (0.44%), α-Copaene (0.09%), sesquiterpenehydrocarbon (0.14%), β-Bourbonene (0.2%), β-Cubebene (0.2%), β-Elemene (0.39%), Sesquithujene (0.09%), caryophyllene, E (0.56%), β-copaene (0.15%), α-Guaiene (0.16%), α-Humulene (0.19%), allo-Aromadendrene (0.16%), γ-Muurolene (3.37%), α-Zingiberene (0.62%), α-Muurolene (0.15%), Bicyclogermacrene (0.14%), β-Bisabolene (0.12%), Δ-Amorphene (0.25%), Δ-Cadinene (0.28%), Elemol (3.24%), nerolidol, E (0.38%), Carotol (0.1%), Guaiol (0.32%), β-Atlantol (0.12%). **Hydrolate**: 3-Methyl-4-penten-1-ol (1%), Benzaldehyde (0.28%), Hepten-2-one-6-methyl-5 (3.4%), Para-cymene (0.64%), Benzeneacetaldehyde (0.97%), Linalool (10.99%), Phenylethylalcohol (0.72%), cis-p-mentha-2,8-dien-1-ol (0.2%), Borneol (0.2%), Dihydrocarveol (0.73%), Neo-dihydrocarveol (0.25%), trans-Carveol (0.3%), cis-Carveol (0.31%), Nerol (0.82%), Citronellol (0.43%), Neral (31.23%), Carvone (9.95%), Geraniol (0.55%), Piperitone (0.54%), Geranial (33.43%), Ni (0.59%), Thymol (0.32%), Ni (0.63%), Piperitenone (0.52%), Eugenol (0.44%), Ni (0.1%), Elemol (3.73%), Carotol (0.1%).
Souza et al., 2019	Unspecified	Hydrodistillation in a unspecified apparatus	Not investigated

Authors/year	Plant collection	Extraction method	Chemical composition
Almeida et al., 2019	Rio Grande do Sul, Brazil	Hydrodistillation in a Clevenger-type apparatus	Not investigated
da Silva et al., 2019	Pará, Brazil (citral) and Rio Grande do Sul (linalool)	Unspecified	Not investigated
Batista et al., 2018	Manaus, Brazil	Hydrodistillation in a Clevenger-type apparatus	Myrcene (2.0%), Linalool (1.5%), Terpinen-4-ol (1.2%), Neral (16.6%), Geranial (25.4%), β-Elemenene (2.0%), β-Caryophyllene (6.6%), β-Selinene (1.7%), α-Selinene (1.3%), Caryophyllene oxide (16.0%).
Souza et al., 2018	Rio Grande do Sul, Brazil	Steam distillation in a Clevenger-typer apparatus	Not investigated
da Silva et al., 2018	Pará, Brazil	Hydrodistillation in a Clevenger-type apparatus	α-Thujene (0.23%), α-Pinene (0.13%), Abinene (0.42%), 2-Heptene-6-metil-5 (1.29%), α-Terpinene (1.29%), Cimene ortho (0.16%), Limonene (7.32%), E-β-ocimene (0.47%), γ-Terpinene (3.02%), Linalool (0.78%), Geijerene (0.23%), Citronela (0.11%), Z-Chrysanthenol (0.50%), E-isocitral (1.02%), Myrtenol (0.15%), Z-Sabinene hydrate acetate (0.27%), Citronellol (0.29%), Citral (neral+geranial) (58.54%), Carvone (7.41%), Geraniol (0.36%), Piperitone (0.32%), Isobornyl acetate (0.09%), α-Copaene (0.09%), β-Elemene (0.39%), Sesquitujene (0.09%), β-Caryophyllene (0.56%), β-Copaene (0.15%), α-Guaiene (0.16%), α-Humulene (0.19%), Alloaromadendrene (0.16%), γ-Muurolene (3.37%), α-Zingiberene (0.62%), α-Muurolene (0.15%), Bicyclogermacrene (0.14%), β-Bisabolene (0.12%), δ-Amorfilene (0.25%), δ-Cadinene (0.28%), Elemol (3.24%), E-nerolidol (0.38%), Guaiol (0.32%), β-Atlantol (0.12%), γ-Eudesmol (0.24%), α-Muurulol (0.09%).

Authors/year	Plant collection	Extraction method	Chemical composition
Becker et al., 2018	Pará, Brazil	Hydrodistillation in a Clevenger-type apparatus	Hexenal<2E-> (0.06%), Thujene<alpha-> (0.10%), Benzaldehyde (0.04%), Sabinene (0.35%), Dimethyl-4-heptanone<3.5-> (0.04%), Hepten-2-one<6-methyl-5-> (1.40%), Myrcene (0.31%), Phellandrene<alpha-> (0.10%), Isoamyl isobutyrate (0.03%), Terpinene<alpha-> (0.18%), Cymene<para-> (1.35%), Limonene (9.11%), Cineole<1.8-> (0.04%), Ocimene<(E)-beta-> (0.45%), Bergamal (3.13%), Sabinene hydrate<cis->(IPP vs OH) (0.08%), Terpinolene (0.04%), NI (0.08%), Linalool (0.95%), Pinene oxide<alpha-> (0.12%), NI (0.15%), Geijerene (0.12%), Isocitral<exo-> (0.26%), Necrodol<trans-alpha-> (0.25%), Citronellal (0.34%), Borneol (0.10%), Borneol (0.06%), Terpinen-4-ol (0.27%), eritrol<cis-> (0.05%), Carveol<trans-> (0.05%), Citronellol (1.42%), Mentha-1(7),8-dien-2-ol<cis-p-> (0.14%), Neral (25.26%), Carvone (0.07%), Geraniol (0.17%), Geranial (30.02%), Citronellyl acetate (0.04%), Eugenol (0.13%), Neryl acetate (0.11%), Copaene<alpha-> (0.13%), Geranyl acetate (0.35%), Bourbonene<beta-> (0.11%), Cubebene<beta-> (0.31%), Elemene<beta-> (0.30%), Sesquithujene (0.16%), Cedrene<alpha-> (0.17%), Funebrene<beta-> (0.06%), Caryophyllene(E-) (0.43%), Copaene<beta-> (0.14%), Humulene<alpha-> (0.10%), Farnesene<E-beta-> (0.15%), Aromadendrene<allo-> (0.20%), Muurolene<gamma-> (4.33%), amorphene<gamma-> (1.14%), Muurolene<alpha-> (0.19%), Bisabolene<(Z)-alpha-> (0.13%), Cadinene<delta-> (0.45%), Calamenene<cis-> (0.09%), Copaen-11-ol<alpha-> (0.06%), Elemol (5.24%), Nerolidol<E-> (0.71%), Globulol (0.66%), Cubenol<1,10-di-epi-> (0.05%), Eremoligenol (0.11%), Eudesmol<gamma-> (0.39%), Hinesol (0.05%), Eudesmol<beta-> (0.54%), Eudesmol<alpha-> (0.52%), Bulnesol (0.05%), Germacra-4(15),5,10(14)-trien-1-alpha-ol (0.13%), Bergamotol<(Z)-alpha-trans-> (0.26%), Curcumenol (0.14%), Geranyl isobutanoate (0.19%), NI (0.28%), Selinene<7-epi-alpha-> (0.11%), Isocitral<Z-> (1.10%), Isocitral<E-> (1.51%) and NI (0.23%)
Almeida et al., 2018	Rio Grande do Sul, Brazil	Hydrodistillation in a Clevenger-type apparatus	Sabinene (0.817%), β-Pinene (0.972%), Limonene (1.992%), Eucalyptol (10.633%), Linalool (66.347%), Camphor (0.516%), Trans-Dihydrocarvone (1.183%), Carveol (1.135%), Geranial (0.764%), Neral (0.361%), Aromadendrene (3.480%), Germacrene D (2.784%), Germacrene B (2.219%) and Spathulenol (1.340%).
Bandeira et al., 2018	Rio Grande do Sul, Brazil	Hydrodistillation in a Clevenger-type apparatus	Sabinene (0.45%), β-Pinene (0.77%), Limonene (0.28%), Eucalyptol (3.82%), β-E-ocimene (0.33%), Linalool (81.64%), Carveol (1.39%), α-Citral (2.30%), β-Elemene (0.21%), β-Caryophyllene (1.60%), β-Cubebene (1.87%).
Salbego et al., 2017	Rio Grande do Sul, Brazil	Steam distillation in a Clevenger-typer apparatus	Sabinene (0.45%), β-Pinene (0.77%), Limonene (0.28%), Eucalyptol (3.82%), β-E-ocimene (0.33%), Linalool (81.64%), Carveol (1.39%), α-Citral (2.30%), β-Elemene (0.21%), β-Caryophyllene (1.60%), β-Cubebene (1.87%).
Salbego et al., 2017	Pará, Brazil (citral) and Rio Grande do Sul (linalool)	Hydrodistillation in a Clevenger+F29:F36-type apparatus	Linalool (50,6%) and citral (54,4%).

Authors/year	Plant collection	Extraction method	Chemical composition
Simoes et al., 2017	Rio Grande do Sul, Brazil	Hydrodistillation in a Clevenger-type apparatus	α-Pinene (0.52%), 5-hepten-2one-4,6-dimethyl (1.32%), sabinene (0.30%), β-Pinene (0.61%), β-Myrcene (9.74%), limonene (0.53%), eucalyptol (10.51%), β-E-ocimene (4.86%), linalool (48.69%), 2.6-dimethyl-1,3,5,7-octatretaene,E,E (2.81%), Camphor (1.86%), α-terpineol (0.19%), E-dihydrocarvone (0.47%), γ-terpineol (0.48%), Z-dihydrocarvone (0.70%), E-Carveol (0.86%), neral (0.77%), geranial (1.23%), total of monoterpenoids (86.45%), Copaene (0.33%), β-elemene (2.06%), β-Caryophyllene (4.19%), α-Caryophyllene (1.30%), β-Z-Farnesene (0.22%), γ-Muurolene (1.00%), germacrenea (0.78%), γ-Cadinene (0.49%), δ-Cadinene (0.86%), germacrene B (0.22%), E-nerolidol (33.15%), Caryophyllene oxide (0.40%).
Souza et al., 2017	Rio Grande do Sul, Brazil and Pará, Brazil	Hydrodistillation in a Clevenger-type apparatus	Linalool chemotype: Sabinene (1.05%), limonene (0.63%), 1,8-cineole (7.01%), E-β-ocimene (1.10%), β-linalool (50.56%), Z-geraniol (0.49%), E-geraniol (0.17%), E-citral (1.51%), β-elemene (2.66%), E-caryophyllene (4.56%), γ-elemene (1.27%), γ-muurolene (5.23%), bicyclogermacrene (0.22%), elemol (0.13%), germacrene B (2.37%), caryophyllene oxide (1.12%).<br>Citral chemotype: Sabinene (0.47%), limonene (6.15%), E-β-ocimene (0.35%), γ-terpinene (3.16%), β-linalool (0.73%), Z-geraniol (3.57%), Z-citral (24.41%), E-geraniol (5.32%), E-citral (29.84%), β-elemene (0.30%), E-caryophyllene (0.99%), γ-elemene (0.08%), γ-muurolene (2.46%), bicyclogermacrene (3.72%), elemol (3.15%), germacrene B (0.23%), caryophyllene oxide (0.80%).
Sena et al., 2016	Rio Grande do Sul, Brazil	Unspecified	Not investigated
Cárdenas et al., 2016	Rio Grande do Sul, Brazil	Hydrodistillation in a Clevenger-type apparatus	Not investigated
Hohlenwerger et al., 2016	Rio Grande do Sul, Brazil	Steam distillation in a Clevenger-type apparatus	Sabinene (0.83%), β-myrcene (11.02%), limonene (0.58%), eucalyptol (9.77%), β-E-ocimene (5.44%), linalool (47.66%), 2,6-dimethyl-1,3,5,7-octatetraene,E,E (3.50%), camphor (0.61%), umbellulone (0.33%), NI (0.97%), α-terpineol (0.28%), Z-dihydrocarvone (0.40%), γ-terpineol (0.81%), E-dihydrocarvone (1.15%), E-carveol (0.60%), neral (1.03%), geranial (1.78%), NI (0.16%), copaene (0.25%), β-elemene (2.12%), β-caryophyllene (4.04%), α-caryophyllene (1.39%), Z-β-farnesene (0.19%), allo-aromadendrene (0.79%), γ-muurolene (0.94%), germacrenea (0.77%), γ-cadinene (0.40%), δ-cadinene (0.87%), germacrene B (0.22%), longicamphenylone (0.25%), caryophyllene oxide (0.39%).

Authors/year	Plant collection	Extraction method	Chemical composition
Toni et al., 2015	Rio Grande do Sul, Brazil	Hydrodistillation in a Clevenger-type apparatus	Sabinense (0.534%), 1-octen-3-ol (0.094%), β-myrcene (0.152%), Limonene (0.08%), 1-8-cineol (3.576%), E-β-ocimene (0.214%), E-linalool oxide (furanoid) (0.073%), β-linalool (87.605%), Hotrienol (0.097%), 1,3,8-p-menthatriene (0.062%), NI (0.116%), Camphor (0.303%), NI (0.067%), Pinocarvone (0.063%), p-mentha-1,5-dien-8-ol (0.148%), α-terpineol (0.219%), Z,Z-2,6-dimethyl-3,5,7-octatrien-2-ol (0.385%), E,E-2,6-dimethyl-3,5,7-octatrien-2-ol (0.779%), Z-citral (0.096%), E-citral (0.102%), exo-2-hydroxycineole acetate (0.078%), Isoledene (0.063%), β-bourbonene (0.078%), NI (0.4%), β-caryophyllene (1.019%), γ-elemene (0.167%), α-caryophyllene (0.143%), E-β-farnesene (0.097%), γ-muurolene (1.226%), NI (0.184%), NIE (0.106%), NI (0.584%), E-nerolidol (0.146%), caryophyllene oxid (0.619%), NI (0.203%), NI (0.12%).
Souza et al., 2015	Rio Grande do Sul, Brazil	Hydrodistillation in a Clevenger-type apparatus	Not investigated
Sousa et al., 2015	Ceará, Brazil	Unspecified	Citral (75%) [geranial (41.81%), neral (34.11%)], 1-limoneno (9.85%), carvone (8.92%), gamma-terpinene (2.05%), benzene, and 1-methyl-3-(1-methylethyl) (1.02%).
Heldwein et al., 2014	Rio Grande do Sul, Brazil	Hydrodistillation in a Clevenger-type apparatus	Not investigated
Salbego et al., 2014	Rio Grande do Sul, Brazil	Hydrodistillation in a Clevenger-type apparatus	Not investigated
Carmona et al., 2013	Minas Gerais, Brazil	Maceration in a 70% ethanol for 10 days	Not investigated

Authors/year	Plant collection	Extraction method	Chemical composition
Heldwein et al., 2012	Rio Grande do Sul, Brazil	Hydrodistillation in a Clevenger-type apparatus	α-pinene (0.13%), camphene (0.20%), α-phellandrene (1.32%), sabinene (0.22%), 1-octen-3-ol (0.10%), myrcene (0.84%), limonene (0.48%), 1,8-cineole (9.11%), β-ocimene,Z (0.09%), β-ocimene,E (0.67%), γ-terpinene (0.03%), sabinene hydrate,Z (0.14%), linalool oxide,Z (0.05%), terpinolene (0.22%), sabinene hydrate,E (0.02%), linalool (59.66%), hotrienol (0.10%), 1,3,8-p-menthatriene (0.07%), 6-camphenol (0.04%), camphor (3.15%), chrysanthenol,E (0.03%), bicyclo[2.2.1] heptan-3-one,6,6-dimethyl, 2-methylene (0.29%), borneol (0.37%), p-mentha-1,5-dien-8-ol (0.65%), neoiso-isopulegol (0.09%), α-terpinen-4-ol (0.07%), α-terpineol (0.58%), myrtenal (0.11%), 2,6-dimethyl-3,5,7-octatrien-2-ol,Z,Z (0.64%), 2,6-dimethyl-3,5,7-octatrien-2-ol,E,E (1.65%), citronellol (0.02%), neral (citral b) (0.15%), geranial (citral a) (0.14%), isobornyl acetate (0.07%),δ-elemene (0.03%), exo-2-hydroxycineolacetate (0.11%), α-cubebene (0.01%), α-copaene (0.19%), β-bourbonene (0.17%), β-elemene (1.32%), α-gurjenene (0.023%), β-caryophyllene (2.84%), β-copaene (0.11%), γ-elemene (0.91%), α-humulene (0.364%), β-farnesene,E (0.21%), allo-aromadendrene (0.17%), α-amorphene (0.11%), germacrene D (3.78%), 4-epi-cubebol (0.09%), bicyclogermacrene (0.14%), α-muurolene (0.23%), 10-epi-cubebol (0.42%), γ-cadinene (0.36%), nerolidol,Z (0.18%), macrene D-4-ol (1.05%), caryophyllene oxide (0.62%), viridiflorol (0.06%), muurolol (0.21%), 14-hydroxy-9-epi-caryophyllene,E (0.17%), α-cadinol (0.25%), 14-hydroxy-α-muurolene (0.26%).
Parodi et al., 2012	Rio Grande do Sul, Brazil	Hydrodistillation in a Clevenger-type apparatus	Linalool (59.66%) and 1,8-cineole (9.11%).
Hatano et al., 2012	Ceará, Brazil	Steam distillation in a unspecified apparatus	Alpha-thujene (0.28%), Alpha-pinene (0.21%), Sabinene (2.98%), Myrcene (0.46%), Limonene (23.13%), Beta-ocimene (Z) (0.43%), Gamma-terpinene (0.70%), Terpinen-4-ol (0.22%), Carvone (54.57%), Isopiperitenone (0.97%), Beta-bourbonene (0.73%), Beta-cubebene (0.26%), Beta-elemene (0.28%), Caryophyllene (E) (0.34%), Aromadendrene Allo (0.26%), Alpha-muurolene (4.84%), Gamma-cadinene (0.24%), Geracrene A (0.41%), Bulnesol (2.14%).
Conde et al., 2011	Minas Gerais, Brazil	Essential oil extracted by hydrodistillation in a Clevenger-type apparatus. Hydroethanolic extract preparation in maceration of 70% ethanol for 10 days.	Santolina triene (9.28%), Delta-carene (0.47%), p-Cimene (0.32%), Limonene (0.27%), Beta-phellandrene (0.17%), Cis-ocimene (0.40%), Trans-ocimene (1.56%), Moslene (0.13%), Linalool oxide cis (0.12%), Camphenol (0.47%), Karahanaenone (0.25%), Isopulegol (0.23%), Borneol (0.12%), Dihidrocarvone (0.34%), Mirtenal (0.25%), Carvenone (20.92%), Geranial (24.64%), Trans-carvyl acetate (0.20%), Linalool isobutirate (0.48%), Daucene (0.17%), Beta-cubebene (0.43%), Beta-elemene (2.34%), Alfa-santalene (8.27%), Beta-gurjunene (0.16%), Alloaromadendrene (0.44%), Guaiene (3.62%), Trans-beta-guaiene (0.18%), Beta-bisabolene (0.45%), Delta-cadinene (0.61%), Trans-nerolidol (0.42%), Caryophyllene oxide (1.40%), 1-Hexadecene (0.34%), Octadecane (0.34%).

Authors/year	Plant collection	Extraction method	Chemical composition
da Cunha et al., 2011	Rio Grande do Sul, Brazil	Hydrodistillation in a Clevenger-type apparatus	Not investigated
da Cunha et al., 2010	Rio Grande do Sul, Brazil	Steam distillation in a Clevenger-type apparatus	Not investigated
Neto et al., 2009	São Paulo, Brazil	Essential oil extracted by hydrodistillation in a Clevenger-type apparatus. Hydroethanolic extract preparation in maceration of 80% ethanol for 72h.	LP1: Linalool (77.95%), Carvone (5.53%), 1,8-Cineole (8.80%), Transcaryophyllene (2.13%); LP2: Geranial (33.49%),
Hennebelle et al., 2008	Guadeloupe, France	Extracted sequentially with dichloromethane, methanol, and a methanol/water mixture. After, subjected to liquid-liquid extractions and chromatographic purifications to isolate compounds.	Not investigated
Zétola et al., 2002	Santa Catarina, Brazil	Percolation with 40%, 60% or 80% ethanol and maceration for 24h with 12% of the solvent volume	Only flavonoids
Viana et al., 2000	Ceará, Brazil	Steam distillation in a steam-generator apparatus by Craveiro et al, (1976)	Not investigated
Vale et al., 1999	Ceará, Brazil	Steam distillation in a unspecified apparatus	Not investigated
Viana et al., 1998	Ceará, Brazil	Steam distillation in a unspecified apparatus	Not investigated
